# Intrahepatic T cell analysis reveals Th17 and MAIT17 enrichment in primary sclerosing cholangitis

**DOI:** 10.1016/j.jhepr.2026.101857

**Published:** 2026-04-17

**Authors:** Lisa R.V. Brynjulfsen, Hesham ElAbd, Jonas Øgaard, Markus S. Jördens, Amber G. Bozward, Daniel Kearns, Xiaojun Jiang, Trine Folseraas, Palak J. Trivedi, Ye H. Oo, Andre Franke, Tom H. Karlsen, Espen Melum, Brian K. Chung

**Affiliations:** 1 Norwegian PSC Research Center, Department of Transplantation Medicine, Division of Surgery and Specialized Medicine, Oslo University Hospital Rikshospitalet, Oslo, Norway; 2 Research Institute of Internal Medicine, Division of Surgery and Specialized Medicine, Oslo University Hospital Rikshospitalet, Oslo, Norway; 3 Institute of Clinical Medicine, Faculty of Medicine, University of Oslo, Oslo, Norway; 4 Institute of Clinical Molecular Biology, University of Kiel and University Hospital Schleswig-Holstein (UKSH), Kiel, Germany; 5 Department of Gastroenterology, Hepatology and Infectious Diseases, Medical Faculty, Heinrich Heine University Düsseldorf, University Hospital Düsseldorf, Düsseldorf, Germany; 6 Department of Immunology and Immunotherapy, School of Infection, Inflammation and Immunology, College of Medicine and Health, University of Birmingham, Birmingham, UK; 7 NIHR Biomedical Research Centre, Centre for Liver and Gastrointestinal Research, College of Medicine and Health, University of Birmingham, Birmingham, UK; 8 Centre for Rare Diseases, European Reference Network on Hepatological Diseases (ERN-RARE-LIVER) Centre, Birmingham, UK; 9 Liver Unit, University Hospitals Birmingham National Health Service Foundation Trust Queen Elizabeth, Birmingham, UK; 10 Section of Gastroenterology, Department of Transplantation Medicine, Division of Surgery and Specialized Medicine, Oslo University Hospital Rikshospitalet, Oslo, Norway; 11 Hybrid Technology Hub, Institute of Basic Medical Sciences, University of Oslo, Oslo, Norway

**Keywords:** Primary sclerosing cholangitis, Primary biliary cholangitis, Alcohol-related liver disease, Single-cell RNA sequencing, T cell receptor sequencing, IL-17 producing helper T cells, mucosal-associated invariant T cells

## Abstract

**Background & Aims:**

Primary sclerosing cholangitis (PSC) is an immune-mediated liver disease where the exact contribution of T cells is unclear. To deeply characterize intrahepatic T cells in PSC, we combined single-cell RNA sequencing (scRNA-seq) with T cell receptor sequencing (TCR-seq) and functional confirmation, using primary biliary cholangitis (PBC) and alcohol-related liver disease (ALD) as disease controls.

**Methods:**

Intrahepatic T cells from 12 PSC, nine PBC and three ALD explants were enriched by negative selection, viability-sorted and analyzed by scRNA-seq for total gene and TCR expression. T helper 17 (Th17) and IL-17^+^ mucosal-associated invariant T cell (MAIT17) proportions were analyzed by flow cytometry and liver immunohistochemistry. Liver TCRs in blood were evaluated in an independent PSC, PBC and healthy cohort previously analyzed by TCR β-chain sequencing. Antigenicity of MAIT TCR-transduced reporters was assessed using MAIT-specific tetramers (5-OP-RU-MR1) and MR1^+^H69 cholangiocyte co-cultures.

**Results:**

ScRNA-seq of 40,897 intrahepatic T cells revealed increased Th17/MAIT17 proportions in PSC livers compared with PBC (1.9-fold) and ALD (3.5-fold). Liver Th17 and MAIT17 expressed tissue-resident markers (*CD69*, *CXCR6*, *ITGAE)* and relative abundances measured by scRNA-seq correlated with proportions detected by flow cytometry. Immunohistochemistry liver staining of a validation cohort (n = 37) showed higher fractions of RORγt^+^ cells in PSC and greater cumulative frequencies of liver Th17 and MAIT17 TCR clonotypes in PSC blood compared with PBC. Seven of ten TCR reporters representing the most-common and expanded MAIT17 clonotypes in PSC livers bound 5-OP-RU-MR1 tetramer and were activated by 5-OP-RU-loaded MR1^+^H69 cholangiocytes.

**Conclusions:**

Multimodal analysis of intrahepatic T cells in PSC revealed a novel enrichment of Th17 and MAIT17 which may reflect increased IL-17 polarization of T cells within the PSC microenvironment.

**Impact and implications:**

PSC is a progressive cholestatic biliary disease featuring liver T cell infiltration of unknown significance. To better define the role of T cells in PSC, we phenotyped intrahepatic T cells by scRNA-seq and detected increased proportions of tissue-resident Th17 and MAIT17 cells in PSC relative to disease controls. TCR sequencing also revealed that liver Th17 and MAIT17 TCR clonotypes were more frequent in blood from individuals with PSC compared with PBC. Collectively our results suggest that PSC is associated with a greater expansion of liver Th17 and MAIT17 which may contribute to the pathogenesis of PSC.

## Highlights

• Th17 and MAIT17 cells are enriched in PSC livers compared to disease controls.
• Liver Th17 and MAIT17 express markers associated with inflammation (*KLRB1*, *CCR6*, *RORC*, *IL23R*) and tissue-residency (*CD69*, *CXCR6*, *ITGAE*).
• Liver Th17 and MAIT17 TCR clonotypes are more frequent in blood of individuals with PSC than PBC cholestatic controls.
• Expanded MAIT17 TCR clonotypes recognize the known bacterial metabolite 5-OP-RU.

## Graphical abstract

**Figure:**
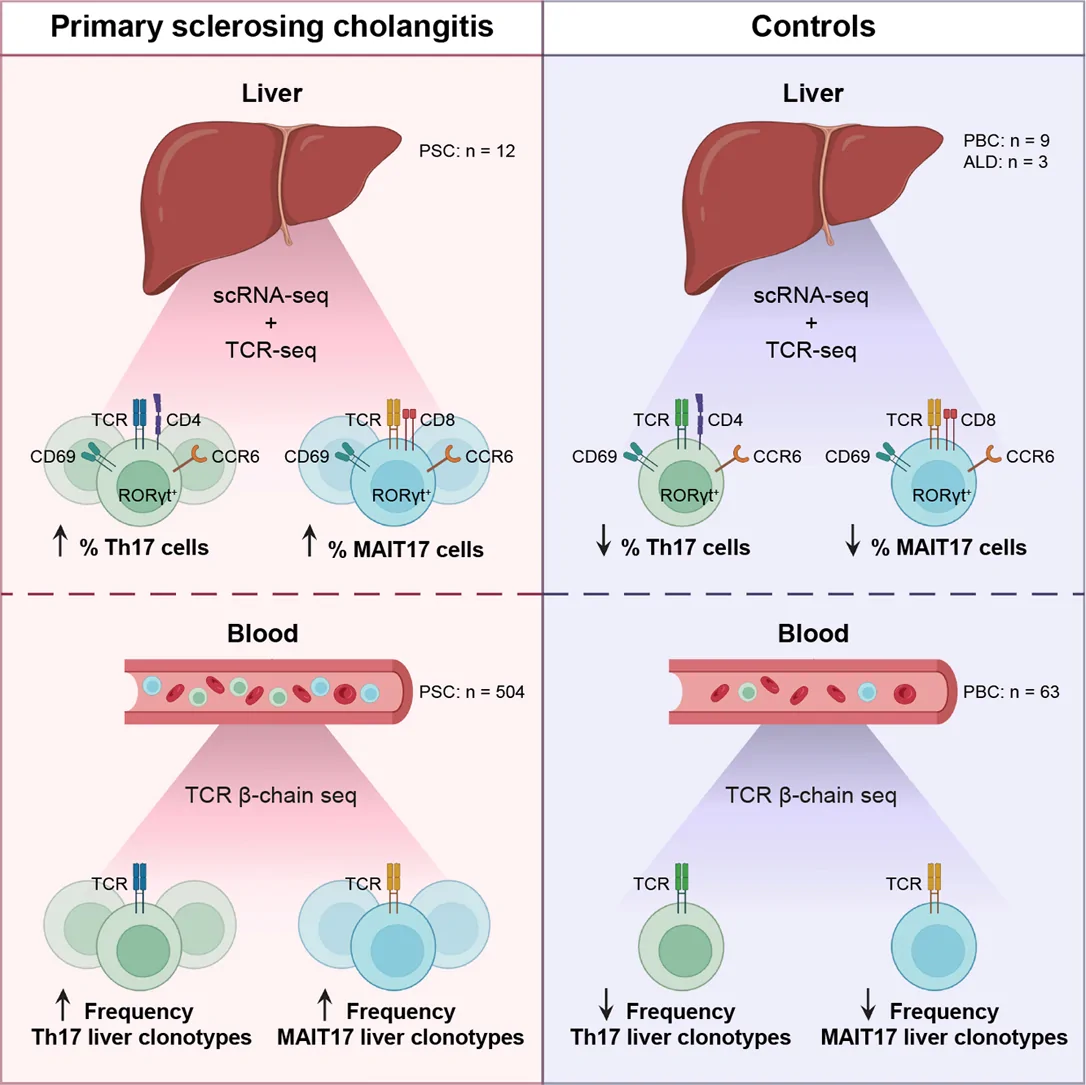

## Introduction

Primary sclerosing cholangitis (PSC) is an inflammatory biliary disorder with no effective medical treatments limiting the progression of disease. Though rare (0–34.7/100,000 individuals globally),^1^ individuals with PSC represent a serious unmet clinical need as they often require liver transplantation, experience reduced quality of life and are at significant risk of cancer development particularly cholangiocarcinoma.^2^ Typical features of PSC include fibrosis, bile duct strictures, and cirrhosis, and 60–80% of patients have a concurrent inflammatory bowel disease (IBD).^1^^,^^3^ Strong genetic associations to variants encoding the human leukocyte antigen complex, which present antigens to T cells, and genes involved in T cell function (*e.g. CCL20*, *CD28*, *IL2RA*, *IL2*, *IL21*) have been repeatedly reported and collectively suggest that T cells are critical in PSC pathogenesis.^3^ Activating T cell antigens in PSC are unknown but reports of a substantial T cell and B cell overlap among the gut and liver compartment suggests that antigens circulating among the gut–liver axis may drive lymphocyte expansion in both microenvironments.^4^^,^^5^

Previous studies on intrahepatic T cell subsets in PSC have found tissue-resident CD8^+^ T cells to be the most abundant lymphocyte subset overall^6^ whereas IL-17^+^CD4^+^ T cells (Th17) are the most prevalent tissue-resident CD4^+^ effector memory (EM) subset within PSC bile ducts.^7^ Regulatory T cells (Tregs) involved in dampening inflammation are reportedly decreased in PSC liver explants compared with primary biliary cholangitis (PBC)^8^ and CD4^+^CD28^−^ T cells associated with chronic antigen and cytokine activation are elevated in PSC livers compared to non-PSC controls.^9^ These previous studies support the earlier genetic findings that T cells are involved in PSC pathogenesis but were biased towards known cell types as the primary methodologies utilized (*e.g.* flow cytometry and immunohistochemistry [IHC]) require the preselection of relative few lineage markers that may have limited the analysis to known cell types and features.

To improve upon previous studies, recent immunophenotyping efforts have leveraged more granular RNA technologies, including single-cell RNA sequencing (scRNA-seq), single-nuclei RNA seq (snRNA-seq) and spatial transcriptomics.^10^^,^^11^ These technologies enable deeper global profiling of the entire transcriptome, increasing phenotyping precision and reducing preselection bias compared with historical approaches.^12^ Using a combination of scRNA-seq and flow cytometry to define T cell subsets, Poch and Krause *et al.*^13^ identified a liver-resident population of naïve-like CD4^+^ T cells expanded in PSC that preferentially differentiate into Th17 cells under polarizing *in vitro* conditions. Andrews and Nakib *et al.*^14^ applied several multiomic modalities (scRNA-seq, snRNA-seq, spatial transcriptomics) to profile the entire liver compartment in patients with PSC and disease controls and also showed an expansion of CD4^+^ T cells that co-localized with myeloid subsets. Although the main findings in these studies were not immunological, these data suggest that specific CD4^+^ T cells have a role in pathogenesis but in both studies, scRNA-seq was implemented on a limited number of disease controls and protein-level tissue confirmation and functional validation of specific T cell clonotypes were not investigated.

As identification of enriched T cell subsets and confirmation of their antigen reactivity may broaden our understanding of the pathophysiological mechanisms underlying PSC, we aimed to determine if T cell subsets in PSC livers are enriched compared with disease controls by leveraging scRNA-seq, T cell receptor sequencing (TCR-seq), flow cytometry, and liver IHC. We further assessed if liver T cell clonotypes are detected in peripheral circulation using PSC, PBC, and healthy blood samples previously analyzed by T cell receptor (TCR) β-chain sequencing^15^ and if expanded liver T cell clonotypes found across multiple individuals with PSC recognize a common antigen.

## Patients and methods

### Patient samples

Liver sections from individuals undergoing liver transplantation with PSC (n = 12), PBC (n = 9), and ALD (n = 3) were collected immediately following explantation for isolation of liver mononuclear cells (LMC). Fixed explant liver tissue from PSC (n = 20), PBC (n = 10), alcohol-related liver disease (ALD, n = 4) and metabolic dysfunction-associated steatohepatitis (MASH, n = 3) were analyzed by IHC. Blood samples from PSC (n = 504), PBC (n = 63), and healthy controls (n = 904) were assessed by TCR β-chain sequencing. This study was approved by the Regional Committees for Medical and Health Research Ethics of South East Norway (reference numbers: 15368 and 18221) and by the local ethical committees at the University of Birmingham (LREC #06-Q2702-61) and University of Kiel (D441/16, D474/12, A161/08, A103/14, and A148/14) and conducted in accordance with the Declaration of Helsinki and Istanbul. All study participants provided written informed consent before study inclusion.

### ScRNA-seq and TCR-seq

ScRNA-seq and TCR-seq was performed using Chromium Next GEM Single Cell 5′ version 1.1 kits or Chromium Next GEM Single Cell 5' v2 kits following the manufacturer’s instructions (User guide CG000207 Rev E and User guide CG000331 Rev D, 10x Genomics, Pleasanton, CA, USA) and batch corrected for kit variations (see Supplementary Materials & Methods). Enriched T cells (8,280–15,000 per reaction) were loaded per channel onto Chromium Next GEM G or K chips for GEM generation using a Chromium X/iX (10x Genomics). cDNA libraries were sequenced on a NovaSeq (Illumina, San Diego, CA, USA) using SP or S4 flow cells at the recommended minimum sequencing depth of 20,000 reads per cell for total gene libraries and 5,000 read pairs per cell for TCR libraries. Sequencing data from scRNA-seq can be explored at RIIM Data Explorer (https://riim-data.no/index2.html).

### Liver TCR-seq

For TCR-seq of liver T cells, filtered contig annotation outputs from the Cell Ranger multi pipeline (10x Genomics) containing single and paired TCR αβ chain sequences were assigned to individual patients and scRNA-seq T cell clusters using R version 4.3.2 (R Foundation for Statistical Computing, Vienna, Austria) and Immunarch version 0.9.0.^17^ Clonotypes were defined by the amino acid sequence of the TCR αβ chain complementarity-determining region 3 (CDR3). Expanded clonotypes were defined as cells expressing the identical TCR clonotype detected in at least two cells. For each cluster, the percentage of expanded cells was calculated as the number of expanded cells divided by the total number of TCR per sample.

### Statistical analysis

All values shown represent the mean ± standard deviation unless otherwise stated. Mann-Whitney *U* test was used for comparisons between two groups and Kruskal-Wallis test followed by Dunn’s test was used for comparisons with three groups. Values of *p* ≤0.05 for Mann-Whitney *U* test and *p* values adjusted for Dunn’s multiple comparisons ≤ 0.05 were considered statistically significant. Correlations and *p* values between scRNA-seq Th17 and *IL17*^+^ mucosal-associated invariant T cell (MAIT17) frequencies and flow cytometry were calculated using two-tailed Pearson analysis. Unless otherwise stated, statistical analyses were performed using Prism version 10.0.3 (GraphPad, San Diego, CA, USA).

## Results

### Expansion of Th17/MAIT17 cells in livers of individuals with PSC

**Fig. 1:**
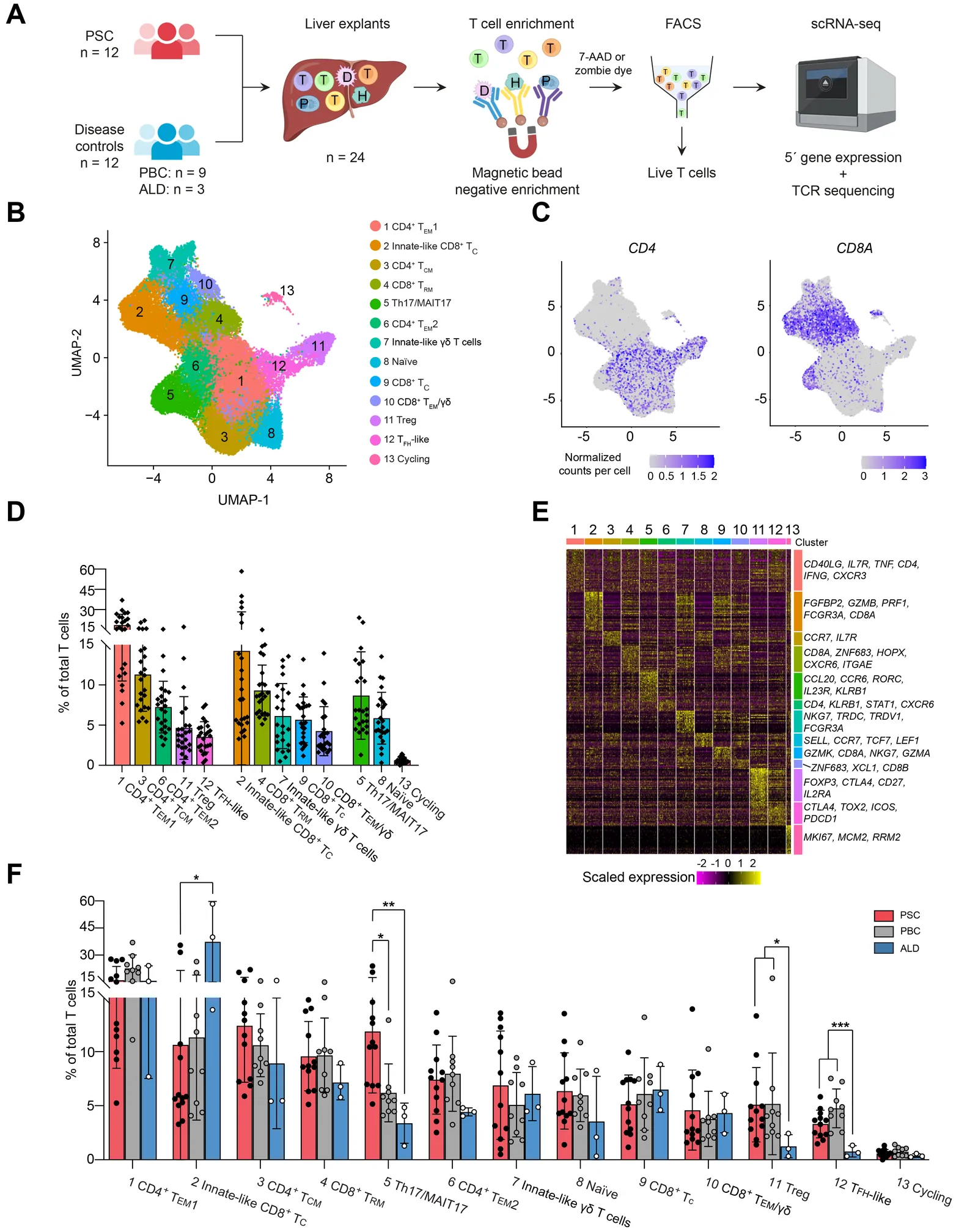
Expansion of Th17/MAIT17 cells in livers of individuals with PSC. (A) Workflow for scRNA-seq of T cells from explanted livers of PSC (n = 12), PBC (n = 9) and ALD (n = 3). (B) UMAP of 40,897 T cells from all livers combined (PSC: 16,641 cells, PBC: 18,426 cells, ALD: 5,830 cells). (C) T cell UMAPs of *CD4* or *CD8A* expression. (D) Relative abundance of each T cell cluster relative to total T cells ordered into *CD4*^+^ clusters, *CD8*^+^ clusters, and clusters with both *CD4*^+^ and *CD8*^+^ cells. (E) Heatmap of top 40 differentially expressed genes for each cluster. Representative marker genes shown on the right. (F) Relative abundance of T cell clusters between PSC, PBC, and ALD, and between PSC + PBC (PSC and PBC merged into single disease group) and ALD. Comparisons between PSC + PBC and ALD are highlighted in bold. Statistical significance was evaluated using Kruskal-Wallis test followed by Dunn’s test for comparison between three groups and Mann-Whitney *U* test for comparison between two groups, ∗*p* ≤0.05, ∗∗*p* ≤0.01, ∗∗∗*p* ≤0.001. ALD, alcohol-related liver disease; D, dendritic cell; H, hepatocyte; MAIT17*, IL17*^+^ mucosal-associated invariant T cell; P, plasma cell; PBC, primary biliary cholangitis; PSC, primary sclerosing cholangitis; scRNA-seq, single-cell RNA sequencing; T, T cell; T_c_, cytotoxic T cell; T_CM_, central memory T cell; T_EM_, effector memory T cell; T_FH_-like, follicular-helper like T cell; Th17, T helper type 17; Treg, regulatory T cell; T_RM_, tissue-resident memory T cell; UMAP, uniform manifold approximation and projection.

To investigate the phenotype of intrahepatic T cells in PSC (n = 12) compared with disease controls (PBC: n = 9, ALD: n = 3; Table 1; Table S1), we enriched T lymphocytes from LMC and assessed total gene and TCR expression using 5′ scRNA-seq and TCR-seq (Fig. 1A). In total, 40,897 intrahepatic T cells passed quality control and clustering by differential gene expression revealed 13 T cell subsets (Fig. 1B) that coarsely segregated based on *CD4* (cluster 1, 3, 6, 11, 12), *CD8A* (cluster 2, 4, 7, 9, 10), or a combination of *CD4* and *CD8A* expression (cluster 5, 8, 13; Fig. 1C). Clustering of T cell subsets was consistent between both PSC and control patients with each cluster identified in every liver except cluster 13 (cycling) which was present in 23 of 24 explants (Fig. 1D; Fig. S4A).

**Table 1:** Clinical overview of the scRNA-seq cohort at time of liver transplantation.

	PSC (n = 12)	PBC (n = 9)	ALD (n = 3)
Age	42.6 (19–70)	51.3 (43–69)	57.7 (55–62)
Males, n (%)	10 (83.3%)	3 (33.3%)	0 (0.0%)
Bilirubin, μmol/L	175 (8–916)	59 (11–166)	45 (14–102)
INR	1.2 (0.9–1.6)	1.2 (0.9–1.6)	1.5 (1.4–1.7)
ALT, U/L	136 (18–311)	71 (29–192)	33 (26–38)
ALP, U/L	404 (176–1,110)	491 (159–1,776)	152 (120–200)
Albumin, g/dl	38 (30–49)	35 (21–42)	37 (33–40)
Platelets, 10^9^/L	177 (36–375)	120 (46–296)	118 (83–159)

Data are presented as mean (range) or n (%). ALD, alcohol-related liver disease; ALP, alkaline phosphatase; ALT, alanine aminotransferase; IBD, inflammatory bowel disease; INR, international normalized ratio; PBC, primary biliary cholangitis; PSC, primary sclerosing cholangitis.

Using canonical lineage markers from differential gene expression analysis (Fig. 1E), CD4^+^ T cell subsets were annotated into CD4^+^ EM1 (T_EM_1), CD4^+^ central memory (T_CM_), CD4^+^ T_EM_2, Tregs, and T follicular-helper (T_FH_)-like cells whereas CD8^+^ T cell subsets were annotated into innate-like CD8^+^ cytotoxic (T_c_), CD8^+^ tissue-resident memory (T_RM_), innate-like γδ T cells, CD8^+^ T_c_, and CD8^+^ T_EM_/γδ cells. Of the three subsets containing a mixture of both CD4^+^ and CD8^+^ cells, cluster 5 expressed *CCR6*, *RORC*, *IL23R*, and *KLRB1* indicative of both Th17 and MAIT17 cells whereas the two remaining clusters were classified as naïve and cycling.

After defining the intrahepatic T cell compartment in the explanted livers, we examined the relative proportions of each T cell subset in PSC livers compared with disease controls (Fig. 1F). Cluster 5 containing Th17 and MAIT17 cells was increased 1.9-fold in PSC (11.9 ± 5.7%) compared with PBC (6.2 ± 2.7%, *p* = 0.023) and 3.5-fold higher in PSC compared with ALD (3.4 ± 1.9%, *p* = 0.0048). Relative proportions of Th17/MAIT17 in PSC livers were also elevated *vs*. healthy controls previously analyzed by scRNA-seq (PSC: 10.2 ± 3.5%, healthy controls: 4.4 ± 3.4%, *p* = 0.0069)^14^ and the inclusion of eight PSC and two PBC liver samples from Andrews and Nakib *et al.*^14^ further confirmed that Th17/MAIT17 abundances are increased in PSC livers compared with PBC disease controls (Fig. S5). In contrast, cluster 2 (innate-like CD8^+^ T_c_) was significantly reduced in PSC compared with ALD (PSC: 10.6 ± 10.9%, ALD: 37.4 ± 22.3%, *p* = 0.041) but not PBC (11.3 ± 7.7%, *p* = 0.14). No other clusters showed significant differences in abundance between PSC, PBC, and ALD by scRNA-seq. To determine if immune-related liver disorders jointly exhibited enrichment of T cell states, we merged the relative abundance values of each T cell cluster from PSC and PBC livers (Fig. 1F) and saw that Tregs (cluster 11) were increased in the immune-related liver diseases (PSC and PBC) compared with ALD (PSC and PBC: 5.1 ± 3.9%, ALD: 1.3 ± 1.1%, *p* = 0.011) and also harbored a greater proportion T_FH_-like cells (cluster 12; PSC and PBC: 3.9 ± 1.7%, ALD: 0.8 ± 0.5%, *p* = 0.0010). Fibrosis-4 (FIB-4) and model for end-stage liver disease-sodium (MELD-Na) scores indicative of fibrosis and liver decompensation at transplantation did not correlate with any of the T cell proportions differentially abundant between PSC, PBC, and ALD livers (Fig. S4B). Collectively these results imply that T cells with an IL-17 signature are expanded in livers of individuals with PSC and that Tregs and T_FH_-like cells, regulating inflammation and promoting B cell differentiation, are enriched in the PSC and PBC compared with ALD.

### Intrahepatic Th17 and MAIT17 cells are distinct and elevated in PSC

**Fig. 2:**
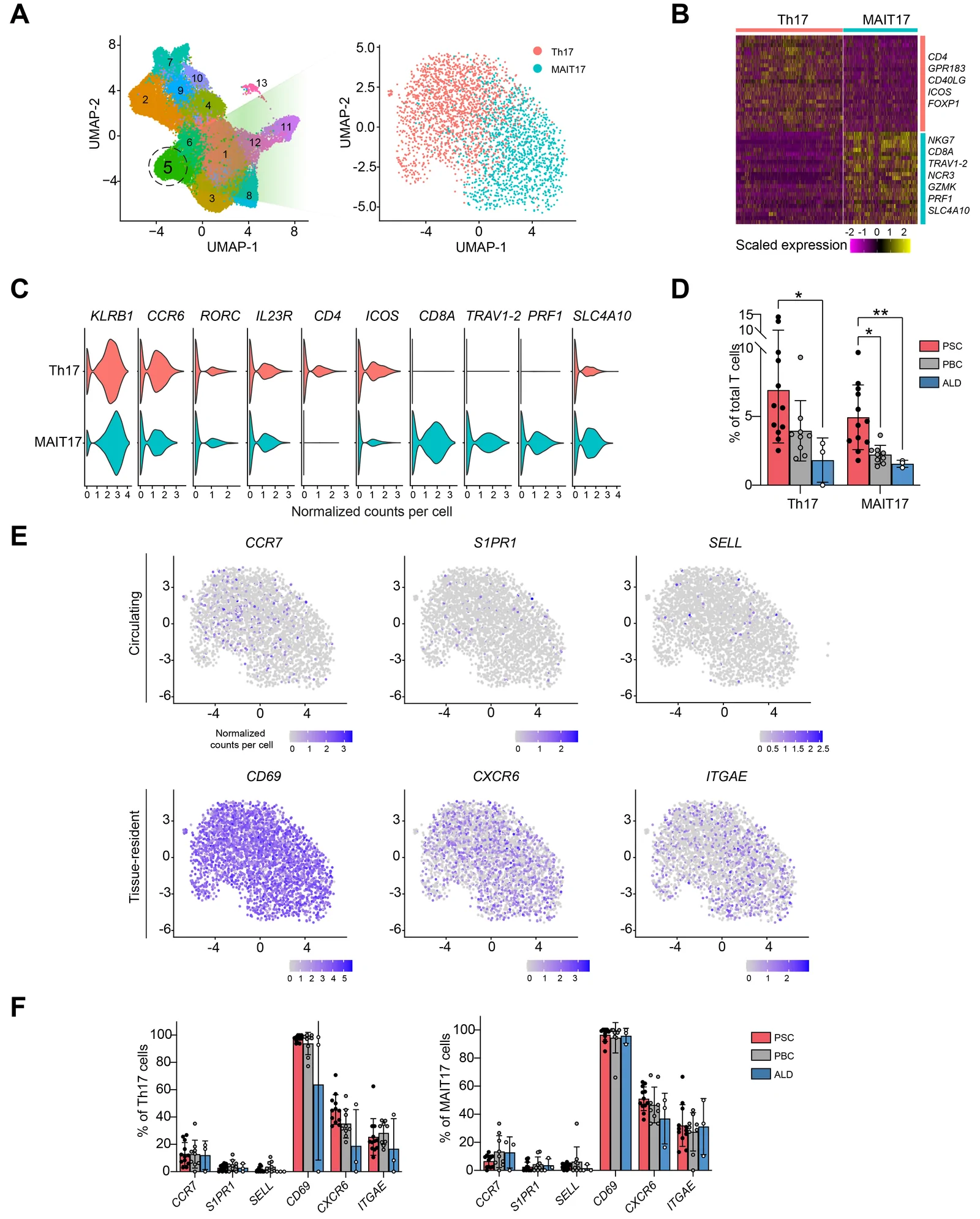
Intrahepatic Th17 and MAIT17 cells are distinct and elevated in patients with PSC. (A) Reclustering of Th17/MAIT17 cluster from the total T cell compartment of PSC, PBC, and ALD explants. Th17/MAIT17 UMAP contains 3,039 cells from PSC (n = 12, 1,746 cells), PBC (n = 9, 1,044 cells), and ALD (n = 3, 249 cells). (B) Heatmap of top 25 differentially expressed genes for Th17 and MAIT17. Representative marker genes shown on the right. (C) Normalized expression counts of indicated genes in Th17 and MAIT17. (D) Abundance of Th17 and MAIT17 among PSC, PBC, and ALD calculated by scRNA-seq. (E) Expression of circulating (*CCR7*, *S1PR1*, *SELL*) and tissue-resident (*CD69*, *CXCR6*, *ITGAE*) markers in Th17/MAIT17 of PSC, PBC and ALD liver explants. (F) Proportion of Th17 and MAIT17 expressing the indicated circulating and tissue-resident markers split by disease. Statistical significance was evaluated using Kruskal-Wallis test followed by Dunn’s test, ∗*p* ≤0.05, ∗∗*p* ≤0.01. ALD, alcohol-related liver disease; MAIT17, *IL17*^+^ mucosal-associated invariant T cell; PBC, primary biliary cholangitis; PSC, primary sclerosing cholangitis; Th17, T helper type 17; UMAP, uniform manifold approximation and projection.

To determine if specific gene profiles for Th17 and MAIT17 cells could be distinguished, we reclustered the combined Th17/MAIT17 cluster (cluster 5) and observed a separation of a *CD4*^+^ Th17 and *CD8*^*+*^ MAIT17 subset (Fig. 2A). Cells in the Th17 cluster featured higher expression of *CD4* and the co-stimulatory receptors *ICOS* and *CD4*0*LG*,^18^ whereas MAIT17 cells expressed the canonical MAIT genes *TRAV1-2* and *SLC4A10*,^19^ and the T_c_-related markers *NKG7*, *GZMK*, and *PRF1* (Fig. 2B).^20^^,^^21^ Despite these clear differences, Th17 and MAIT17 cells shared a large phenotypic similarity including a robust expression of the proinflammatory IL-17^+^ T cell markers *KLRB1*, *CCR6*, *RORC*, and *IL23R* (Fig. 2C). Abundance analysis (Fig. 2D) showed that Th17 cells were enriched in PSC livers compared with ALD (PSC: 6.9 ± 3.9%, ALD: 1.8 ± 1.6%, *p* = 0.020) but not compared with PBC livers (PBC: 4.0 ± 2.2%, *p* = 0.13) whereas MAIT17 cells were more abundant in PSC livers (4.9 ± 2.3%) compared with both PBC (2.2 ± 0.7%, *p* = 0.021) and ALD (1.6 ± 0.3%, *p* = 0.0061). Weak expression of the circulating T cell markers *CCR7*, *S1PR1*, and *SELL* was detected in a small proportion of Th17/MAIT17 cells whereas higher levels of the tissue-resident markers *CD69*, *CXCR6*, and *ITGAE* were observed in larger proportion of the Th17/MAIT17 cluster (Fig. 2E).^6^ Th17/MAIT17 cluster split by disease revealed no difference in the proportions of Th17/MAIT17 expressing circulating or tissue-resident markers between PSC, PBC, and ALD livers (Fig. 2F). TCR-seq analysis of clonotypes defined by scRNA-seq as Th17 and MAIT17 showed a similar percentage of expanded clonotypes and cells assigned to expanded clonotypes among PSC and disease controls (Fig. S6).

### Th17 and MAIT17 analyses using flow cytometry and immunohistochemistry staining

**Fig. 3:**
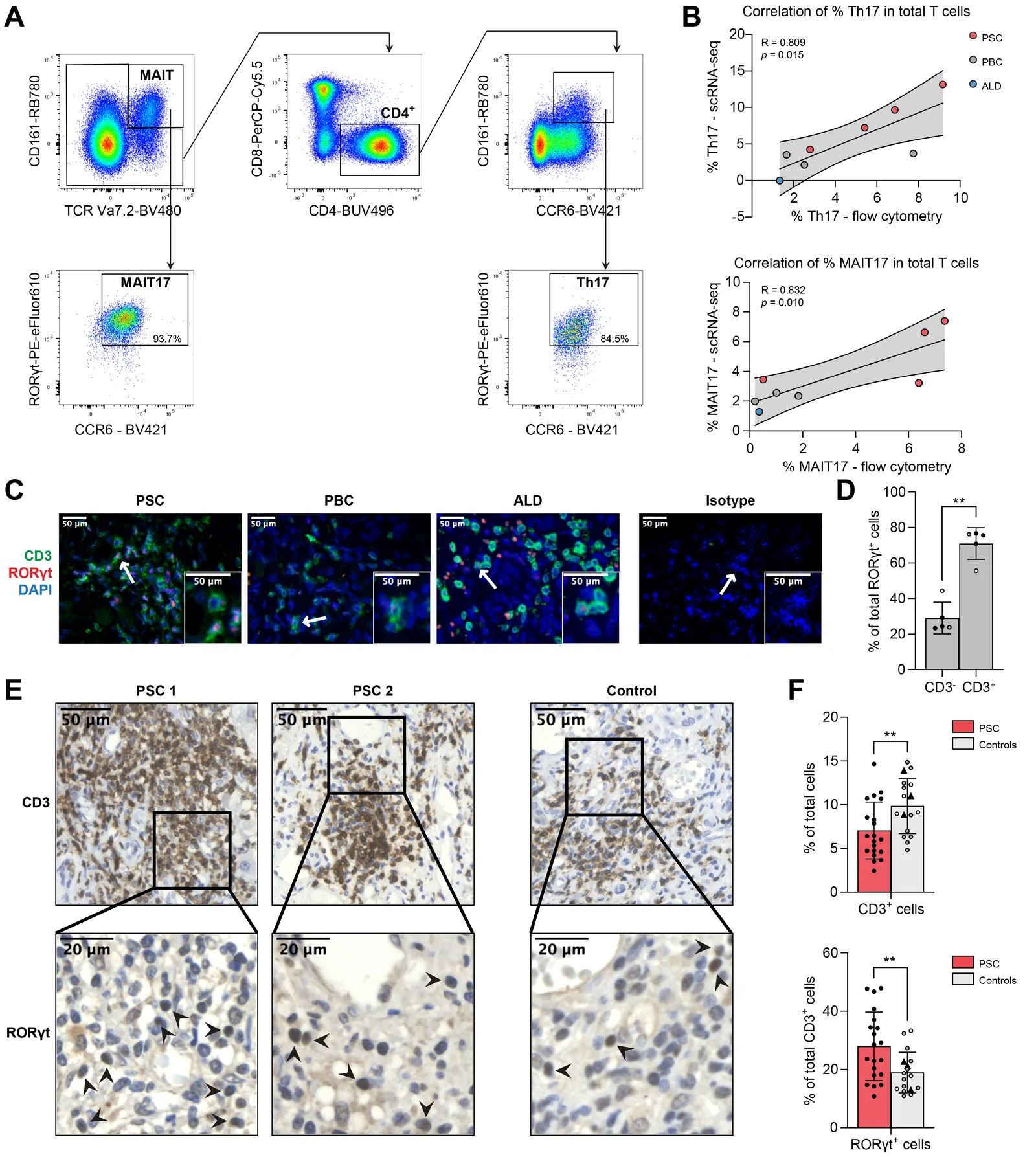
Th17 and MAIT17 analyses using flow cytometry and immunohistochemistry staining. (A) Gating strategy for MAIT17 and Th17 gated from live CD3^+^ TCRγδ^-^ T cells derived from explanted livers of patients (four PSC, three PBC and one ALD). (B) Correlation between Th17 and MAIT17 abundance measured by scRNA-seq and flow cytometry. (C) CD3 and RORγt IF of liver tissue from PSC and disease controls. (D) Relative abundance of CD3^-^RORγt^+^ and CD3^+^RORγt^+^ cells in three PSC (black dots) and two disease controls (PBC: gray dot, ALD: white dot). (E) CD3 and RORγt IHC staining of liver tissue from PSC and disease control. (F) Relative abundance of CD3^+^ cells in total cells and RORγt^+^ cells in CD3^+^ cells between PSC and disease controls (PBC: gray dots, ALD: white dots, MASH: black triangles). Statistical significance of correlation was evaluated using two-tailed Pearson correlation. Mann-Whitney *U* test was used for comparison between two groups, ∗∗*p* ≤0.01. ALD, alcohol-related liver disease; MAIT, mucosal-associated invariant; MAIT17, *IL17*^+^ mucosal-associated invariant T cell; PBC, primary biliary cholangitis; PSC, primary sclerosing cholangitis; scRNA-seq, single-cell RNA sequencing; Th17, T helper type 17.

To assess if Th17 and MAIT17 were increased in PSC livers at the protein level, we first performed flow cytometry on a subset of LMC (n = 8) analyzed by scRNA-seq using CD4^+^CD161^+^CCR6^+^RORγt^+^ as markers for Th17 cells and Vα7.2^+^CD161^+^CCR6^+^RORγt^+^ for MAIT17 cells (Fig. 3A and Fig. S7). We observed a strong correlation in the relative proportion of Th17 cells (R = 0.81, *p* = 0.015) and MAIT17 cells (R = 0.83, *p* = 0.010) measured by flow cytometry and scRNA-seq supporting that liver Th17 and MAIT17 are increased in PSC (Fig. 3B). To confirm that Th17 and MAIT17 were enriched in PSC livers, we next quantified CD3 and RORγt co-expression by immunofluorescence (IF) and noted that the majority of RORγt^+^ cells co-stained with CD3 (Fig. 3C, D; Fig. S8). IHC staining for CD3 and RORγt of 20 PSC and 17 liver disease controls (10 PBC, four ALD, three MASH; Fig. 3E; Table S2) in a validation cohort showed that proportions of CD3^+^ cells per total cells were decreased in PSC compared with disease controls (PSC: 7.1 ± 3.2%, disease controls: 9.9 ± 3.2%, *p* = 0.008; Fig. 3F) but RORγt^+^ cells in CD3^+^ regions were significantly enriched in PSC (PSC: 28.0 ± 11.8%, disease controls: 19.0 ± 7.0%, *p* = 0.009). Collectively these protein-level data support our scRNA-seq findings that relative proportions of RORγt^+^ cells are increased in PSC compared with disease controls.

### Th17 TCR clonotypes and MAIT17 cells in the blood of individuals with PSC

**Fig. 4:**
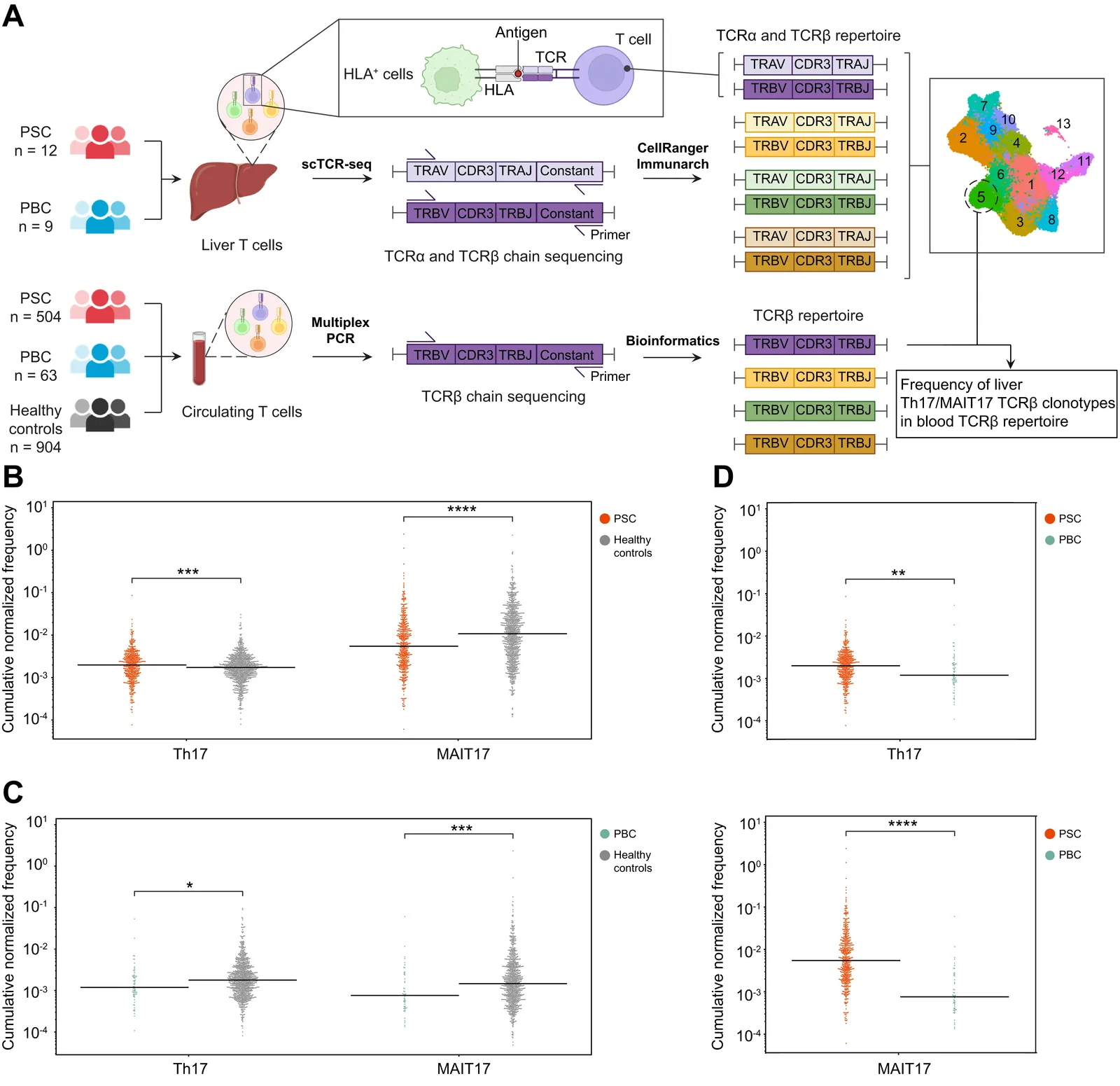
Identification of PSC and PBC Th17/MAIT17 liver clonotypes in blood of individuals with PSC, PBC, and HC. (A) Workflow for identifying proportions of Th17 and MAIT17 liver TCR clonotypes shared with blood T cell repertoires of patients with PSC (n = 504), PBC (n = 63), and healthy controls (n = 904). (B) Cumulative normalized frequency of PSC liver Th17 and MAIT17 TCR clonotypes found in blood of PSC and healthy controls. (C) Cumulative normalized frequency of PBC liver Th17 and MAIT17 TCR clonotypes found in blood of PBC and healthy controls. (D) Comparison of PSC liver Th17/MAIT17 clonotypes in PSC blood *vs*. PBC liver Th17/MAIT17 clonotypes in PBC blood. Statistical significance was evaluated by Mann-Whitney *U* test in Python using statannotations package, ∗*p* ≤0.05, ∗∗*p* ≤0.01, ∗∗∗*p* ≤0.001, ∗∗∗∗*p* ≤0.0001. MAIT17, IL-17+ mucosal-associated invariant T cell; ns, non-significant; PBC, primary biliary cholangitis; PSC, primary sclerosing cholangitis.

Having confirmed increased Th17 and MAIT17 cells in PSC livers, we determined if the abundance of Th17 and MAIT17 clonotypes defined by TCR-seq were altered in blood samples from an independent cohort of PSC (n = 504), PBC (n = 63), and healthy controls (HC, n = 904) previously assessed by TCR β-chain sequencing (Fig. 4A).^15^ We detected 218 of 843 total PSC Th17 liver clonotypes (25.9%) and 156 of 494 total PSC MAIT17 liver clonotypes (31.6%) across the TCR blood repertoire. Liver Th17 clonotypes constituted a greater median cumulative frequency of the blood TCR repertoire in PSC (PSC: 1.99 × 10^-3^, HC: 1.74 × 10^-3^, *p* = 3.05 × 10^-4^, Fig. 4B) whereas cumulative median frequencies of liver MAIT17 clonotypes in PSC blood were dramatically reduced in the peripheral TCR repertoire of HC (PSC: 0.5 × 10^-2^, HC: 1.08 × 10^-2^, *p* = 2.08 × 10^-11^).

In contrast to PSC, we detected 122 of 492 total PBC Th17 liver clonotypes (24.8%) in PBC blood samples (n = 63) and 71 of 252 total PBC MAIT17 liver clonotypes across the TCR blood repertoire of HC (n = 904). Median cumulative frequencies of PBC Th17 and MAIT17 liver clonotypes were both lower in PBC blood than HC (Th17 PBC: 1.25 × 10^-3^, HC: 1.70 × 10^-3^, *p* = 1.06 × 10^-2^; MAIT17 PBC: 6.4 × 10^-4^, HC: 1.32 × 10^-3^, *p* = 7.02 × 10^-4^; Fig. 4C). Interestingly, PSC Th17 and MAIT17 liver clonotypes were significantly elevated in PSC blood compared with PBC Th17 and MAIT17 liver clonotypes in PBC blood. (Th17 PSC: 1.99 × 10^-3^, Th17 PBC: 1.25 × 10^-3^, *p* = 1.07 × 10^-3^; MAIT17 PSC: 0.5 × 10^-2^, MAIT17 PBC: 6.4 × 10^-4^, *p* = 4.81 × 10^-16^; Fig. 4D). No difference in total MAIT TCR frequencies was detected between PSC and PBC blood samples using a deep learning MAIT predictive model (Seq2MAIT)^22^ whereas both disease groups showed lower MAIT TCR frequencies than HC (PSC: 0.83 × 10^-2^, PBC: 0.80 × 10^-2^, HC: 1.01 × 10^-2^; Fig. S9).

### Expanded MAIT17 TCR clonotypes in PSC livers recognize 5-OP-RU

**Fig. 5:**
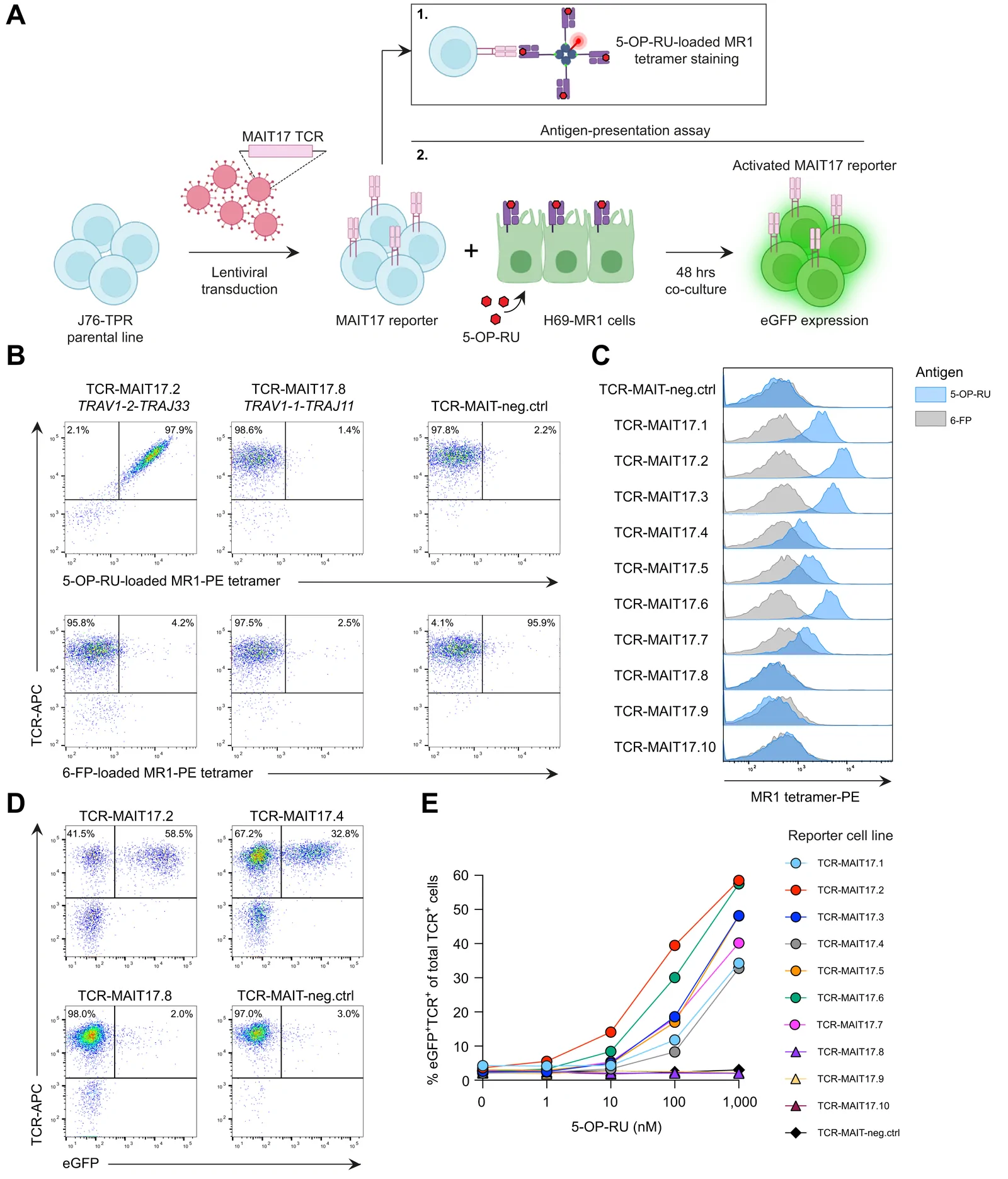
Expanded MAIT17 TCR clonotypes in PSC livers recognize 5-OP-RU. (A) Generation, tetramer validation, and activation of MAIT17 TCR reporters. (B) 5-OP-RU-MR1 tetramer staining of MAIT17 TCR reporters generated from TCR-seq of liver T cells. Plots show staining of MAIT17 TCR cell lines expressing prototypical MAIT TCR (*TRAV1-2*-*TRAJ33*), non-prototypical MAIT TCR (*TRAV1-1*-*TRAJ11*) and non-MAIT TCR (TCR-MAIT-neg.ctrl). (C) 5-OP-RU-MR1 and 6-FP-MR1 (negative) tetramer staining overlay of MAIT17 TCR reporters (n = 10). (D) NFAT-eGFP expression indicates MAIT17 TCR activation by 5-OP-RU-pulsed H69-MR1 cholangiocytes. (E) 5-OP-RU activation of all MAIT17 TCR reporters. 5-OP-RU, 5-(2-oxopropylideneamino)-6-d-ribitylaminouracil; 6-FP, 6-formylpterin; eGFP, enhanced green fluorescent protein; J76-TPR, Jurkat 76 triple parameter reporter; MAIT17, *IL17*^+^ mucosal-associated invariant T cell; MR1, major histocompatibility complex class I-related gene protein; TCR, T cell receptor.

To validate that liver MAIT17 defined by scRNA-seq are *bona fide* MAIT cells that recognize the prototypical MAIT antigen 5-(2-oxopropylideneamino)-6-d-ribitylaminouracil (5-OP-RU), we selected the top 10 most prevalent and highly expanded MAIT17 TCR αβ-CDR3 detected by TCR-seq (Table S3) and generated MAIT17 TCR reporters (MAIT17.1 to MAIT17.10) using lentiviral transduction (Fig. 5A). Seven of 10 MAIT17 TCR (MAIT17.1 to MAIT17.7) utilized known MAIT TCR α-genes (*TRAV1-2*, *TRAJ33*, or *TRAJ12*)^23^ whereas MAIT17.8 to MAIT17.10 utilized TCR genes not previously ascribed to MAIT cells (Table S3). As expected, MAIT17 reporters expressing the prototypic MAIT TCR α-genes stained positive with 5-OP-RU-MR1 tetramer but showed differing binding affinities as measured by tetramer-positive mean fluorescence intensity (MFI) values (Fig. 5B, C). MAIT17 reporters expressing unreported MAIT TCR α-genes did not stain with the 5-OP-RU-MR1 tetramer (MAIT17.8 to MAIT17.10). Tetramer-positive TCR reporters were activated in a dosage-dependent manner by 5-OP-RU-pulsed H69-MR1 cholangiocytes while tetramer-negative reporters remained eGFP negative (Fig. 5D, E). These findings confirm that MAIT cells identified by scRNA-seq express TCR which recognize and are stimulated by 5-OP-RU.

## Discussion

Herein we performed scRNA-seq and TCR-seq on intrahepatic T cells from the largest number of individuals with PSC to date and identified 13 T cell subsets where Th17 and MAIT17 cells were increased and were consistent with findings detected at the protein level using flow cytometry, IF, and IHC staining.

It has previously been reported that Th17 cells are the most abundant CD4 T_EM_ in bile ducts of patients with PSC^7^ and Poch and Krause *et al.*^13^ identified a subset of enriched tissue-resident naïve-like CD4^+^ T cells with the propensity to develop into Th17. Moreover, studies by Jeffery, Hunter and Humphreys *et al.*^24^ suggest CD4 T cells polarize towards Th17 around biliary epithelium and Oo and Banz *et al.*^25^ showed that inflamed and microbe-exposed biliary epithelium secrete CXCL10 and CCR6, recruiting Th17 cells via CXCR3 across hepatic sinusoids. Our findings significantly extend these previous observations and show for the first time that Th17 are directly increased in PSC livers by scRNA-seq, flow cytometry, and IHC. A potential mechanism for this increase could relate to the association of PSC with IBD as peripheral blood mononuclear cells from patients with PSC-IBD stimulated by gut-derived microbiota showed greater Th17 differentiation and IL-17 secretion compared with peripheral blood mononuclear cells from patients with IBD and HC.^26^^,^^27^ Furthermore, increased liver Th17 differentiation has also been observed in gnotobiotic mice inoculated with PSC-derived microbiota suggesting that enrichment of liver Th17 cells in PSC could be caused by disease-associated microbial strains.^28^ Collectively, our data suggest that the gut–liver axis may be a key factor in driving the enrichment of Th17 cells in PSC livers.

In addition to Th17 cells, we also observed that liver MAIT17 cells were increased in PSC. These data support previous observations suggesting that intrahepatic MAIT cells localize around peribiliary regions and are activated by *Escherichia coli* via intrahepatic B cells and biliary epithelium.^29^ These findings however contrast with previous reports showing PSC and non-PSC controls have similar abundances of liver MAIT cells when defined as TCR Vα7.2^+^CD161^+^ by flow cytometry or as TCR Vα7.2^+^ by IHC.^30^^,^^31^ These disparate results could reflect the ability of scRNA-seq to identify MAIT17 cells using multiple markers (*e.g. RORC*, *CCR6*, *IL23R*) instead solely by the expression of TCR Vα7.2 alone. As expression of TCR Vα7.2 is not exclusive to MAIT cells, identification using MR1-5-OP-RU tetramers is regarded as the most accurate TCR-specific method.^32^ Another explanation for our findings is the potential classification of non-MAIT cells as MAIT17 as we saw that three of 10 TCR reporters generated from the MAIT17 cluster defined by scRNA-seq did not recognize the prototypical MAIT antigen. These non-MAIT cells share nearly identical gene profiles with prototypical MAIT17 cells and may therefore represent a MAIT17-like subset which possibly recognize a range of non-MAIT antigens. As MAIT17-like T cells (*TRAV1-2*^–^, *TRAJ33*^–^*, TRAJ12*^–^, and *TRAJ20*^–^) constitute 6.7–100% (2–117 total cells per patient) of the MAIT17 scRNA-seq cluster from different liver explants (data not shown), future large-scale screening of MAIT17-like TCR reporters may reveal the identity of novel antigens that promote IL-17^+^ T cell differentiation.

Th17 and MAIT17 subsets in our data both expressed RORγt which induces transcription of the genes encoding for IL-17.^33^ Although the exact role of IL-17^+^ T cells is still to be determined in PSC, inhibition of RORγt has shown to protect against 3,5-diethoxycarbonyl-1,4-dihydrocollidine-induced liver injury in mice inoculated with mixture of *Klebsiella pneumoniae, Proteus mirabilis*, and *Enterococcus gallinarum*.^28^ Increased circulating Th17 cells have also been reported in rheumatoid arthritis, multiple sclerosis, and psoriasis indicating that IL-17^+^ T cells are a general disease feature of immune-mediated chronic disorders.[34], [35], [36] Our observations here and previous reports suggest that Th17 and MAIT17 depletion may be of potential benefit in PSC but as both cell types participate in the clearance of extracellular pathogens and mucosal homeostasis, targeting of Th17 and MAIT17 in PSC requires caution and further investigation.[37], [38], [39], [40]

To determine if our scRNA-seq results from the liver were generalizable to other sites and a larger study cohort, we assessed the abundance of Th17 and MAIT17 clonotypes from PSC and PBC livers in blood of individuals with PSC, PBC, and HC. PSC liver Th17 clonotypes were enriched in PSC blood *vs*. HC similar to other immune-mediated chronic disorders[34], [35], [36] and also elevated compared with PBC liver Th17 in PBC blood. In contrast to Th17 liver clonotypes, PSC and PBC MAIT17 liver clonotypes were decreased in PSC and PBC blood compared with HC and predicted Seq2MAIT frequencies of MAIT clonotypes were also decreased in PSC and PBC blood compared with HC. These findings align with previous studies reporting lower frequencies of MAIT cells in PSC blood^30^^,^^41^ as well as ALD, PBC, IBD, rheumatoid arthritis, and systemic lupus erythematosus,[41], [42], [43] suggesting that MAIT17 clonotypes may not be enriched in totality but could reflect increased IL-17 polarization of liver-resident MAIT cells or recruitment of circulating MAIT cells that acquire a tissue-resident MAIT17 phenotype in the PSC microenvironment.

Though we present the largest analysis of liver T cells in PSC by scRNA-seq and TCR-seq to date, including the greatest number of patients with PSC sampled from a considerable amount of liver explant material per patient (∼100 g), our study is still limited by a relatively small sample size and partial assessment of each liver explant per patient which may yield an incomplete capture of all T cells. Future studies in larger cohorts using material from multiple locations per explant with deeper sequencing as pathogenic antigen-specific T cells in PSC may be rare. For instance in celiac disease, only 0.1–1.2% of the total CD4^+^ T cells in intestinal biopsies of untreated patients with celiac disease were found to recognize gluten.^44^ Future studies including larger numbers of early-stage patients are needed to ascertain if Th17 and MAIT17 are also elevated throughout PSC progression or increased as a result of secondary effects resulting from chronic inflammation and cirrhosis. As our findings rely on single-cell methodologies, the spatial location of the different T cell subsets identified by scRNA-seq and potential interacting cell types are unknown but are key to unlocking relevant T cell phenotypes as Tregs in portal tracts but not lobular regions have already known to be differentially abundant in PSC and PBC livers.^8^ Future efforts implementing the latest subcellular spatial technologies in combination with deep cellular multi-marker phenotyping at the protein level by multiplex tissue staining will greatly improve the identification of disease-relevant T cell subsets within specific microanatomical locations and help delineate the key cell types mediating the production and presentation of lymphocyte antigens in PSC.

In conclusion, we show using multiple lines of evidence that Th17 and MAIT17 are enriched in livers of individuals with PSC which supports the potential use of targeted T cell therapies for the future treatment of PSC.

## Abbreviations

5-OP-RU, 5-(2-oxopropylideneamino)-6-d-ribitylaminouracil; 6-FP, 6-formylpterin; ALD, alcohol-related liver disease; ALP, alkaline phosphatase; ALT, alanine aminotransferase; D, dendritic cell; eGFP, enhanced green fluorescent protein; EM, effector memory; FIB-4, fibrosis-4; H, hepatocyte; HC, healthy controls; IBD, inflammatory bowel disease; IF, immunofluorescence; IL-17+ mucosal-associated invariant T cell; INR, international normalized ratio; J76-TPR, Jurkat 76 triple parameter reporter; LMC, liver mononuclear cells; MAIT, mucosal-associated invariant; MAIT17*, IL17*^+^ mucosal-associated invariant T cell; MASH, metabolic dysfunction-associated steatohepatitis; MELD-Na, model for end-stage liver disease; MFI, mean fluorescence intensity; MR1, major histocompatibility complex class I-related gene protein; P, plasma cell; PBC, primary biliary cholangitis; PSC, primary sclerosing cholangitis; scRNA-seq, single-cell RNA sequencing; snRNA-seq, single-nuclei RNA seq; T, T cell; T_c_, cytotoxic T cell; T_CM_, central memory T cell; TCR, T cell receptor; TCR-seq, T cell receptor sequencing; T_EM_, effector memory T cell; T_FH_-like, follicular-helper like T cell; Th17, T helper type 17; Treg, regulatory T cell; T_RM_, tissue-resident memory T cell; UMAP, uniform manifold approximation and projection.

## Authors’ contributions

Designed the study and wrote the manuscript: LRVB, EM, BKC. Performed wet lab experiments: LRVB, BKC. Assisted with bioinformatics and data management: JØ. Provided and reviewed clinical information: MSJ, AGB, DK, TF, YHO. Shared and analyzed blood TCR sequencing data: HE, AF. Assisted with flow cytometry setup and analysis: XJ. Supplied patient samples: YHO, PJT, THK, TF, EM. Coordinated project contributions: EM, BKC. All authors approved the final manuscript.

## Data availability

Data are available upon reasonable request from the corresponding author BKC.

## Financial support

This work was funded by the 10.13039/501100005416Research Council of Norway (325435), 10.13039/501100003725National Research Foundation of Korea (RS-2023-00268071) and Norwegian PSC Research Center. LRVB was supported by a Helse Sør-Øst PhD fellowship (340365) and MSJ by the Mildred-Scheel-Stipendienprogramm für Krebsforschung (57584079).

## Conflicts of interest

The authors declare no conflicts of interest.

Please refer to the accompanying ICMJE disclosure forms for further details.
