# Supplementary material for: Intrahepatic T cell analysis reveals Th17 and MAIT17 enrichment in primary sclerosing cholangitis
Source: JHEP Rep. 2026 Apr 17;8(9):101857. doi: 10.1016/j.jhepr.2026.101857 (PMC13507853; doi:10.1016/j.jhepr.2026.101857)
Supplement: Supplementary file 1 [file mmc1.pdf]

Intrahepatic T-cell analysis reveals Th17 and MAIT17 enrichment in primary sclerosing cholangitis

Lisa R. V. Brynjulfsen, Hesham ElAbd, Jonas Øgaard, Markus S. Jördens, Amber G. Bozward, Daniel Kearns, Xiaojun Jiang, Trine Folseraas, Palak J. Trivedi, Ye H. Oo, Andre Franke, Tom H. Karlsen, Espen Melum, Brian K. Chung

Table of contents

Supplementary materials & methods……………………………………………………….………2

Supplementary references…………………………………………………………………..8

Supplementary figures………………………………………………………………………9

Supplementary tables………………………………………………………………………18

Supplementary Materials & Methods

LMC isolation and cryopreservation

Liver sections (~100 g) were minced into 1-2 mm^3 pieces, washed with PBS and incubated at 37°C for 30 min in digestion medium consisting of RPMI-1640 supplemented with 3% fetal bovine serum (FBS) and collagenase type 1A (VWR International, Radnor, USA). Digested tissue was mechanically homogenized using a Stomacher 400 circulator (Seward, Worthing, UK) and filtered through 60-70 μm nylon strainers with PBS. Filtered suspensions were transferred into 50 ml conical tubes and spun at 600 g for 5 min. Supernatant was discarded and cell pellets were washed 3-4 more times in PBS. Washed pellets were resuspended in PBS and layered over Lympholyte-H (Cedarlane, Burlington, Canada) and spun at 600 g for 20 min to isolate LMCs by density centrifugation. Isolated LMCs were resuspended in Cryostor-10 (Sigma-Aldrich, St.Louis, USA) at 8-16 million cells per vial and stored in liquid nitrogen.[1]

T cell enrichment from LMCs

Cryopreserved LMCs were thawed and washed in warm RPMI 1640 with GlutaMAX and HEPES (Thermo Fisher Scientific, Waltham, USA) supplemented with 20% FBS (Sigma-Aldrich). Washed LMCs were resuspended in PBS (Lonza, Basel, Switzerland) supplemented with 2% FBS and 1 mM EDTA (Thermo Fisher Scientific), treated with 100 μg/ml DNase I (QIAGEN, Hilden, Germany) for 15 min and filtered through 30 μm strainers. T cells were enriched from LMCs using negative-selection magnetic bead kits following the manufacture's recommendations (STEMCELL Technologies, Vancouver, Canada). To exclude dead cells, enriched T cells were stained with 7-aminoactinomycin D (7-AAD, 1:333 dilution; Thermo Fisher Scientific) or Zombie NIR (1:333 dilution; BioLegend, San Diego, USA) for 5 min and sorted using a FACSMelody flow cytometer (BD Biosciences, Franklin Lakes, USA). Viable T cell enrichments were filtered through 30 μm strainers and enumerated using a Countess II Automated Cell Counter (Thermo Fisher Scientific) for scRNA-seq.

Preprocessing, clustering, differential gene expression analysis and cluster annotation

Sequencing reads were demultiplexed and aligned to human genome GRCh38-2020-A using the Cell Ranger v7.1.0 multi pipeline (10x Genomics).[2] Filtered outputs were loaded into R v4.3.2 for preprocessing with Seurat v.5.0.1.[3] Features detected in fewer than 3 cells and cells with fewer than 200 detected features were excluded. Cell doublets and empty droplets outside the 1.5 interquartile range (IQR) for total unique molecular identifier (UMI) counts per cell and unique features per cell were excluded. Further, dead cells with mitochondrial gene count percentage per cell above the upper fence of the 1.5 IQR were also excluded. Preprocessed samples were merged into a single SeuratObject and normalized using NormalizeData. The top 2000 variable features were identified using FindVariableFeatures and scaled using ScaleData. Principal component analysis (PCA) was performed using RunPCA and batch effects were adjusted using CCA integration.[4, 5]

To define cell clusters, FindNeighbors was performed using the first 30 principal components (PCs) followed by FindClusters with a resolution parameter of 1.3. DEG analysis for each cluster was performed using FindAllMarkers to obtain marker genes and all reported cluster marker genes reached statistical significance ($P<0.01$, Wilcoxon Rank Sum test) and ordered by ascending P-value. To exclude non-T cells, only cells with normalized counts of *CD3D* > 0,

CD68 < 1, *CD79A* < 2, *CD79B* < 2, and *ALB* < 5 were analyzed. Reclustering of filtered T cells was repeated using the first 21 PCs and resolution setting of 0.5. Marker DEGs for each T cell cluster were identified using FindAllMarkers and reached a $\log_2$ fold-change > 0.5, expressed in > 25% of the cells in the cluster and had an adjusted P-value < 0.05. Heatmaps showing the top 40 DEGs for each cluster in the total T cell UMAP and the top 25 DEGs for the Th17/MAIT17 were generated using DoHeatmap and downsampled to display a maximum of 1000 cells per cluster for the total T cell UMAP.

Using canonical lineage markers,[6-10] all 13 T cell clusters in the total T cell UMAP were annotated into discrete phenotypes. Cluster 1 ($CD4^+$ T_{EM}1) was the most abundant in 14 of 24 livers (18.5 ± 8.0%) and expressed the effector memory (EM) markers *TNF*, *IFNG* and *CXCR3*. Of the four remaining $CD4^+$ subsets, cluster 3 ($CD4^+$ T_{CM}) was the second largest and featured the central memory (CM) markers *CCR7* and *IL7R*, while cluster 6 ($CD4^+$ T_{EM}2) shared EM markers with $CD4^+$ T_{EM}1 (*CD40LG*, *IL7R*, *CD4*) but elevated expression of *KLRB1*, *STAT1*, and *CXCR6*. Cluster 11 (Tregs) featured the conventional Treg genes *FOXP3*, *CTLA4*, *CD27*, *IL2RA* and cluster 12 expressed the follicular-helper T cell (T_{FH}) markers *CTLA-4*, *PDCD1*, *TOX2*, and *ICOS*.

Of the five $CD8^+$ subsets, cluster 2 (innate-like $CD8^+$ T_c) was the most abundant (14.2 ± 14.2%) and featured co-expression of the innate leukocyte marker *FCGR3A* and the cytotoxic T cell (T_c) genes *FGFBP2*, *GZMB* and *PRF1*. Cluster 4 ($CD8^+$ T_{RM}) was classified as $CD8^+$ tissue-resident memory (RM) based on *ITGAE* and *CXCR6* expression and cluster 7 (innate-like γδ T cells) featured both *FCGR3A* and the TCR γδ-genes *TRDC* and *TRDV1*. Cluster 9 ($CD8^+$ T_c) expressed the cytotoxic markers *GZMK*, *GZMA*, *NKG7* but lacked innate leukocyte markers. Cluster 10 ($CD8^+$ T_{EM}/γδ) expressed low levels of *CCR7* and cytotoxic markers characteristic of $CD8^+$ effector memory T cells and was also positive for the TCR γδ-genes *TRDC* and *TRDV1*.

Three of 13 subsets contained a mixture of both $CD4^+$ and $CD8^+$ cells with cluster 5 constituting the largest (8.7 ± 5.5%) featuring *CCR6*, *RORC*, *IL23R* and *KLRB1* indicative of both Th17 and MAIT17 cells. Cluster 8 was annotated as naïve based on the expression of *CCR7*, *SELL*, and *TCF7* while cluster 13 (cycling) was the smallest T cell subset overall (0.6 ± 0.4%) and distinguished by heightened expression of the cell cycle markers *MKI67* and *RRM2*.

To partition the Th17/MAIT17 cluster, Th17/MAIT17 cells was subsetted from total T cells and reclustered using the same methods described for total T cells with PCA set on *TRAV1-2*, *CD8A*, *CD8B*, *NKG7*, *NCR3*, *GZMK*, *CTSW*, *CD4*, *GPR183*, *MAP3K4*, *CD40LG*, *SAMSN1* and *AHR*.

Data integration was performed using CCA integration with the k.weight parameter set to 41. The first 10 PCs were used for clustering Th17/MAIT17 cells. Resolution was set to 0.2. UMAPs were generated with RunUMAP using the first 10 PCs and minimum distance of 0.8.

Clusters annotated as CD4-positive, alpha-beta T cell, CD8-positive, alpha-beta T cell and T cell from Andrews and Nakib *et al*. (https://cellxgene.cziscience.com/collections/0c8a364b-97b5-4cc8-a593-23c38c6f0ac5) were merged into a single SeuratObject with total T cells from our study. NormalizeData, FindVariableFeatures, ScaleData, RunPCA, CCA integration with

k.weight set to 42. Clustering and generation of UMAP on merged data was performed using the first 25 PCs and with the resolution parameter set to 0.6.

Analysis of liver and blood TCR overlap and blood MAIT cell frequencies
DNA was extracted from EDTA-blood of individuals with PSC (n=504), PBC (n=63) and age and sex-matched controls (n=904) using the Qiagen DNeasy Blood Extraction Kit (QIAGEN, Hilden, Germany). Isolated DNA was amplified for TCR β-chain repertoires using the immuneSEQ assay (Adaptive Biotechnologies, Seattle, USA) as previously reported.[11] Briefly, after library preparation, sequencing and clonotype identification, non-productive clonotypes containing a stop-codon or a frameshift were excluded. Different V(D)J recombination sequences encoding the same TCR β-chain sequence at the protein level were combined by summing their expansion thus a blood TCR clonotype was defined herein as a unique recombination of V and J genes and a unique amino acid CDR3 sequence.

TCR and MR1 DNA constructs and lentiviral vectors
Paired TCR αβ-chain variable domain RNA sequences from a subset of expanded PSC liver MAIT17 clonotypes identified by TCR-seq (n=10, Supplementary Table 3) were cloned into DNA constructs for lentiviral transduction into J76-triple parameter reporters (J76-TPR) that lack endogenous TCR and express enhanced green fluorescent protein (eGFP) upon TCR-mediated NFAT activation.[12, 13] For each TCR construct, a MAIT17 TCR β-chain variable domain sequence from TCR-seq was followed by sequences for a murine TCR β-chain constant region, cysteine bridge, P2A self-cleaving peptide, MAIT17 TCR α-chain variable domain from TCR-seq, murine TCR α-chain constant region and cysteine bridge.[14] To generate the MAIT17 TCR negative control, protein sequences for the TCR αβ-variable domains know to recognize a HLA-DRB1*13:01-restricted p53 mutant (4285-TCR1)[15] were reverse translated and codon-optimized for expression in human cells. Human MR1 sequence (GenBank: NM_001531.2) was cloned into DNA constructs for lentiviral transduction of H69 cholangiocytes.[16] TCR- and MR1-encoding pLV-Puro-EF1α lentiviral vectors were synthesized by VectorBuilder (Chicago, Illinois, USA) and detailed in Supplementary Figure 1A and 1B.

Flow cytometry
Cryopreserved LMCs were thawed in RPMI 1640 with GlutaMAX and HEPES (Thermo Fisher Scientific) containing 20% FBS (Sigma-Aldrich), filtered through 30 μm strainers and incubated at 10^6 cells/ml in RPMI containing 10% FBS for 3.5 hr at 37°C in a humidified 5% CO_2 incubator. Following incubation, cells were treated with FcR blocking reagent (1:5, Miltenyi Biotec, Bergisch Gladbach, Germany) for 10 min at 4°C in PBS (Lonza), washed and then stained with BV421 anti-human CCR6 (1:100, clone G034E3, BioLegend) and BV786 anti-human CCR7 (1:50, clone 3D12, Thermo Fisher Scientific) for 30 min at 37°C in Brilliant stain buffer (1:2, Thermo Fisher Scientific) diluted with RPMI and 10% FBS. Cells were then stained for 30 min at 4°C for extracellular markers using the following antibodies diluted 1:50 in a mixture of PBS, Brilliant stain buffer (1:2, Thermo Fisher Scientific) and CellBlox Plus blocking buffer (1:20, Thermo Fisher Scientific): Alexa Fluor 488 anti-human CD3 (clone OKT3, Thermo Fisher Scientific), PE/Fire 810 anti-human TCR γ/δ (clone B1, BioLegend), RB780 anti-human CD161 (clone DX12, BD Biosciences), BV480 anti-human TCR Vα7.2 (clone OF-5A12, BD Biosciences), PerCP/Cyanine5.5 anti-human CD8a (clone RPA-T8, BioLegend), BUV 496 anti-human CD4 (clone RPA-T4, Thermo Fisher Scientific), BUV395 anti-human CD45RA (clone

HI100, Thermo Fisher Scientific), NovaFluor Red 700 anti-human CD25 (clone BC96, Thermo Fisher Scientific), BV650 anti-human PD-1 (clone J105, Thermo Fisher Scientific), Super Bright 600 anti-human ICOS (clone ISA-3, Thermo Fisher Scientific) and LIVE/DEAD Fixable Near-IR Dead Cell Stain (1:200, Thermo Fisher Scientific). LMCs were washed, fixed, permeabilized and re-stained for intracellular markers using the Foxp3 intracellular staining kit (Thermo Fisher Scientific) following the manufacturer´s instructions. Intracellular staining was performed with PE-eFluor 610 anti-human RORγt (1:200, clone AFKJS-9, Thermo Fisher Scientific), PE-Cy7 anti-human FOXP3 (1:50, clone PCH101, Thermo Fisher Scientific), BUV737 anti-human CTLA-4 (1:50, clone 14D3, Thermo Fisher Scientific), PE anti-human FGFBP2 (1:50, clone TDA3, BioLegend), and BV711 anti-human Bcl-6 (1:50, clone K112-91, BD Biosciences) in Brilliant stain buffer (1:2 diluted in 1x permeabilization buffer) for 30 min at room temperature. LMCs were washed in permeabilization buffer, resuspended in PBS and filtered using 30 μm strainers prior for data acquisition on a ID7000 Spectral Cell Analyzer (Sony Biotechnology, San Jose, USA) equipped with five lasers (355 nm, 405 nm, 488 nm, 561 nm, 637 nm). Data was unmixed using Sony ID7000 software v2.0.2.17121 and analyzed with FlowJo v10.10.0 (BD Life Sciences, Franklin Lakes, New Jersey, USA). Fluorescence minus one (FMO) controls were used to determine positive stainings and gating for MAIT17 and Th17 cells is shown in Supplementary Figure 7.

To determine the reactivity of TCR-transduced J76-TPR cell lines to 5-OP-RU, approximately 100,000 cells were stained with PE-conjugated human MR1:5-OP-RU tetramer (1:400) or MR1:6-formylpterin (6-FP, 1:400) control tetramer from the NIH Tetramer Core (Emory University, Atlanta, USA) for 30 min at 4°C. Cells were washed and stained with anti-mouse TCR β-chain-APC (1:50, clone H57-597, BioLegend). Dead cells were excluded using Zombie Violet (1:200, BioLegend). MR1 expression on H69-MR1 cells was measured using anti-human MR1-APC (1:50, clone 26.5, BioLegend). TCR reporters and H69-MR1s were analyzed by flow cytometry data using a LSRFortessa flow cytometer (BD Biosciences) and FlowJo v10.10.0 (BD Life Sciences). All stainings were performed on ice in PBS (Lonza). Gating strategy for MR1 expression on H69-MR1 cells in shown in Supplementary Figure 3A and 3B, while gating strategy for tetramer staining is detailed in Supplementary Figure 2A.

5-OP-RU induced activation of MAIT17 reporter cell lines

On day 1, H69-MR1 cells were seeded at 1 x 10^4 per well in a 96-well flat-bottom plate and incubated overnight in a 37°C humidified 5% CO_2 incubator. On day 2, MAIT17 TCR-transduced J76-TPR cell lines (TCR-MAIT) were added at 5 x 10^4 per well in H69 media supplemented with the indicated concentrations of 5-OP-RU and incubated for 48 hrs in a 37°C humidified 5% CO_2 incubator. J76-TPR cells expressing a non-MAIT17 TCR (4285-TCR1) were cultured with 5-OP-RU-loaded H69-MR1 cells as a negative control. Parental J76-TPR cells cultured with 1000 nM 5-OP-RU without H69 cells also served as negative controls. 5-OP-RU was prepared as by incubating 5-amino-6-D-ribitylaminouracil (5-A-RU, MuseChem, Paoli, Pennsylvania, USA) with methylglyoxal (Sigma-Aldrich) at a 1:25 ratio for 15 minutes at room temperature as previously described.[17] NFAT-eGFP expression used to detect 5-OP-RU activation by MAIT17-TCR reporters was measured using LSRFortessa flow cytometer (BD Biosciences) and analyzed in FlowJo v10.10.0 (BD Life Sciences). Gating strategy for identifying activated TCR-transduced reporter cell lines is shown in Supplementary Figure 2B.

Lentiviral transduction and culture of J76-TPR and H69 cell lines
J76-TPR cells cultured in RPMI 1640 with GlutaMAX and HEPES (Thermo Fisher Scientific) supplemented with 10% fetal bovine serum (FBS; Sigma-Aldrich) and penicillin-streptomycin (1:100, Thermo Fisher Scientific) were infected with TCR-encoding pLV-Puro-EF1α lentiviral vectors at a multiplicity of infection (MOI) of 5 using 8.0 μg/ml polybrene (VectorBuilder). Cells and vectors were spun at 800 g for 30 min at room temperature and maintained in a 37°C humidified 5% CO_2 incubator. Three days post-infection, TCR-transduced J76-TPR cells were selected using 2.5 μg/ml puromycin (Applied Biological Materials, Richmond, Canada) for 48 hr. To generate MR1-transduced H69 cells (H69-MR1), H69 cholangiocytes cultured to 50% confluency in H69-specific supplemented medium [16] were infected overnight with MR1-encoding pLV-Puro-EF1α lentiviral vectors at an MOI of 25 with 8.0 μg/ml polybrene (VectorBuilder) in a 37°C humidified 5% CO_2 incubator. Three days post-infection, H69-MR1s were selected with 3 μg/ml puromycin (Applied Biological Materials) for 48 hr. Following selection, all transduced cell lines were cultured in non-selective media and maintained in a 37°C humidified 5% CO_2 incubator for experiments.

Immunohistochemistry (IHC), immunofluorescence (IF) and staining quantification
Formalin-fixed paraffin-embedded 5 μm liver sections were deparaffinized in histoclear (Histolab Products AB, Askim, Sweden) for 10, 8 and 5 min and rehydrated in series of decreasing ethanol solutions (100% 2x for 1 min, 96% for 1 min, 80% for 1 min and 50% for 1 min) and then stored in distilled water. Antigen retrieval was performed in Envision FLEX Target Retrieval pH 6.1 solution (Agilent Technologies, Santa Clara, USA) for 20 min at 95°C. Sections were left cooling in the machine for 15 min, transferred to the bench top for another 15 min and then placed in PBS. Sections used for RORγt IHC detection and IF staining underwent nuclear permeabilization with 0.1% Triton X-100 (Sigma-Aldrich) in PBS (Santa Cruz Biotechnology, Dallas, USA) for 10 min followed by three washes with 0.1% Tween-20 in PBS (PBST). RORγt and IF sections were the treated with 3% hydrogen peroxide (Merck KGaA, Darmstadt, Germany) while sections used for CD3 IHC staining were treated with 0.3% hydrogen peroxide in PBS for 20 min. All sections were then washed three times with PBST and then blocked with 2.5% normal goat serum or 2.5% normal horse serum (Vector Laboratories, Newark, USA) for 20 min. Sections were incubated overnight at 4°C with mouse anti-human RORγt (1:250, clone 6F3.1, Sigma-Aldrich) or rabbit anti-CD3 (1:500, clone SP162, Abcam, Cambridge, UK) in PBST containing 0.1% bovine serum album (Sigma-Aldrich). Mouse IgG2a antibody (clone: MOPC-173, BioLegend) and rabbit IgG (clone: EPR25A, Abcam) were used as isotype controls.

After overnight incubation, sections for IHC washed 3 times in PBST and stained with ImmPRESS HRP-conjugated goat anti-mouse IgG for 30 min or ImmPRESS HRP-conjugated horse anti-rabbit IgG (Vector Laboratories) for 20 min. After three final washes in PBST, sections used for IHC RORγt detection were stained with DAB (Vector Laboratories) for 3 min while sections used for CD3 detection were stained with DAB for 4 min. Sections were then stored in water for 5 min, counterstained with hematoxylin (Vector Laboratories), dehydrated in series of increasing ethanol solutions (50% for 1 min, 80% for 1 min, 96% for 1 min and 100% for 1 min), washed 3x in histoclear for 1 min and mounted using Eukitt Quick-hardening mounting medium (Sigma-Aldrich). All steps were performed at room temperature unless stated

otherwise. Annotation of positive and negative cells was performed in QuPath v0.5.[18] using the train object classifier set to random trees. While viewing the image on live update, the classifier was trained by manually annotating 10-20 positive and negative cells for CD3 and 20-40 positive and negative cells for RORγt. After automated classification, CD3 and RORγt positive staining was evaluated and adjusted. Percentage of $CD3^+$ cells was calculated by dividing the number of $CD3^+$ cells on the total number of cells detected within the tissue analyzed for CD3. The percentage of RORγt^+ T cells was calculated by dividing the number of RORγt^+ cells on the number of $CD3^+$ cells quantified within the same tissue area.

For IF staining, sections incubated overnight with primary antibodies were washed 3 times in PBST and stained for 30 min with Alexa Fluor 555 donkey anti-mouse IgG (1:1000, Thermo Fisher Scientific) and Alexa Fluor 647 goat anti-rabbit IgG (1:1000, Thermo Fisher Scientific) in PBST containing 0.1% bovine serum albumin. After washing three times with PBST, IF sections were mounted using EverBrite TrueBlack Hardset Mounting Medium with DAPI (Biotium, Fremont, USA). All steps were performed at room temperature unless stated otherwise. Slides were imaged using a Nikon Eclipse E400 Microscope equipped with a Nikon camera DS-Fi3 (Minato, Japan) and merged using Fiji ImageJ v2.16.0/1.54p[19]

Supplementary References

[1] Chung BK, Guevel BT, Reynolds GM, et al. Phenotyping and auto-antibody production by liver-infiltrating B cells in primary sclerosing cholangitis and primary biliary cholangitis. J Autoimmun. 2017;77:45-54.

[2] Zheng GX, Terry JM, Belgrader P, et al. Massively parallel digital transcriptional profiling of single cells. Nat Commun. 2017;8:14049.

[3] Hao Y, Stuart T, Kowalski MH, et al. Dictionary learning for integrative, multimodal and scalable single-cell analysis. Nat Biotechnol. 2024;42:293-304.

[4] **Stuart T, Butler A,** Hoffman P, et al. Comprehensive Integration of Single-Cell Data. Cell. 2019;177:1888-1902 e1821.

[5] Butler A, Hoffman P, Smibert P, et al. Integrating single-cell transcriptomic data across different conditions, technologies, and species. Nat Biotechnol. 2018;36:411-420.

[6] **Poch T, Krause J,** Casar C, et al. Single-cell atlas of hepatic T cells reveals expansion of liver-resident naive-like CD4(+) T cells in primary sclerosing cholangitis. J Hepatol. 2021;75:414-423.

[7] Xu W, Zhao X, Wang X, et al. The Transcription Factor Tox2 Drives T Follicular Helper Cell Development via Regulating Chromatin Accessibility. Immunity. 2019;51:826-839 e825.

[8] Andreatta M, Corria-Osorio J, Muller S, et al. Interpretation of T cell states from single-cell transcriptomics data using reference atlases. Nat Commun. 2021;12:2965.

[9] Ramachandran P, Dobie R, Wilson-Kanamori JR, et al. Resolving the fibrotic niche of human liver cirrhosis at single-cell level. Nature. 2019;575:512-518.

[10] **Alvisi G, Termanini A,** Soldani C, et al. Multimodal single-cell profiling of intrahepatic cholangiocarcinoma defines hyperactivated Tregs as a potential therapeutic target. J Hepatol. 2022;77:1359-1372.

[11] ElAbd H, Pesesky M, Innocenti G, et al. T and B cell responses against Epstein-Barr virus in primary sclerosing cholangitis. Nat Med. 2025.

[12] Muller TR, Schuler C, Hammel M, et al. A T-cell reporter platform for high-throughput and reliable investigation of TCR function and biology. Clin Transl Immunology. 2020;9:e1216.

[13] Jutz S, Leitner J, Schmetterer K, et al. Assessment of costimulation and coinhibition in a triple parameter T cell reporter line: Simultaneous measurement of NF-kappaB, NFAT and AP-1. J Immunol Methods. 2016;430:10-20.

[14] Muller TR, Jarosch S, Hammel M, et al. Targeted T cell receptor gene editing provides predictable T cell product function for immunotherapy. Cell Rep Med. 2021;2:100374.

[15] Malekzadeh P, Pasetto A, Robbins PF, et al. Neoantigen screening identifies broad TP53 mutant immunogenicity in patients with epithelial cancers. J Clin Invest. 2019;129:1109-1114.

[16] Grubman SA, Perrone RD, Lee DW, et al. Regulation of intracellular pH by immortalized human intrahepatic biliary epithelial cell lines. Am J Physiol. 1994;266:G1060-1070.

[17] **Valestrand L, Zheng F,** Hansen SH, et al. Bile from Patients with Primary Sclerosing Cholangitis Contains Mucosal-Associated Invariant T-Cell Antigens. Am J Pathol. 2022;192:629-641.

[18] Bankhead P, Loughrey MB, Fernandez JA, et al. QuPath: Open source software for digital pathology image analysis. Sci Rep. 2017;7:16878.

[19] Schindelin J, Arganda-Carreras I, Frise E, et al. Fiji: an open-source platform for biological-image analysis. Nat Methods. 2012;9:676-682.

[20] **Andrews TS, Nakib D,** Perciani CT, et al. Single-cell, single-nucleus, and spatial transcriptomics characterization of the immunological landscape in the healthy and PSC human liver. J Hepatol. 2024;80:730-743.

Supplementary Figures

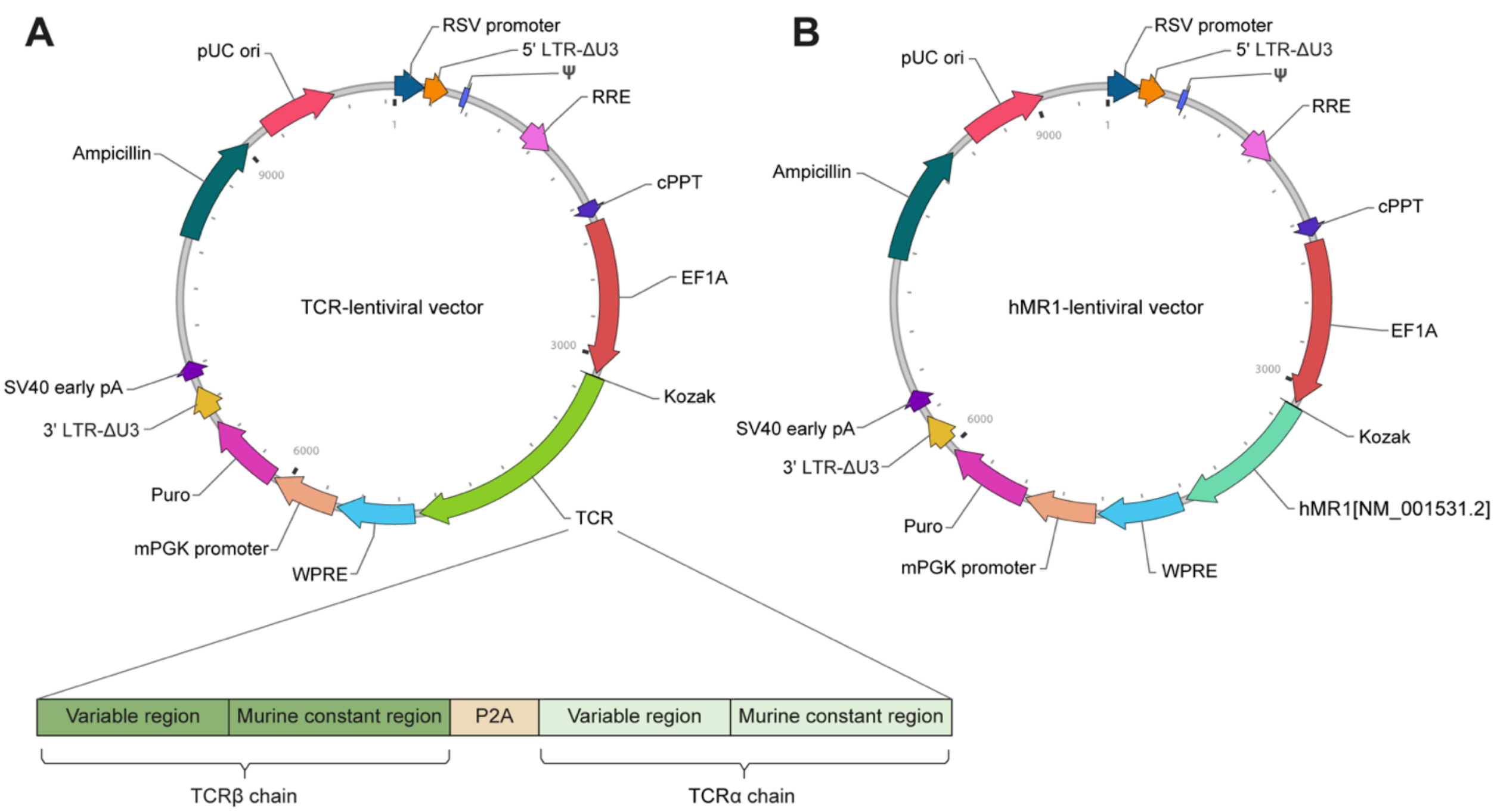

Supplementary Figure 1. Detailed overview of lentiviral TCR and MR1 expression vectors used to generate MAIT17 TCR reporters and H69-MR1 cell line. (A) Representative lentiviral vector used to transduce TCR expression into J76-TPR cell lines. (B) Lentiviral vector used to drive human MR1 overexpression in H69 cholangiocytes. hMR1, human major histocompatibility complex class I-related gene protein; P2A, peptide 2A sequence.

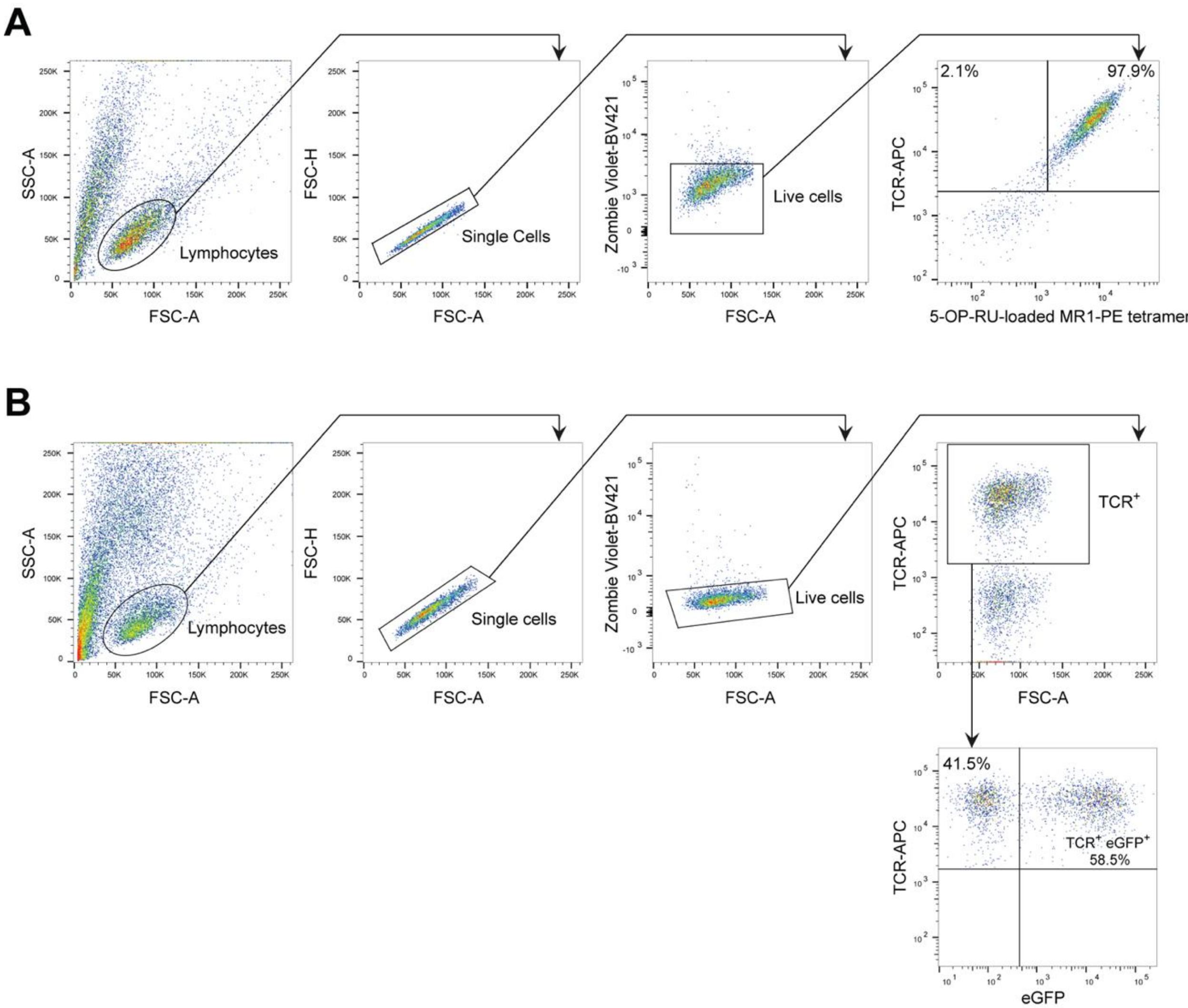

Supplementary Figure 2. Gating strategy for 5-OP-RU-MR1 tetramer staining and activation of TCR reporter cell lines by flow cytometry. (A) TCR-transduced cell lines were gated for lymphocyte morphology and singlets using forward (FSC) and side scatter (SSC). Lymphocyte singlets were gated for viability (Zombie Violet$^-$) and TCR and MR1-tetramer staining. TCR$^+$MR1-tetramer$^+$ cells were categorized as prototypical MAIT cells. Gated frequencies show the percentage of MR1-tetramer$^+$ cells as a fraction of total TCR$^+$ cells. (B) TCR-transduced reporter lines were analyzed for TCR activation by gating on lymphocyte morphology, singlets, live cells (Zombie Violet$^-$) and TCR expression. Gated frequencies show the percentage of TCR$^+$eGFP$^+$ cells as a fraction of total TCR$^+$ cells.

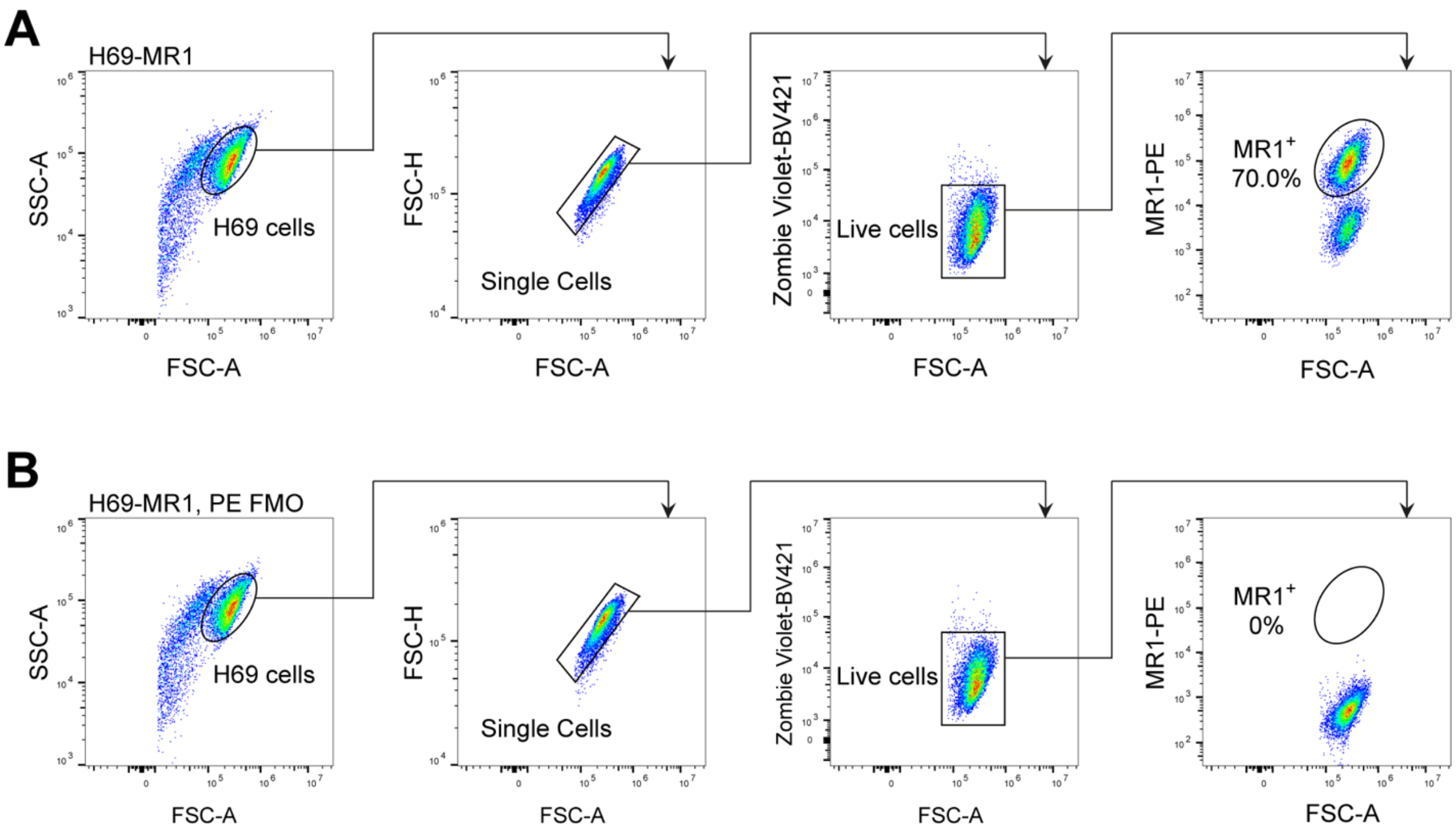

Supplementary Figure 3. MR1 expression on H69-MR1 cholangiocyte cells by flow cytometry. (A) MR1-H69 cells were analyzed for MR1 expression by gating for H69 morphology, singlets using forward (FSC) and side scatter (SSC), live cells (Zombie Violet^{-}), and MR1 staining. (B) FMO control for MR1 staining to assist with the gating of MR1^{+} cells. H69-MR1 cells were gated on H69 morphology, singlets, live cells (Zombie Violet^{-}), and MR1 staining. FMO, fluorescence minus one; MR1, major histocompatibility complex class I-related gene protein.

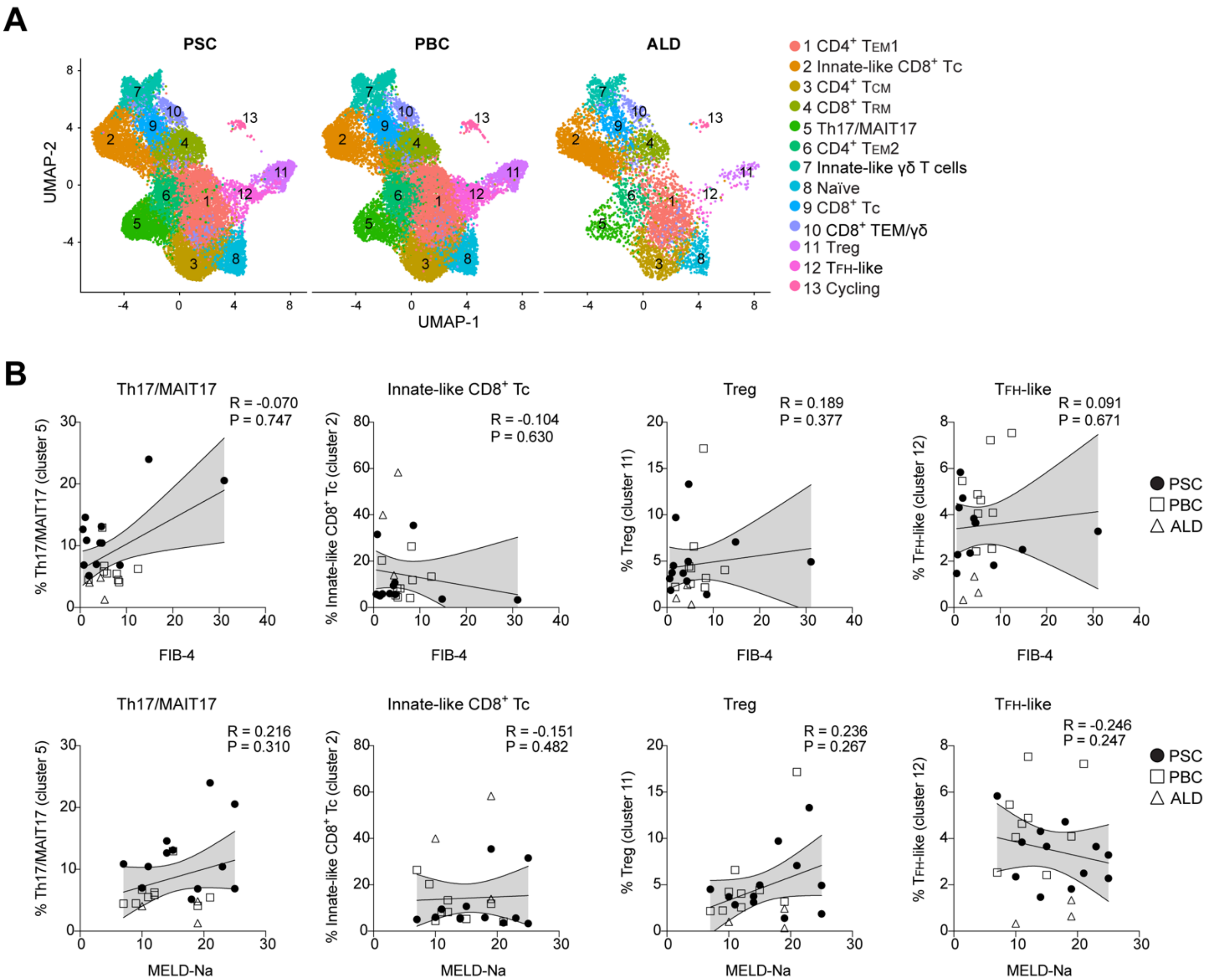

Supplementary Figure 4. Total T cell scRNA-seq UMAP split by disease type and correlation of FIB-4 and MELD-Na with T cell proportions. (A) UMAP showing PSC (n=12, 16,641 cells), PBC (n=9, 18,426 cells), and ALD livers (n=3, 5,830 cells) analyzed by scRNA-seq. (B) Correlation analysis of FIB-4 and MELD-Na with proportions of liver T cell subsets differentially-abundant in PSC, PBC and ALD measured by scRNA-seq (i.e. Th17/MAIT17, innate-like CD8^{+} cytotoxic T_c, Treg, and T_{FH}-like). Statistical significance for correlation analyses evaluated using Spearman.

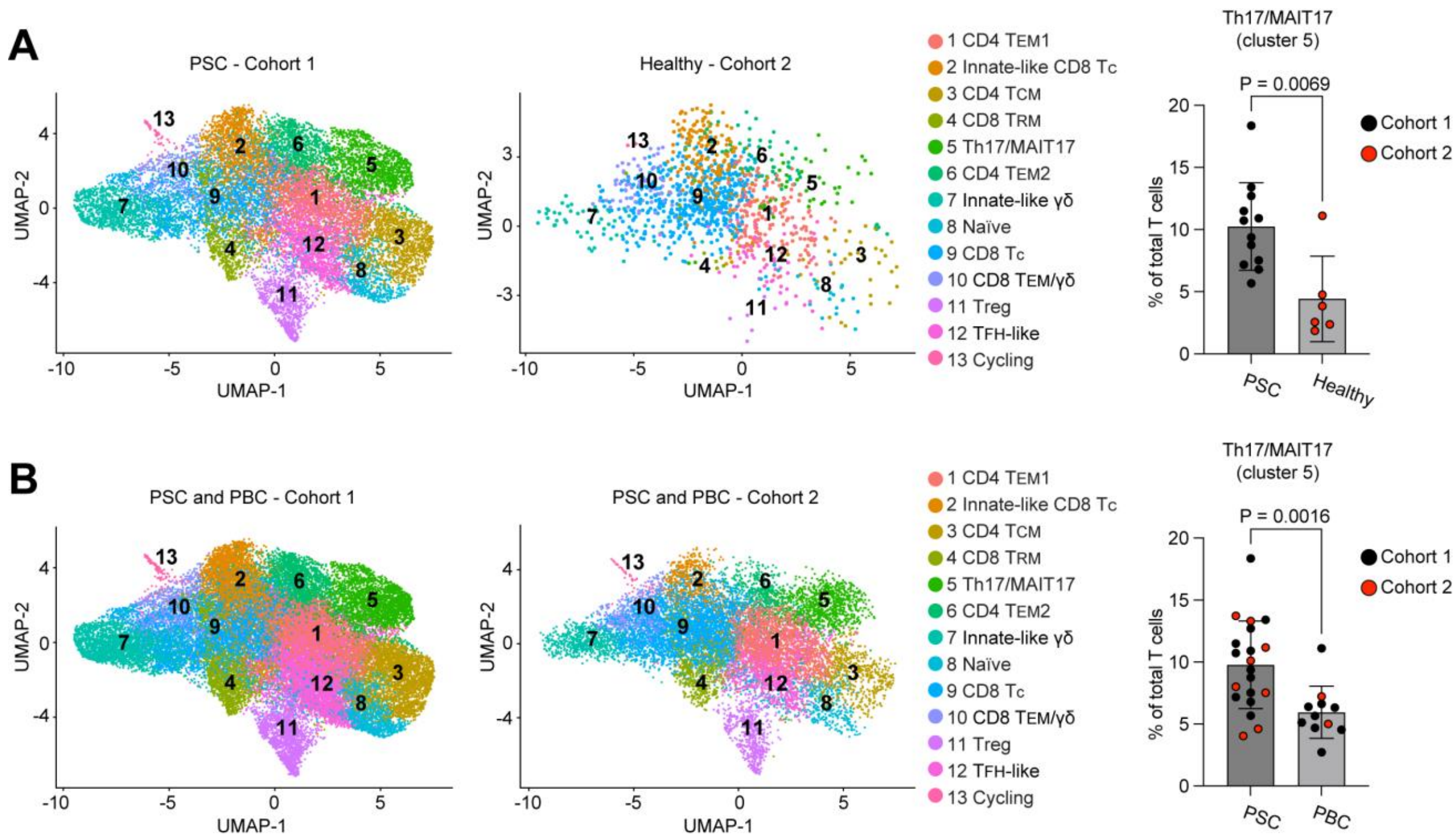

Supplementary Figure 5. PSC livers have increased Th17/MAIT17 proportions compared to healthy and PBC controls. (A) UMAPs indicate clustering of T cells from 12 PSC livers (Supplementary Table 1) herein and 6 healthy controls from Andrews and Nakib *et al*.[20] assessed by scRNA-seq (Cohort 1). Bar graph shows Th17/MAIT17 proportion as percentage of total T cells. (B) UMAPs indicate clustering of PSC and PBC liver T cells (12 PSC, 9 PBC; Supplementary Table 1) and Andrews and Nakib *et al*.[20] (8 PSC, 2 PBC) assessed by scRNA-seq (Cohort 2). Bar graph shows Th17/MAIT17 proportion as percentage of total T cells. Statistical significance evaluated using Mann-Whitney.

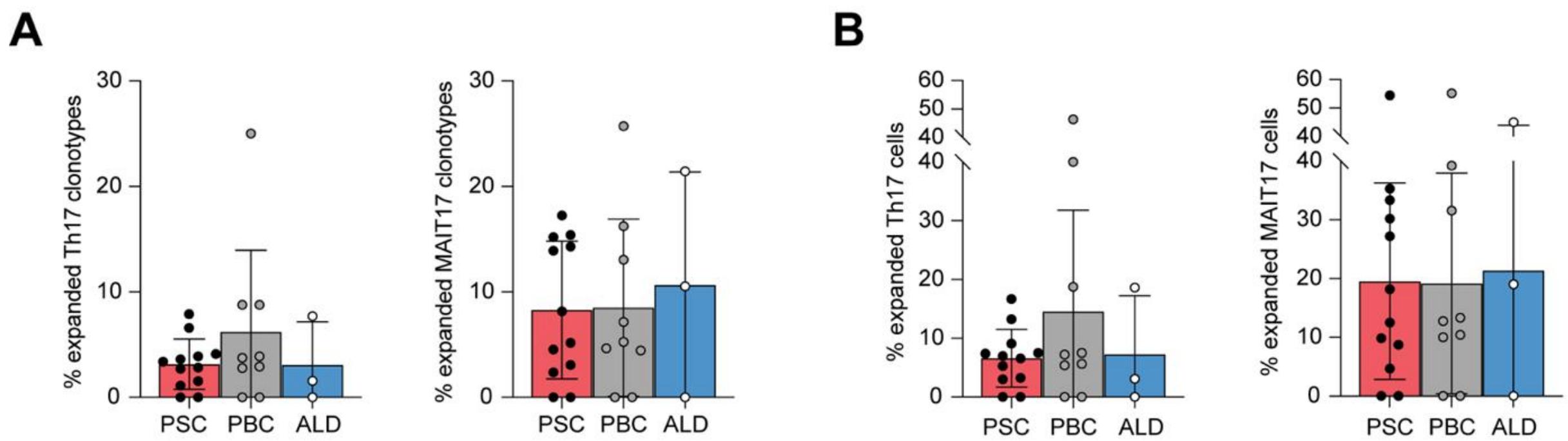

Supplementary Figure 6. Clonal expansion of Th17 and MAIT17 enrichment in PSC. (A) Percentage of expanded Th17 and MAIT17 clonotypes. (B) Percentage of expanded Th17 and MAIT17 cells.

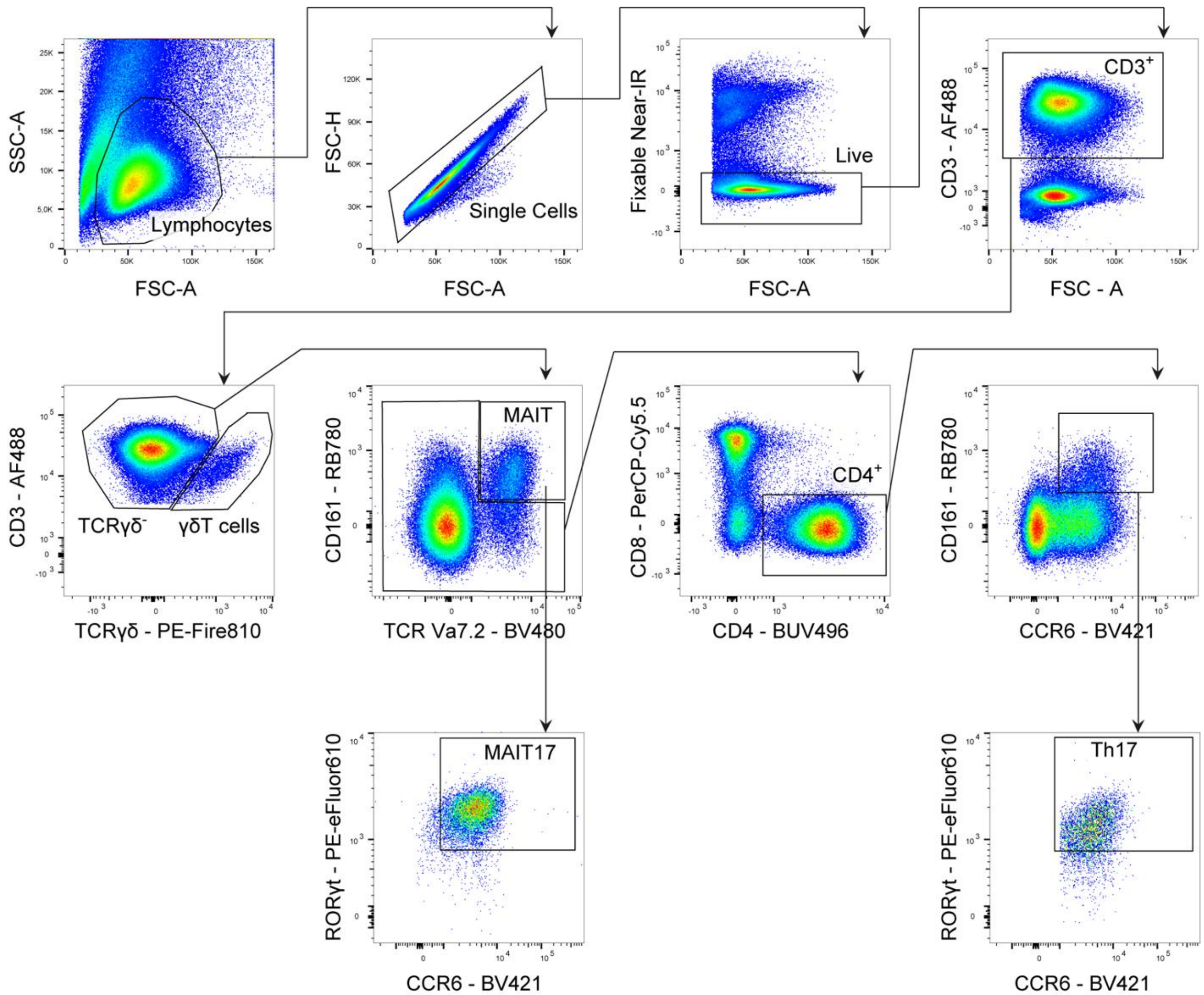

Supplementary Figure 7. Gating strategy for the identification of liver MAIT17 and Th17 cells by flow cytometry. Cryopreserved LMCs stained for MAIT17 and Th17 markers were first gated on lymphocyte morphology and single cells using forward (FSC) and side scatter (SSC). Dead cells were excluded using Fixable Near-IR and TCRγδ$^+$ cells were excluded from live CD3E$^+$TCRαβ$^+$ cells. MAIT cells within live CD3E$^+$TCRαβ$^+$ cells were identified as CD161$^+$TCRVα7.2$^+$ and defined as MAIT17 by RORγt$^+$ and CCR6$^+$ co-staining. TCRVα7.2$^-$ cells positive for CD4, CD161 and RORγt were enumerated as Th17 cells.

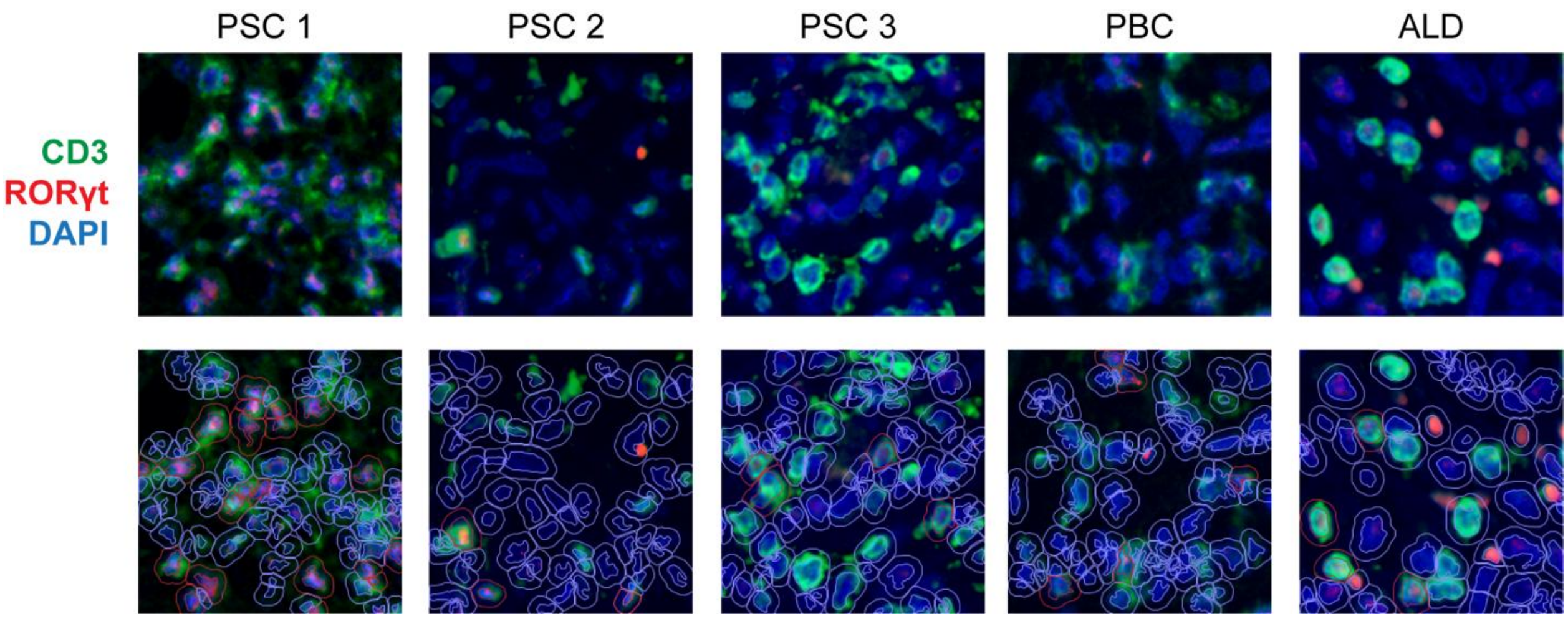

Supplementary Figure 8. Immunofluorescence and quantification of Th17/MAIT17 cells in liver tissue from PSC and disease controls. Images in top row indicate $CD3^+$ (green), nuclear $ROR\gamma t^+$ (red) and $DAPI^+$ nuclei (blue) IF staining of liver tissue from 3 PSC, 2 PBC and 1 ALD explant. For each liver section, numbers of single positive ($ROR\gamma t^+$) and double-positive ($CD3^+ROR\gamma t^+$) cells were quantified for 3 regions (150 μm^2). Quantification was performed in QuPath v0.5[18] using the train object classifier set to random trees. To train the classifier, 5-15 negative, single positive or double-positive cells were manually annotated. Within each region, T cell percentages of total $ROR\gamma t^+$ were calculated by dividing the number of double-positive on the number of single-positive. Mean T cell percentages of total $ROR\gamma t^+$ cells for 3 regions was then average for each sample.

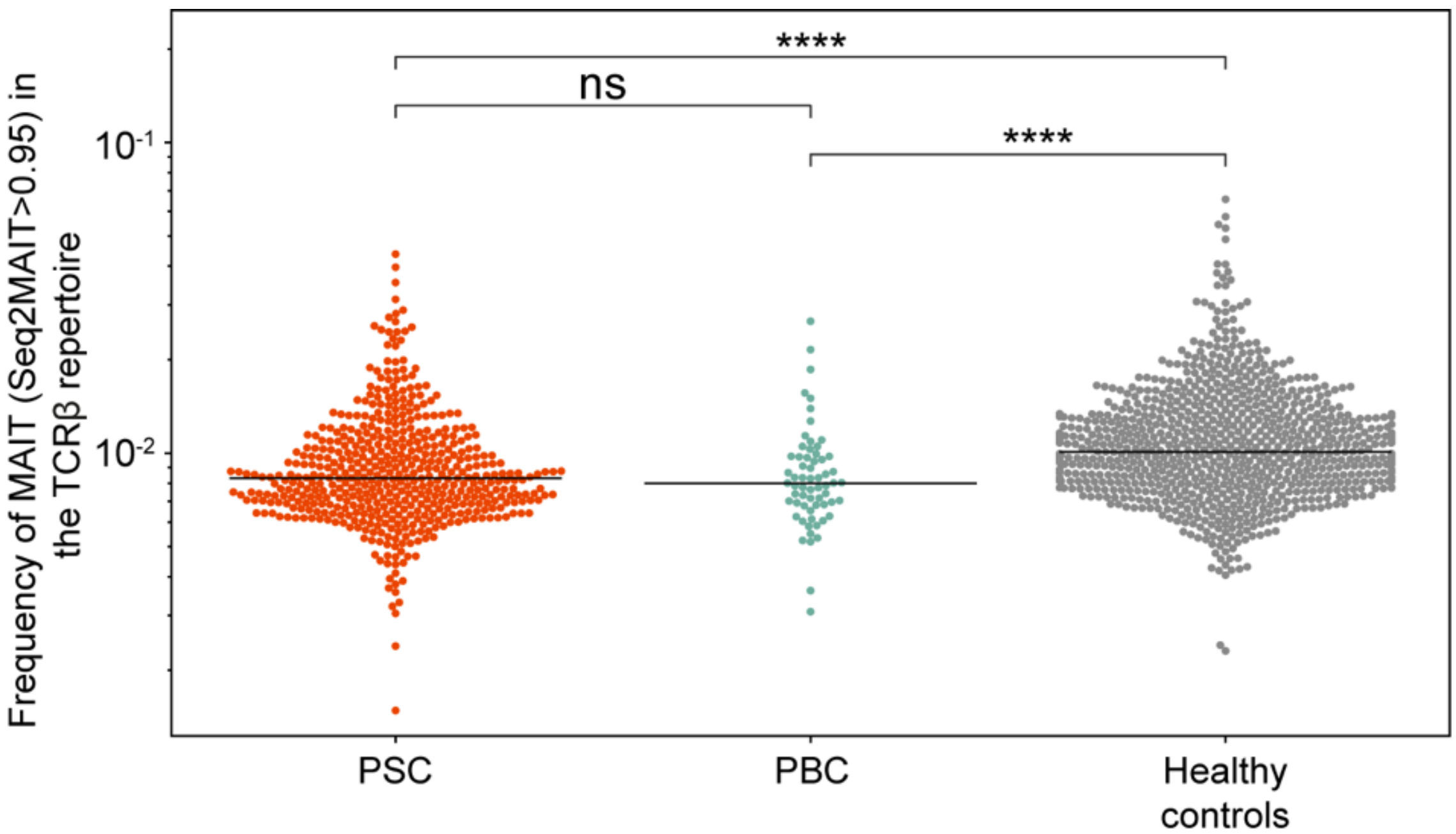

Supplementary Figure 9. Predicted MAIT cell clonotype frequencies in blood PSC, PBC and healthy controls. Fraction of MAIT TCR clonotypes in blood of 504 PSC, 63 PBC and 904 healthy controls predicted by Seq2MAIT. Statistical significance was evaluated by Mann-Whitney test in Python using statannotions package, ns > 0.05, $^{****}p \leq 0.0001$.

Supplementary Table 1. Clinical details of scRNA-seq cohort at liver transplantation

Patient ID	Disease	Age (years)	Sex (M/F)	IBD status	UDCA (Y/N)	Disease stage	APRI	FIB-4	MELD Na	Hb (g/dL)	Platelets (10^9/L)	Leuko (10^9/L)	INR
1	PSC	38	M	UC	Y	late	0.8	1.4	7	14.3	220	5.4	0.9
2	PSC	65	F	CD	N	late	21.1	31.1	25	8.4	36	21.3	1.3
3	PSC	37	M	—	Y	late	4.7	8.6	19	11.2	86	4.7	1.2
4	PSC	44	M	UC	Y	early	1.1	4.6	15	12.6	218	10.2	1.3
5	PSC	19	M	CD	Y	early	1.7	1.1	14	11.2	174	4.5	1.2
6	PSC	33	M	UC	N	late	2.5	4.7	23	9.0	159	5.0	1.4
7	PSC	56	M	UC	N	late	9.4	14.8	21	13.0	102	5.5	1.6
8	PSC	63	M	—	Y	early	1.0	4.3	11	12.7	123	6.3	1.1
9	PSC	36	M	UC	N	late	2.2	1.9	18	13.9	216	5.5	1.3
10	PSC	70	M	UC	N	late	0.6	3.5	10	11.7	121	4.9	1.2
11	PSC	28	F	UC	N	early	0.7	0.8	25	12.1	290	5.7	1.1
12	PSC	22	M	UC	Y	early	1.2	0.6	14	13.3	375	9.1	1.1
13	PBC	45	F	—	N	late	2.0	5.1	12	14.1	74	10.6	1.1
14	PBC	54	F	—	Y	late	3.1	8.4	19	13.2	90	4.2	1.4
15	PBC	46	F	—	N	late	3.4	7.9	21	10.3	115	4.0	1.6
16	PBC	44	M	—	N	late	1.4	1.8	9	12.8	296	6.0	0.9
17	PBC	43	F	—	Y	early	1.7	5.2	10	10.2	96	5.7	1.1
18	PBC	57	M	—	N	late	2.3	5.8	11	14.5	155	3.6	1.1
19	PBC	69	F	—	Y	late	0.9	4.8	15	7.7	139	4.3	1.3
20	PBC	52	M	—	N	late	3.7	8.2	7	11.9	46	5.2	1.1
21	PBC	53	F	—	Y	late	3.9	12.5	12	9.2	66	3.5	1.2
22	ALD	55	F	—	N	late	1.2	4.4	19	13.4	112	8.2	1.7
23	ALD	62	F	—	N	late	0.5	2.0	10	13.1	159	8.9	1.4
24	ALD	56	F	—	N	late	1.4	5.3	19	10.0	83	4.8	1.5

Patient ID	Disease	Sodium (mmol/L)	AST (U/L)	ALT (U/L)	Protein (g/L)	ALB (g/dL)	bilirubin (μmol/L)	CRP (mg/L)	γGT (U/L)	ALP (U/L)	Creatinine (μmol/L)	Dialysis (Y/N)
1	PSC	141	87	119	82	43	19	1.2	444	272	82	N
2	PSC	136	304	311	39	32	916	51.0	313	351	52	N
3	PSC	132	200	100	70	32	90	46.0	450	665	71	N
4	PSC	140	97	18	99	36	73	21.0	1689	339	52	N
5	PSC	138	116	138	69	39	73	3.0	186	176	69	N
6	PSC	137	162	51	79	32	409	32.0	88	286	101	N
7	PSC	138	384	202	62	30	222	28.0	213	231	69	N
8	PSC	143	50	35	71	33	25	33.0	93	393	113	N
9	PSC	139	187	267	72	49	149	4.4	440	1110	88	N
10	PSC	139	28	21	80	40	8	8.0	277	233	104	N
11	PSC	133	79	103	67	41	34	1.0	529	252	51	Y
12	PSC	142	176	263	80	44	86	3.0	698	541	65	N
13	PBC	141	59	49	75	39	54	3.1	377	328	52	N
14	PBC	135	111	63	72	34	104	28.0	74	199	80	N
15	PBC	136	157	64	66	26	166	3.9	119	598	49	N
16	PBC	139	171	192	79	42	35	1.6	523	1776	67	N
17	PBC	137	64	30	80	35	36	13.8	65	190	77	N
18	PBC	138	143	81	86	40	44	13.8	456	620	89	N
19	PBC	134	52	29	51	21	36	15.1	53	159	77	N
20	PBC	135	68	87	69	40	11	3.0	286	159	70	N
21	PBC	139	103	44	56	34	48	26.8	368	386	50	N
22	ALD	137	55	38	63	33	102	4.9	35	200	60	N
23	ALD	130	26	26	77	40	18	21.0	274	137	65	N
24	ALD	137	47	36	68	39	14	1.6	194	120	207	N

Supplementary Table 1 Abbreviations: ALB, albumin; ALD, alcohol-related liver disease; ALT, alanine aminotransferase; ALP, alkaline phosphatase; APRI, aspartate aminotransferase to platelet ratio index; AST, aspartate transaminase; CD, Crohn's disease; CRP, C-reactive protein; FIB-4, fibrosis-4 index; γGT, gamma-glutamyl transferase; Hb, hemoglobin; IBD, inflammatory bowel disease; INR, international normalized ratio; leuko, leukocytes; MELD-Na, model for end-stage liver disease sodium, neg, negative; PBC, primary biliary cholangitis; PSC, primary sclerosing cholangitis; UC, ulcerative colitis; UDCA, ursodeoxycholic acid; (–), no result; early-stage disease = FIB-4 < 1.3 for < 65 years, < 2.0 for ≥ 65 years; later-stage disease = FIB-4 ≥ 1.3 for < 65 years, ≥ 2.0 for ≥ 65 years.

Supplementary Table 2. Clinical details of IHC liver staining cohort at liver transplantation

Patient ID	Disease	Age (years)	Sex (M/F)	IBD status	UDCA (Y/N)	Disease Stage	APRI	FIB-4	MELD Na	Hb (g/dL)	Platelets (10^9/L)	Leuko (10^9/L)	INR
1	PSC	38	M	UC	Y	late	0.8	1.4	7	14.3	220	5.4	0.9
3	PSC	37	M	neg	Y	late	4.7	8.6	19	11.2	86	4.7	1.2
14	PBC	54	F	neg	Y	late	3.1	8.4	20	13.2	90	4.2	1.4
23	ALD	62	F	neg	N	late	0.5	2.0	10	13.1	159	8.9	1.4
25	PSC	56	M	neg	N	early	0.2	0.7	6	13.4	366	8.5	1.0
26	PSC	41	M	UC	N	late	0.8	1.4	8	13.4	287	8.9	1.2
27	PSC	43	M	neg	Y	late	1.2	1.9	20	15.8	195	5.6	1.1
28	PSC	42	M	UC	N	early	0.2	0.6	7	15.5	348	6.7	1.1
29	PSC	40	M	UC	Y	late	0.8	1.4	18	14.9	305	7.5	1.1
30	PSC	53	M	UC	Y	late	1.0	1.7	9	13.8	282	8.0	1.0
31	PSC	32	M	UC	Y	late	4.5	6.4	7	12.2	60	2.2	1.0
32	PSC	48	F	neg	Y	early	0.5	0.9	12	13.5	445	3.5	1.1
33	PSC	33	F	UC	Y	late	5.1	6.2	22	10.4	68	3.6	1.4
34	PSC	58	M	neg	N	late	2.6	8.3	22	12.2	81	4.0	1.5
35	PSC	47	F	UC	N	late	5.2	5.9	24	12.0	148	7.5	1.6
36	PSC	32	M	UC	N	late	7.7	11.0	15	10.1	72	2.1	1.2
37	PSC	66	M	neg	N	late	1.5	4.4	15	13.1	153	7.8	1.1
38	PSC	67	M	UC	Y	late	1.9	7.2	19	11.6	184	6.4	1.2
39	PSC	29	M	UC	Y	late	1.2	1.7	24	8.9	155	4.4	1.5
40	PSC	39	M	UC	N	early	0.2	0.4	6	15.3	466	14.7	1.0
41	PSC	55	F	UC	Y	late	1.1	1.8	7	12.9	246	7.9	1.1
42	PSC	54	F	neg	N	late	0.7	1.3	9	11.8	210	9.4	0.9
43	MASH	64	F	neg	N	late	1.2	3.9	8	9.5	141	6.8	1.2
44	MASH	55	M	neg	N	late	0.6	2.7	11	12.2	150	7.3	1.3
45	MASH	70	M	neg	N	late	0.4	2.1	8	8.6	210	5.2	1.2
46	ALD	70	M	neg	N	late	2.0	16.5	29	8.6	36	3.1	2.5
47	ALD	58	M	neg	N	late	0.7	4.8	11	11.7	106	6.2	1.3
48	ALD	56	M	neg	N	late	1.6	5.6	18	10.4	138	7.3	1.2
49	PBC	66	F	neg	N	late	1.6	7.0	15	8.0	68	1.4	1.3
50	PBC	72	F	neg	N	late	0.8	4.4	23	10.4	82	9.0	2.1
51	PBC	58	F	neg	N	late	6.9	9.5	17	9.7	98	4.7	1.2
52	PBC	72	F	neg	Y	late	2.1	8.2	14	11.9	91	3.3	1.3
53	PBC	53	M	neg	Y	late	2.1	5.0	25	9.5	173	7.2	1.7
54	PBC	56	M	neg	N	late	3.0	4.7	16	12.7	138	3.9	0.9
55	PBC	64	F	neg	Y	late	15.1	23.5	30	7.6	48	4.6	2.2
56	PBC	70	F	neg	Y	late	1.5	5.2	21	10.6	137	7.3	1.7
57	PBC	71	F	neg	Y	late	1.8	6.6	15	10.8	125	4.2	1.3

Patient ID	Disease	Sodium (mmol/L)	AST (U/L)	ALT (U/L)	Protein (g/L)	ALB (g/dL)	bilirubin (μmol/L)	CRP (mg/L)	γGT (U/L)	ALP (U/L)	Creatinine (μmol/L)	Dialysis (Y/N)
1	PSC	141	87	119	82	43	19	1.2	444	272	82	N
3	PSC	132	200	100	70	32	90	46.0	450	665	71	N
14	PBC	135	111	63	72	34	104	28.0	74	199	80	N
23	ALD	130	26	26	77	40	18	21.0	274	137	65	N
25	PSC	140	43	84	72	45	7	0.6	100	134	67	N
26	PSC	143	110	119	70	46	14	1.1	537	255	82	N
27	PSC	140	113	179	76	48	458	0.7	306	166	81	N
28	PSC	139	30	37	75	47	7	1.2	30	73	68	N
29	PSC	137	120	120	83	45	279	50.0	292	505	77	N
30	PSC	145	143	264	74	44	37	4.6	1107	528	69	N
31	PSC	140	136	128	68	38	20	3.1	236	344	64	N
32	PSC	144	79	82	69	43	55	1.7	229	325	66	N
33	PSC	134	121	89	52	27	248	23.0	30	281	53	N
34	PSC	132	107	85	74	22	122	17.0	137	188	56	N
35	PSC	136	271	214	67	31	326	16.0	277	663	61	N
36	PSC	140	278	127	79	35	87	14.0	1123	722	50	N
37	PSC	134	111	117	87	35	30	1.0	500	188	108	N
38	PSC	136	179	82	79	36	100	8.9	250	494	116	N
39	PSC	131	90	102	95	27	189	65.0	153	346	33	N
40	PSC	135	40	61	80	41	10	72.0	478	407	68	N
41	PSC	143	93	132	82	44	16	10.0	832	393	51	N
42	PSC	139	54	115	75	41	14	5.7	1411	613	119	N
43	MASH	135	59	48	78	41	9	4.0	186	74	76	N
44	MASH	136	46	38	54	26	22	28.0	73	112	95	N
45	MASH	127	37	33	74	36	12	3.7	190	89	79	N
46	ALD	137	36	18	55	44	98	12.0	56	89	167	N
47	ALD	135	39	20	59	31	29	13.0	172	137	103	N
48	ALD	132	112	66	70	32	24	18.0	164	143	140	N
49	PBC	132	33	21	62	35	22	12.0	97	168	120	N
50	PBC	130	23	21	49	34	38	67.0	11	37	41	N
51	PBC	138	237	216	81	31	120	16.0	379	598	52	N
52	PBC	139	67	42	71	26	55	24.0	79	212	84	N
53	PBC	130	162	98	85	17	147	17.0	73	252	75	N
54	PBC	137	188	260	80	43	47	4.0	1261	699	134	N
55	PBC	138	254	159	53	38	680	28.0	24	91	56	N
56	PBC	131	71	48	53	32	115	13.0	38	165	56	N
57	PBC	139	80	48	60	23	52	42.0	57	441	92	N

Supplementary Table 2 Abbreviations: ALB, albumin; ALD, alcohol-related liver disease; ALT, alanine aminotransferase; ALP, alkaline phosphatase; APRI, aspartate aminotransferase to platelet ratio index; AST, aspartate transaminase; CRP, C-reactive protein; FIB-4, fibrosis-4 index; γGT, gamma-glutamyl transferase; Hb, hemoglobin; IBD, inflammatory bowel disease; INR,

international normalized ratio; leuko, leukocytes; MASH, metabolic dysfunction-associated steatohepatitis; MELD-Na, model for end-stage liver disease sodium, neg, negative; PBC, primary biliary cholangitis; PSC, primary sclerosing cholangitis; UC, ulcerative colitis; UDCA, ursodeoxycholic acid; early-stage disease = FIB-4 < 1.3 for < 65 years, < 2.0 for ≥ 65 years; later-stage disease = FIB-4 ≥ 1.3 for < 65 years, ≥ 2.0 for ≥ 65 years.

MAIT17 TCR reporter line	TCR chain	V-gene	CDR3 Sequence	J-gene
MAIT17.1	Alpha	*TRAV1-2*	CAVRDSNYQLIW	*TRAJ33*
	Beta	*TRBV6-5*	CASSPTENTEAFF	*TRBJ1-1*
MAIT17.2	Alpha	*TRAV1-2*	CAVMDSNYQLIW	*TRAJ33*
	Beta	*TRBV6-2*	CASSYRGGSGANVLTF	*TRBJ2-6*
MAIT17.3	Alpha	*TRAV1-2*	CAVMDSSYKLIF	*TRAJ12*
	Beta	*TRBV6-1*	CASTSSEVGEQYF	*TRBJ2-7*
MAIT17.4	Alpha	*TRAV1-2*	CAVKDSNYQLIW	*TRAJ33*
	Beta	*TRBV20-1*	CSQTSGRINEQFF	*TRBJ2-1*
MAIT17.5	Alpha	*TRAV1-2*	CAVLDSNYQLIW	*TRAJ33*
	Beta	*TRBV3-1*	CASSRSGSETDTQYF	*TRBJ2-3*
MAIT17.6	Alpha	*TRAV1-2*	CAALDSNYQLIW	*TRAJ33*
	Beta	*TRBV15*	CATSRTTGEGYNEQFF	*TRBJ2-1*
MAIT17.7	Alpha	*TRAV1-2*	CAPMDSNYQLIW	*TRAJ33*
	Beta	*TRBV6-1*	CASSELAGADTQYF	*TRBJ2-3*
MAIT17.8	Alpha	*TRAV1-1*	CAVRDFPPNSGYSTLTF	*TRAJ11*
	Beta	*TRBV6-6*	CASSYSEQRSNQPQHF	*TRBJ1-5*
MAIT17.9	Alpha	*TRAV29/DV5*	CAASSGNTGKLIF	*TRAJ37*
	Beta	*TRBV7-9*	CASSLLRDLGGYTF	*TRBJ1-2*
MAIT17.10	Alpha	*TRAV13-1*	CAVLNRDDKIIF	*TRAJ30*
	Beta	*TRBV6-1*	CASSEGLEAYNIQYF	*TRBJ2-4*

Supplementary Table 3. MAIT17 TCR sequences identified by TCR-seq used to generate MAIT17 reporters. TCR reporter lines with prototypic MAIT gene usage (*TRAV1-2*$^{+}$*TRAJ33*$^{+}$/*TRAJ12*$^{+}$) shown in purple. MAIT17-like TCR reporter lines (*TRAV1-2*$^{-}$*TRAJ33*$^{-}$/*TRAJ12*$^{-}$) shown in orange.
