# Supplementary material for: Intrahepatic T cell analysis reveals Th17 and MAIT17 enrichment in primary sclerosing cholangitis
Source: JHEP Rep. 2026 Apr 17;8(9):101857. doi: 10.1016/j.jhepr.2026.101857 (PMC13507853; doi:10.1016/j.jhepr.2026.101857)
Supplement: Supplementary file 3 [file mmc4.pdf]

Research article

JHEP Reports

Intrahepatic T cell analysis reveals Th17 and MAIT17 enrichment in primary sclerosing cholangitis

Authors

Lisa R.V. Brynjulfsen, Hesham ElAbd, Jonas Øgaard, …, Tom H. Karlsen, Espen Melum, Brian K. Chung

Correspondence

b.k.chung@medisin.uio.no (B.K. Chung).

Graphical abstract

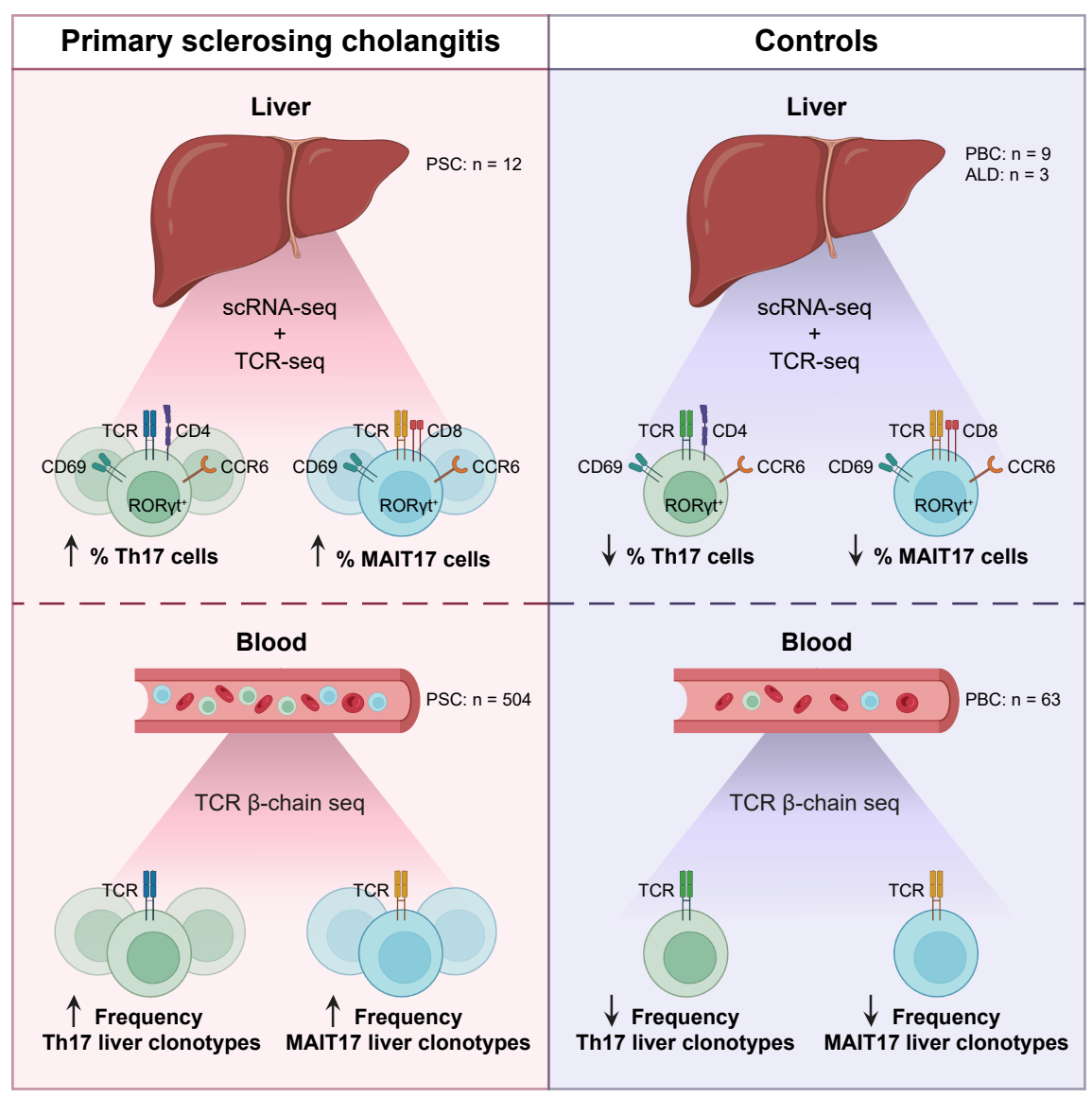

Highlights:

- Th17 and MAIT17 cells are enriched in PSC livers compared to disease controls.
- Liver Th17 and MAIT17 express markers associated with inflammation (*KLRB1*, *CCR6*, *RORC*, *IL23R*) and tissue-residency (*CD69*, *CXCR6*, *ITGAE*).
- Liver Th17 and MAIT17 TCR clonotypes are more frequent in blood of individuals with PSC than PBC cholestatic controls.
- Expanded MAIT17 TCR clonotypes recognize the known bacterial metabolite 5-OP-RU.

Impact and implications:

PSC is a progressive cholestatic biliary disease featuring liver T cell infiltration of unknown significance. To better define the role of T cells in PSC, we phenotyped intrahepatic T cells by scRNA-seq and detected increased proportions of tissue-resident Th17 and MAIT17 cells in PSC relative to disease controls. TCR sequencing also revealed that liver Th17 and MAIT17 TCR clonotypes were more frequent in blood from individuals with PSC compared with PBC. Collectively our results suggest that PSC is associated with a greater expansion of liver Th17 and MAIT17 which may contribute to the pathogenesis of PSC.

https://doi.org/10.1016/j.jhepr.2026.101857
© 2026 The Authors. Published by Elsevier B.V. on behalf of European Association for the Study of the Liver (EASL). This is an open access article under the CC BY license (http://creativecommons.org/licenses/by/4.0/).

Intrahepatic T cell analysis reveals Th17 and MAIT17 enrichment in primary sclerosing cholangitis

Lisa R.V. Brynjulfsen[1,2,3], Hesham ElAbd[4], Jonas Øgaard[1,2], Markus S. Jördens[1,2,3,5], Amber G. Bozward[6,7,8], Daniel Kearns[6,7,9], Xiaojun Jiang[1,2], Trine Folseraas[1,3,10], Palak J. Trivedi[6,7,9], Ye H. Oo[6,7,8], Andre Franke[4], Tom H. Karlsen[1,2,3,10], Espen Melum[1,2,3,10,11], Brian K. Chung[1,2,3,*]

JHEP Reports **2026**. vol. 8 | 1–12

Check for updates

Background & Aims: Primary sclerosing cholangitis (PSC) is an immune-mediated liver disease where the exact contribution of T cells is unclear. To deeply characterize intrahepatic T cells in PSC, we combined single-cell RNA sequencing (scRNA-seq) with T cell receptor sequencing (TCR-seq) and functional confirmation, using primary biliary cholangitis (PBC) and alcohol-related liver disease (ALD) as disease controls.

Methods: Intrahepatic T cells from 12 PSC, nine PBC and three ALD explants were enriched by negative selection, viability-sorted and analyzed by scRNA-seq for total gene and TCR expression. T helper 17 (Th17) and IL-17$^+$ mucosal-associated invariant T cell (MAIT17) proportions were analyzed by flow cytometry and liver immunohistochemistry. Liver TCRs in blood were evaluated in an independent PSC, PBC and healthy cohort previously analyzed by TCR β-chain sequencing. Antigenicity of MAIT TCR-transduced reporters was assessed using MAIT-specific tetramers (5-OP-RU-MR1) and MR1$^+$H69 cholangiocyte co-cultures.

Results: ScRNA-seq of 40,897 intrahepatic T cells revealed increased Th17/MAIT17 proportions in PSC livers compared with PBC (1.9-fold) and ALD (3.5-fold). Liver Th17 and MAIT17 expressed tissue-resident markers (*CD69*, *CXCR6*, *ITGAE)* and relative abundances measured by scRNA-seq correlated with proportions detected by flow cytometry. Immunohistochemistry liver staining of a validation cohort (n = 37) showed higher fractions of RORγt$^+$ cells in PSC and greater cumulative frequencies of liver Th17 and MAIT17 TCR clonotypes in PSC blood compared with PBC. Seven of ten TCR reporters representing the most-common and expanded MAIT17 clonotypes in PSC livers bound 5-OP-RU-MR1 tetramer and were activated by 5-OP-RU-loaded MR1$^+$H69 cholangiocytes.

Conclusions: Multimodal analysis of intrahepatic T cells in PSC revealed a novel enrichment of Th17 and MAIT17 which may reflect increased IL-17 polarization of T cells within the PSC microenvironment.

© 2026 The Authors. Published by Elsevier B.V. on behalf of European Association for the Study of the Liver (EASL). This is an open access article under the CC BY license (http://creativecommons.org/licenses/by/4.0/).

Introduction

Primary sclerosing cholangitis (PSC) is an inflammatory biliary disorder with no effective medical treatments limiting the progression of disease. Though rare (0–34.7/100,000 individuals globally),[1] individuals with PSC represent a serious unmet clinical need as they often require liver transplantation, experience reduced quality of life and are at significant risk of cancer development particularly cholangiocarcinoma.[2] Typical features of PSC include fibrosis, bile duct strictures, and cirrhosis, and 60–80% of patients have a concurrent inflammatory bowel disease (IBD).[1,3] Strong genetic associations to variants encoding the human leukocyte antigen complex, which present antigens to T cells, and genes involved in T cell function (*e.g. CCL20*, *CD28*, *IL2RA*, *IL2*, *IL21*) have been repeatedly reported and collectively suggest that T cells are critical in PSC pathogenesis.[3] Activating T cell antigens in PSC are unknown but reports of a substantial T cell and B cell overlap among the gut and liver compartment suggests that antigens circulating among the gut–liver axis may drive lymphocyte expansion in both microenvironments.[4,5]

Previous studies on intrahepatic T cell subsets in PSC have found tissue-resident CD8$^+$ T cells to be the most abundant lymphocyte subset overall[6] whereas IL-17$^+$CD4$^+$ T cells (Th17) are the most prevalent tissue-resident CD4$^+$ effector memory (EM) subset within PSC bile ducts.[7] Regulatory T cells (Tregs) involved in dampening inflammation are reportedly decreased in PSC liver explants compared with primary biliary cholangitis (PBC)[8] and CD4$^+$CD28$^-$ T cells associated with chronic antigen and cytokine activation are elevated in PSC livers compared to non-PSC controls.[9] These previous studies support the earlier genetic findings that T cells are involved in PSC pathogenesis but were biased towards known cell types as the primary methodologies utilized (*e.g.* flow cytometry and

* Corresponding author. Address: Norwegian PSC Research Center, Research Institute of Internal Medicine, Division of Surgery and Specialized Medicine, Oslo University Hospital Rikshospitalet, Postboks 4950 Nydalen, N-0424 Oslo, Norway.
E-mail address: b.k.chung@medisin.uio.no (B.K. Chung).
https://doi.org/10.1016/j.jhepr.2026.101857

immunohistochemistry [IHC]) require the preselection of relative few lineage markers that may have limited the analysis to known cell types and features.

To improve upon previous studies, recent immunophenotyping efforts have leveraged more granular RNA technologies, including single-cell RNA sequencing (scRNA-seq), single-nuclei RNA seq (snRNA-seq) and spatial transcriptomics.[10,11] These technologies enable deeper global profiling of the entire transcriptome, increasing phenotyping precision and reducing preselection bias compared with historical approaches.[12] Using a combination of scRNA-seq and flow cytometry to define T cell subsets, Poch and Krause *et al.*[13] identified a liver-resident population of naïve-like $CD4^+$ T cells expanded in PSC that preferentially differentiate into Th17 cells under polarizing *in vitro* conditions. Andrews and Nakib *et al.*[14] applied several multiomic modalities (scRNA-seq, snRNA-seq, spatial transcriptomics) to profile the entire liver compartment in patients with PSC and disease controls and also showed an expansion of $CD4^+$ T cells that co-localized with myeloid subsets. Although the main findings in these studies were not immunological, these data suggest that specific $CD4^+$ T cells have a role in pathogenesis but in both studies, scRNA-seq was implemented on a limited number of disease controls and protein-level tissue confirmation and functional validation of specific T cell clonotypes were not investigated.

As identification of enriched T cell subsets and confirmation of their antigen reactivity may broaden our understanding of the pathophysiological mechanisms underlying PSC, we aimed to determine if T cell subsets in PSC livers are enriched compared with disease controls by leveraging scRNA-seq, T cell receptor sequencing (TCR-seq), flow cytometry, and liver IHC. We further assessed if liver T cell clonotypes are detected in peripheral circulation using PSC, PBC, and healthy blood samples previously analyzed by T cell receptor (TCR) β-chain sequencing[15] and if expanded liver T cell clonotypes found across multiple individuals with PSC recognize a common antigen.

Patients and methods

Patient samples

Liver sections from individuals undergoing liver transplantation with PSC (n = 12), PBC (n = 9), and ALD (n = 3) were collected immediately following explantation for isolation of liver mononuclear cells (LMC). Fixed explant liver tissue from PSC (n = 20), PBC (n = 10), alcohol-related liver disease (ALD, n = 4) and metabolic dysfunction-associated steatohepatitis (MASH, n = 3) were analyzed by IHC. Blood samples from PSC (n = 504), PBC (n = 63), and healthy controls (n = 904) were assessed by TCR β-chain sequencing. This study was approved by the Regional Committees for Medical and Health Research Ethics of South East Norway (reference numbers: 15368 and 18221) and by the local ethical committees at the University of Birmingham (LREC #06-Q2702-61) and University of Kiel (D441/16, D474/12, A161/08, A103/14, and A148/14) and conducted in accordance with the Declaration of Helsinki and Istanbul. All study participants provided written informed consent before study inclusion.

ScRNA-seq and TCR-seq

ScRNA-seq and TCR-seq was performed using Chromium Next GEM Single Cell 5′ version 1.1 kits or Chromium Next GEM Single Cell 5’ v2 kits following the manufacturer’s instructions (User guide CG000207 Rev E and User guide CG000331 Rev D, 10x Genomics, Pleasanton, CA, USA) and batch corrected for kit variations (see Supplementary Materials & Methods). Enriched T cells (8,280–15,000 per reaction) were loaded per channel onto Chromium Next GEM G or K chips for GEM generation using a Chromium X/iX (10x Genomics). cDNA libraries were sequenced on a NovaSeq (Illumina, San Diego, CA, USA) using SP or S4 flow cells at the recommended minimum sequencing depth of 20,000 reads per cell for total gene libraries and 5,000 read pairs per cell for TCR libraries. Sequencing data from scRNA-seq can be explored at RIIM Data Explorer (https://riim-data.no/index2.html).

Liver TCR-seq

For TCR-seq of liver T cells, filtered contig annotation outputs from the Cell Ranger multi pipeline (10x Genomics) containing single and paired TCR αβ chain sequences were assigned to individual patients and scRNA-seq T cell clusters using R version 4.3.2 (R Foundation for Statistical Computing, Vienna, Austria) and Immunarch version 0.9.0.[17] Clonotypes were defined by the amino acid sequence of the TCR αβ chain complementarity-determining region 3 (CDR3). Expanded clonotypes were defined as cells expressing the identical TCR clonotype detected in at least two cells. For each cluster, the percentage of expanded cells was calculated as the number of expanded cells divided by the total number of TCR per sample.

Statistical analysis

All values shown represent the mean ± standard deviation unless otherwise stated. Mann-Whitney *U* test was used for comparisons between two groups and Kruskal-Wallis test followed by Dunn’s test was used for comparisons with three groups. Values of $p \leq 0.05$ for Mann-Whitney *U* test and *p* values adjusted for Dunn’s multiple comparisons ≤ 0.05 were considered statistically significant. Correlations and *p* values between scRNA-seq Th17 and $IL17^+$ mucosal-associated invariant T cell (MAIT17) frequencies and flow cytometry were calculated using two-tailed Pearson analysis. Unless otherwise stated, statistical analyses were performed using Prism version 10.0.3 (GraphPad, San Diego, CA, USA).

Results

Expansion of Th17/MAIT17 cells in livers of individuals with PSC

To investigate the phenotype of intrahepatic T cells in PSC (n = 12) compared with disease controls (PBC: n = 9, ALD: n = 3; Table 1; Table S1), we enriched T lymphocytes from LMC and assessed total gene and TCR expression using 5′ scRNA-seq and TCR-seq (Fig. 1A). In total, 40,897 intrahepatic T cells passed quality control and clustering by differential gene expression revealed 13 T cell subsets (Fig. 1B) that coarsely

Table 1. Clinical overview of the scRNA-seq cohort at time of liver transplantation.

	PSC (n = 12)	PBC (n = 9)	ALD (n = 3)
Age	42.6 (19–70)	51.3 (43–69)	57.7 (55–62)
Males, n (%)	10 (83.3%)	3 (33.3%)	0 (0.0%)
Bilirubin, μmol/L	175 (8–916)	59 (11–166)	45 (14–102)
INR	1.2 (0.9–1.6)	1.2 (0.9–1.6)	1.5 (1.4–1.7)
ALT, U/L	136 (18–311)	71 (29–192)	33 (26–38)
ALP, U/L	404 (176–1,110)	491 (159–1,776)	152 (120–200)
Albumin, g/dl	38 (30–49)	35 (21–42)	37 (33–40)
Platelets, 10^9/L	177 (36–375)	120 (46–296)	118 (83–159)

Data are presented as mean (range) or n (%). ALD, alcohol-related liver disease; ALP, alkaline phosphatase; ALT, alanine aminotransferase; IBD, inflammatory bowel disease; INR, international normalized ratio; PBC, primary biliary cholangitis; PSC, primary sclerosing cholangitis.

segregated based on *CD4* (cluster 1, 3, 6, 11, 12), *CD8A* (cluster 2, 4, 7, 9, 10), or a combination of *CD4* and *CD8A* expression (cluster 5, 8, 13; Fig. 1C). Clustering of T cell subsets was consistent between both PSC and control patients with each cluster identified in every liver except cluster 13 (cycling) which was present in 23 of 24 explants (Fig. 1D; Fig. S4A).

Using canonical lineage markers from differential gene expression analysis (Fig. 1E), $CD4^+$ T cell subsets were annotated into $CD4^+$ EM1 (T_{EM}1), $CD4^+$ central memory (T_{CM}), $CD4^+$ T_{EM}2, Tregs, and T follicular-helper (T_{FH})-like cells whereas $CD8^+$ T cell subsets were annotated into innate-like $CD8^+$ cytotoxic (T_c), $CD8^+$ tissue-resident memory (T_{RM}), innate-like $\gamma\delta$ T cells, $CD8^+$ T_c, and $CD8^+$ $T_{EM}/\gamma\delta$ cells. Of the three subsets containing a mixture of both $CD4^+$ and $CD8^+$ cells, cluster 5 expressed *CCR6*, *RORC*, *IL23R*, and *KLRB1* indicative of both Th17 and MAIT17 cells whereas the two remaining clusters were classified as naïve and cycling.

After defining the intrahepatic T cell compartment in the explanted livers, we examined the relative proportions of each T cell subset in PSC livers compared with disease controls (Fig. 1F). Cluster 5 containing Th17 and MAIT17 cells was increased 1.9-fold in PSC (11.9 ± 5.7%) compared with PBC (6.2 ± 2.7%, p = 0.023) and 3.5-fold higher in PSC compared with ALD (3.4 ± 1.9%, p = 0.0048). Relative proportions of Th17/MAIT17 in PSC livers were also elevated *vs*. healthy controls previously analyzed by scRNA-seq (PSC: 10.2 ± 3.5%, healthy controls: 4.4 ± 3.4%, p = 0.0069)[14] and the inclusion of eight PSC and two PBC liver samples from Andrews and Nakib *et al.*[14] further confirmed that Th17/MAIT17 abundances are increased in PSC livers compared with PBC disease controls (Fig. S5). In contrast, cluster 2 (innate-like $CD8^+$ T_c) was significantly reduced in PSC compared with ALD (PSC: 10.6 ± 10.9%, ALD: 37.4 ± 22.3%, p = 0.041) but not PBC (11.3 ± 7.7%, p = 0.14). No other clusters showed significant differences in abundance between PSC, PBC, and ALD by scRNA-seq. To determine if immune-related liver disorders jointly exhibited enrichment of T cell states, we merged the relative abundance values of each T cell cluster from PSC and PBC livers (Fig. 1F) and saw that Tregs (cluster 11) were increased in the immune-related liver diseases (PSC and PBC) compared with ALD (PSC and PBC: 5.1 ± 3.9%, ALD: 1.3 ± 1.1%, p = 0.011) and also harbored a greater proportion T_{FH}-like cells (cluster 12; PSC and PBC: 3.9 ± 1.7%, ALD: 0.8 ± 0.5%, p = 0.0010). Fibrosis-4 (FIB-4) and model for end-stage liver disease-sodium (MELD-Na) scores indicative of fibrosis and liver decompensation at transplantation did not correlate with any of the T cell proportions differentially abundant between PSC, PBC, and ALD livers (Fig. S4B). Collectively these results imply that T cells with an IL-17 signature are expanded in livers of individuals with PSC and that Tregs and T_{FH}-like cells, regulating inflammation and promoting B cell differentiation, are enriched in the PSC and PBC compared with ALD.

Intrahepatic Th17 and MAIT17 cells are distinct and elevated in PSC

To determine if specific gene profiles for Th17 and MAIT17 cells could be distinguished, we reclustered the combined Th17/MAIT17 cluster (cluster 5) and observed a separation of a *CD4*$^+$ Th17 and *CD8*$^+$ MAIT17 subset (Fig. 2A). Cells in the Th17 cluster featured higher expression of *CD4* and the co-stimulatory receptors *ICOS* and *CD40LG*,[18] whereas MAIT17 cells expressed the canonical MAIT genes *TRAV1-2* and *SLC4A10*,[19] and the T_c-related markers *NKG7*, *GZMK*, and *PRF1* (Fig. 2B).[20,21] Despite these clear differences, Th17 and MAIT17 cells shared a large phenotypic similarity including a robust expression of the proinflammatory IL-17$^+$ T cell markers *KLRB1*, *CCR6*, *RORC*, and *IL23R* (Fig. 2C). Abundance analysis (Fig. 2D) showed that Th17 cells were enriched in PSC livers compared with ALD (PSC: 6.9 ± 3.9%, ALD: 1.8 ± 1.6%, p = 0.020) but not compared with PBC livers (PBC: 4.0 ± 2.2%, p = 0.13) whereas MAIT17 cells were more abundant in PSC livers (4.9 ± 2.3%) compared with both PBC (2.2 ± 0.7%, p = 0.021) and ALD (1.6 ± 0.3%, p = 0.0061). Weak expression of the circulating T cell markers *CCR7*, *S1PR1*, and *SELL* was detected in a small proportion of Th17/MAIT17 cells whereas higher levels of the tissue-resident markers *CD69*, *CXCR6*, and *ITGAE* were observed in larger proportion of the Th17/MAIT17 cluster (Fig. 2E).[6] Th17/MAIT17 cluster split by disease revealed no difference in the proportions of Th17/MAIT17 expressing circulating or tissue-resident markers between PSC, PBC, and ALD livers (Fig. 2F). TCR-seq analysis of clonotypes defined by scRNA-seq as Th17 and MAIT17 showed a similar percentage of expanded clonotypes and cells assigned to expanded clonotypes among PSC and disease controls (Fig. S6).

Th17 and MAIT17 analyses using flow cytometry and immunohistochemistry staining

To assess if Th17 and MAIT17 were increased in PSC livers at the protein level, we first performed flow cytometry on a subset of LMC (n = 8) analyzed by scRNA-seq using $CD4^+CD161^+CCR6^+ROR\gamma t^+$ as markers for Th17 cells and

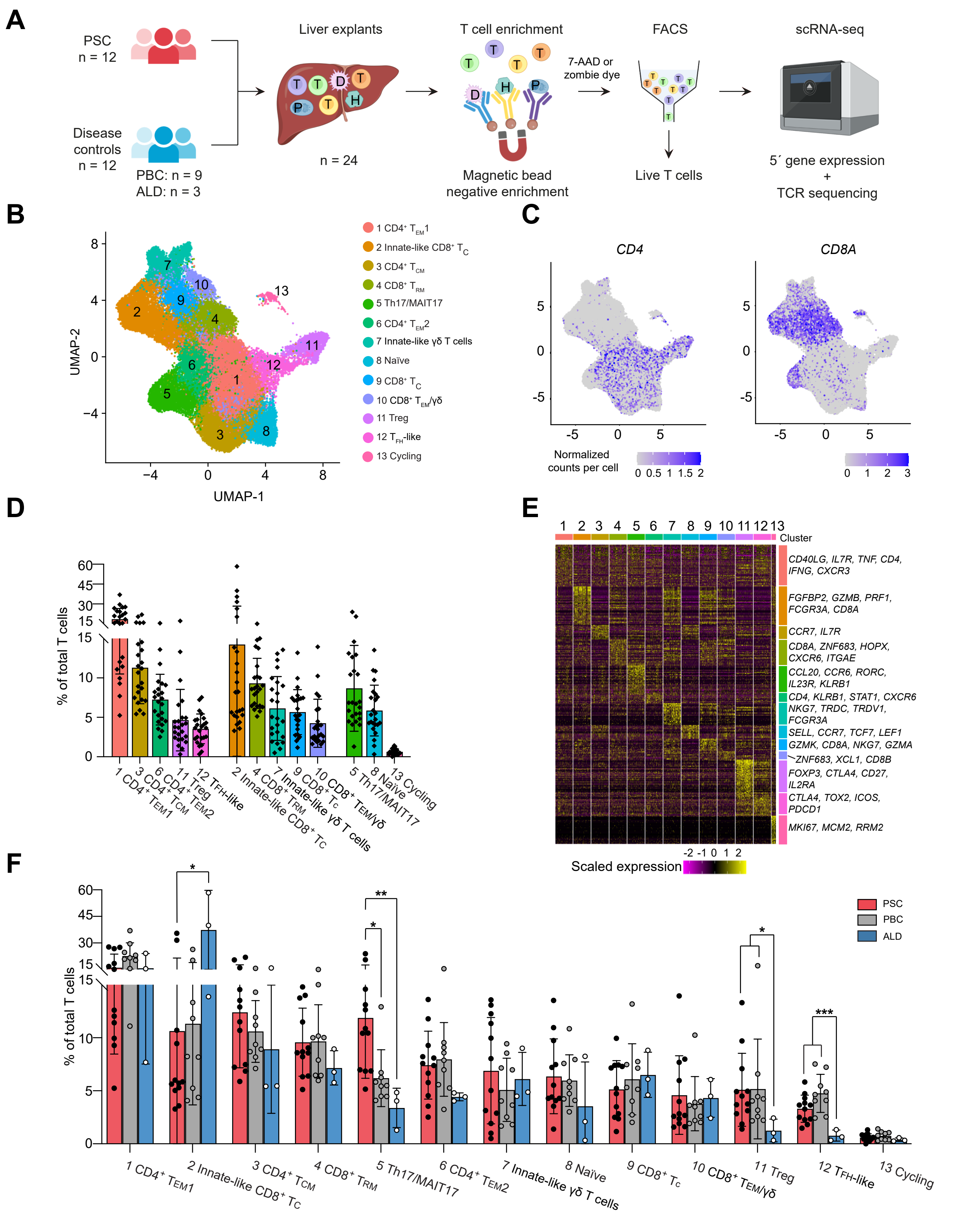

Fig. 1. Expansion of Th17/MAIT17 cells in livers of individuals with PSC. (A) Workflow for scRNA-seq of T cells from explanted livers of PSC (n = 12), PBC (n = 9) and ALD (n = 3). (B) UMAP of 40,897 T cells from all livers combined (PSC: 16,641 cells, PBC: 18,426 cells, ALD: 5,830 cells). (C) T cell UMAPs of *CD4* or *CD8A* expression. (D) Relative abundance of each T cell cluster relative to total T cells ordered into $CD4^+$ clusters, $CD8^+$ clusters, and clusters with both $CD4^+$ and $CD8^+$ cells. (E) Heatmap of top 40 differentially expressed genes for each cluster. Representative marker genes shown on the right. (F) Relative abundance of T cell clusters

Vα7.2$^+$CD161$^+$CCR6$^+$RORγt$^+$ for MAIT17 cells (Fig. 3A and Fig. S7). We observed a strong correlation in the relative proportion of Th17 cells (R = 0.81, p = 0.015) and MAIT17 cells (R = 0.83, p = 0.010) measured by flow cytometry and scRNA-seq supporting that liver Th17 and MAIT17 are increased in PSC (Fig. 3B). To confirm that Th17 and MAIT17 were enriched in PSC livers, we next quantified CD3 and RORγt co-expression by immunofluorescence (IF) and noted that the majority of RORγt$^+$ cells co-stained with CD3 (Fig. 3C, D; Fig. S8). IHC staining for CD3 and RORγt of 20 PSC and 17 liver disease controls (10 PBC, four ALD, three MASH; Fig. 3E; Table S2) in a validation cohort showed that proportions of CD3$^+$ cells per total cells were decreased in PSC compared with disease controls (PSC: 7.1 ± 3.2%, disease controls: 9.9 ± 3.2%, p = 0.008; Fig. 3F) but RORγt$^+$ cells in CD3$^+$ regions were significantly enriched in PSC (PSC: 28.0 ± 11.8%, disease controls: 19.0 ± 7.0%, p = 0.009). Collectively these protein-level data support our scRNA-seq findings that relative proportions of RORγt$^+$ cells are increased in PSC compared with disease controls.

Th17 TCR clonotypes and MAIT17 cells in the blood of individuals with PSC

Having confirmed increased Th17 and MAIT17 cells in PSC livers, we determined if the abundance of Th17 and MAIT17 clonotypes defined by TCR-seq were altered in blood samples from an independent cohort of PSC (n = 504), PBC (n = 63), and healthy controls (HC, n = 904) previously assessed by TCR β-chain sequencing (Fig. 4A).[15] We detected 218 of 843 total PSC Th17 liver clonotypes (25.9%) and 156 of 494 total PSC MAIT17 liver clonotypes (31.6%) across the TCR blood repertoire. Liver Th17 clonotypes constituted a greater median cumulative frequency of the blood TCR repertoire in PSC (PSC: 1.99×10^{-3}, HC: 1.74×10^{-3}, $p = 3.05 \times 10^{-4}$, Fig. 4B) whereas cumulative median frequencies of liver MAIT17 clonotypes in PSC blood were dramatically reduced in the peripheral TCR repertoire of HC (PSC: 0.5×10^{-2}, HC: 1.08×10^{-2}, $p = 2.08 \times 10^{-11}$).

In contrast to PSC, we detected 122 of 492 total PBC Th17 liver clonotypes (24.8%) in PBC blood samples (n = 63) and 71 of 252 total PBC MAIT17 liver clonotypes across the TCR blood repertoire of HC (n = 904). Median cumulative frequencies of PBC Th17 and MAIT17 liver clonotypes were both lower in PBC blood than HC (Th17 PBC: 1.25×10^{-3}, HC: 1.70×10^{-3}, $p = 1.06 \times 10^{-2}$; MAIT17 PBC: 6.4×10^{-4}, HC: 1.32×10^{-3}, $p = 7.02 \times 10^{-4}$; Fig. 4C). Interestingly, PSC Th17 and MAIT17 liver clonotypes were significantly elevated in PSC blood compared with PBC Th17 and MAIT17 liver clonotypes in PBC blood. (Th17 PSC: 1.99×10^{-3}, Th17 PBC: 1.25×10^{-3}, $p = 1.07 \times 10^{-3}$; MAIT17 PSC: 0.5×10^{-2}, MAIT17 PBC: 6.4×10^{-4}, $p = 4.81 \times 10^{-16}$; Fig. 4D). No difference in total MAIT TCR frequencies was detected between PSC and PBC blood samples using a deep learning MAIT predictive model (Seq2MAIT)[22] whereas both disease groups showed lower MAIT TCR frequencies than HC (PSC: 0.83×10^{-2}, PBC: 0.80×10^{-2}, HC: 1.01×10^{-2}; Fig. S9).

Expanded MAIT17 TCR clonotypes in PSC livers recognize 5-OP-RU

To validate that liver MAIT17 defined by scRNA-seq are *bona fide* MAIT cells that recognize the prototypical MAIT antigen 5-(2-oxopropylideneamino)-6-d-ribitylaminouracil (5-OP-RU), we selected the top 10 most prevalent and highly expanded MAIT17 TCR αβ-CDR3 detected by TCR-seq (Table S3) and generated MAIT17 TCR reporters (MAIT17.1 to MAIT17.10) using lentiviral transduction (Fig. 5A). Seven of 10 MAIT17 TCR (MAIT17.1 to MAIT17.7) utilized known MAIT TCR α-genes (*TRAV1-2*, *TRAJ33*, or *TRAJ12*)[23] whereas MAIT17.8 to MAIT17.10 utilized TCR genes not previously ascribed to MAIT cells (Table S3). As expected, MAIT17 reporters expressing the prototypic MAIT TCR α-genes stained positive with 5-OP-RU-MR1 tetramer but showed differing binding affinities as measured by tetramer-positive mean fluorescence intensity (MFI) values (Fig. 5B, C). MAIT17 reporters expressing unreported MAIT TCR α-genes did not stain with the 5-OP-RU-MR1 tetramer (MAIT17.8 to MAIT17.10). Tetramer-positive TCR reporters were activated in a dosage-dependent manner by 5-OP-RU-pulsed H69-MR1 cholangiocytes while tetramer-negative reporters remained eGFP negative (Fig. 5D, E). These findings confirm that MAIT cells identified by scRNA-seq express TCR which recognize and are stimulated by 5-OP-RU.

Discussion

Herein we performed scRNA-seq and TCR-seq on intrahepatic T cells from the largest number of individuals with PSC to date and identified 13 T cell subsets where Th17 and MAIT17 cells were increased and were consistent with findings detected at the protein level using flow cytometry, IF, and IHC staining.

It has previously been reported that Th17 cells are the most abundant CD4 T_{EM} in bile ducts of patients with PSC[7] and Poch and Krause *et al.*[13] identified a subset of enriched tissue-resident naïve-like CD4$^+$ T cells with the propensity to develop into Th17. Moreover, studies by Jeffery, Hunter and Humphreys *et al.*[24] suggest CD4 T cells polarize towards Th17 around biliary epithelium and Oo and Banz *et al.*[25] showed that inflamed and microbe-exposed biliary epithelium secrete CXCL10 and CCR6, recruiting Th17 cells via CXCR3 across hepatic sinusoids. Our findings significantly extend these previous observations and show for the first time that Th17 are

between PSC, PBC, and ALD, and between PSC + PBC (PSC and PBC merged into single disease group) and ALD. Comparisons between PSC + PBC and ALD are highlighted in bold. Statistical significance was evaluated using Kruskal-Wallis test followed by Dunn's test for comparison between three groups and Mann-Whitney *U* test for comparison between two groups, *p ≤0.05, **p ≤0.01, ***p ≤0.001. ALD, alcohol-related liver disease; D, dendritic cell; H, hepatocyte; MAIT17, *IL17*$^+$ mucosal-associated invariant T cell; P, plasma cell; PBC, primary biliary cholangitis; PSC, primary sclerosing cholangitis; scRNA-seq, single-cell RNA sequencing; T, T cell; T_c, cytotoxic T cell; T_{CM}, central memory T cell; T_{EM}, effector memory T cell; T_{FH}-like, follicular-helper like T cell; Th17, T helper type 17; Treg, regulatory T cell; T_{RM}, tissue-resident memory T cell; UMAP, uniform manifold approximation and projection.

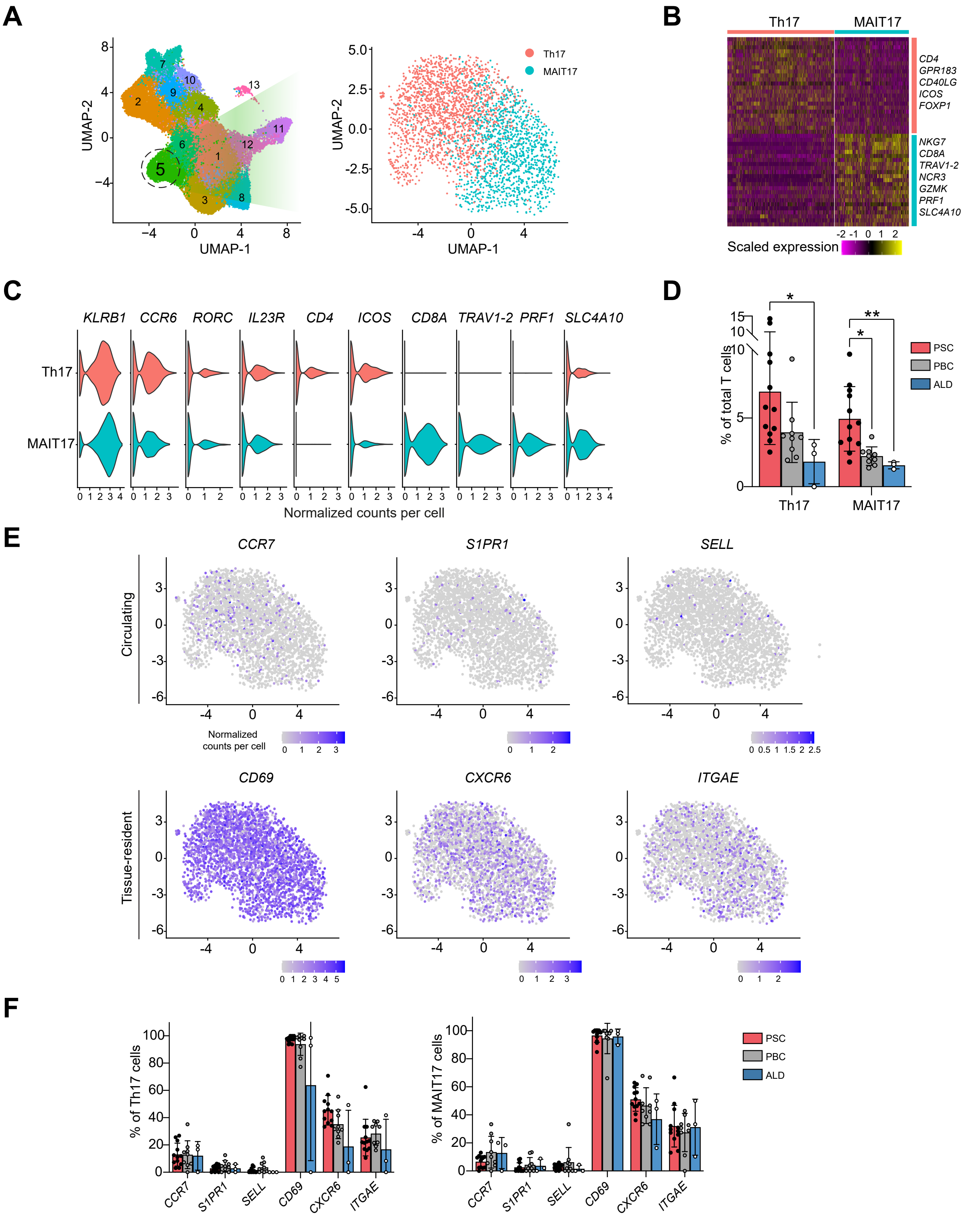

Fig. 2. Intrahepatic Th17 and MAIT17 cells are distinct and elevated in patients with PSC. (A) Reclustering of Th17/MAIT17 cluster from the total T cell compartment of PSC, PBC, and ALD explants. Th17/MAIT17 UMAP contains 3,039 cells from PSC (n = 12, 1,746 cells), PBC (n = 9, 1,044 cells), and ALD (n = 3, 249 cells). (B) Heatmap of top 25 differentially expressed genes for Th17 and MAIT17. Representative marker genes shown on the right. (C) Normalized expression counts of indicated genes in Th17 and MAIT17. (D) Abundance of Th17 and MAIT17 among PSC, PBC, and ALD calculated by scRNA-seq. (E) Expression of circulating

directly increased in PSC livers by scRNA-seq, flow cytometry, and IHC. A potential mechanism for this increase could relate to the association of PSC with IBD as peripheral blood mononuclear cells from patients with PSC-IBD stimulated by gut-derived microbiota showed greater Th17 differentiation and IL-17 secretion compared with peripheral blood mononuclear cells from patients with IBD and HC.[26,27] Furthermore, increased liver Th17 differentiation has also been observed in gnotobiotic mice inoculated with PSC-derived microbiota suggesting that enrichment of liver Th17 cells in PSC could be caused by disease-associated microbial strains.[28] Collectively, our data suggest that the gut–liver axis may be a key factor in driving the enrichment of Th17 cells in PSC livers.

In addition to Th17 cells, we also observed that liver MAIT17 cells were increased in PSC. These data support previous observations suggesting that intrahepatic MAIT cells localize around peribiliary regions and are activated by *Escherichia coli* via intrahepatic B cells and biliary epithelium.[29] These findings however contrast with previous reports showing PSC and non-PSC controls have similar abundances of liver MAIT cells when defined as TCR Vα7.2$^+$CD161$^+$ by flow cytometry or as TCR Vα7.2$^+$ by IHC.[30,31] These disparate results could reflect the ability of scRNA-seq to identify MAIT17 cells using multiple markers (*e.g. RORC*, *CCR6*, *IL23R*) instead solely by the expression of TCR Vα7.2 alone. As expression of TCR Vα7.2 is not exclusive to MAIT cells, identification using MR1-5-OP-RU tetramers is regarded as the most accurate TCR-specific method.[32] Another explanation for our findings is the potential classification of non-MAIT cells as MAIT17 as we saw that three of 10 TCR reporters generated from the MAIT17 cluster defined by scRNA-seq did not recognize the prototypical MAIT antigen. These non-MAIT cells share nearly identical gene profiles with prototypical MAIT17 cells and may therefore represent a MAIT17-like subset which possibly recognize a range of non-MAIT antigens. As MAIT17-like T cells (*TRAV1-2*$^-$, *TRAJ33*$^-$, *TRAJ12*$^-$, and *TRAJ20*$^-$) constitute 6.7–100% (2–117 total cells per patient) of the MAIT17 scRNA-seq cluster from different liver explants (data not shown), future large-scale screening of MAIT17-like TCR reporters may reveal the identity of novel antigens that promote IL-17$^+$ T cell differentiation.

Th17 and MAIT17 subsets in our data both expressed RORγt which induces transcription of the genes encoding for IL-17.[33] Although the exact role of IL-17$^+$ T cells is still to be determined in PSC, inhibition of RORγt has shown to protect against 3,5-diethoxycarbonyl-1,4-dihydrocollidine-induced liver injury in mice inoculated with mixture of *Klebsiella pneumoniae, Proteus mirabilis*, and *Enterococcus gallinarum*.[28] Increased circulating Th17 cells have also been reported in rheumatoid arthritis, multiple sclerosis, and psoriasis indicating that IL-17$^+$ T cells are a general disease feature of immune-mediated chronic disorders.[34–36] Our observations here and previous reports suggest that Th17 and MAIT17 depletion may be of potential benefit in PSC but as both cell types participate in the clearance of extracellular pathogens and mucosal homeostasis, targeting of Th17 and MAIT17 in PSC requires caution and further investigation.[37–40]

To determine if our scRNA-seq results from the liver were generalizable to other sites and a larger study cohort, we assessed the abundance of Th17 and MAIT17 clonotypes from PSC and PBC livers in blood of individuals with PSC, PBC, and HC. PSC liver Th17 clonotypes were enriched in PSC blood *vs*. HC similar to other immune-mediated chronic disorders[34–36] and also elevated compared with PBC liver Th17 in PBC blood. In contrast to Th17 liver clonotypes, PSC and PBC MAIT17 liver clonotypes were decreased in PSC and PBC blood compared with HC and predicted Seq2MAIT frequencies of MAIT clonotypes were also decreased in PSC and PBC blood compared with HC. These findings align with previous studies reporting lower frequencies of MAIT cells in PSC blood[30,41] as well as ALD, PBC, IBD, rheumatoid arthritis, and systemic lupus erythematosus,[41–43] suggesting that MAIT17 clonotypes may not be enriched in totality but could reflect increased IL-17 polarization of liver-resident MAIT cells or recruitment of circulating MAIT cells that acquire a tissue-resident MAIT17 phenotype in the PSC microenvironment.

Though we present the largest analysis of liver T cells in PSC by scRNA-seq and TCR-seq to date, including the greatest number of patients with PSC sampled from a considerable amount of liver explant material per patient (~100 g), our study is still limited by a relatively small sample size and partial assessment of each liver explant per patient which may yield an incomplete capture of all T cells. Future studies in larger cohorts using material from multiple locations per explant with deeper sequencing as pathogenic antigen-specific T cells in PSC may be rare. For instance in celiac disease, only 0.1–1.2% of the total CD4$^+$ T cells in intestinal biopsies of untreated patients with celiac disease were found to recognize gluten.[44] Future studies including larger numbers of early-stage patients are needed to ascertain if Th17 and MAIT17 are also elevated throughout PSC progression or increased as a result of secondary effects resulting from chronic inflammation and cirrhosis. As our findings rely on single-cell methodologies, the spatial location of the different T cell subsets identified by scRNA-seq and potential interacting cell types are unknown but are key to unlocking relevant T cell phenotypes as Tregs in portal tracts but not lobular regions have already known to be differentially abundant in PSC and PBC livers.[8] Future efforts implementing the latest subcellular spatial technologies in combination with deep cellular multi-marker phenotyping at the protein level by multiplex tissue staining will greatly improve the identification of disease-relevant T cell subsets within specific microanatomical locations and help delineate the key cell types mediating the production and presentation of lymphocyte antigens in PSC.

(*CCR7*, *S1PR1*, *SELL*) and tissue-resident (*CD69*, *CXCR6*, *ITGAE*) markers in Th17/MAIT17 of PSC, PBC and ALD liver explants. (F) Proportion of Th17 and MAIT17 expressing the indicated circulating and tissue-resident markers split by disease. Statistical significance was evaluated using Kruskal-Wallis test followed by Dunn's test, *p ≤0.05, **p ≤0.01. ALD, alcohol-related liver disease; MAIT17, *IL17*$^+$ mucosal-associated invariant T cell; PBC, primary biliary cholangitis; PSC, primary sclerosing cholangitis; Th17, T helper type 17; UMAP, uniform manifold approximation and projection.

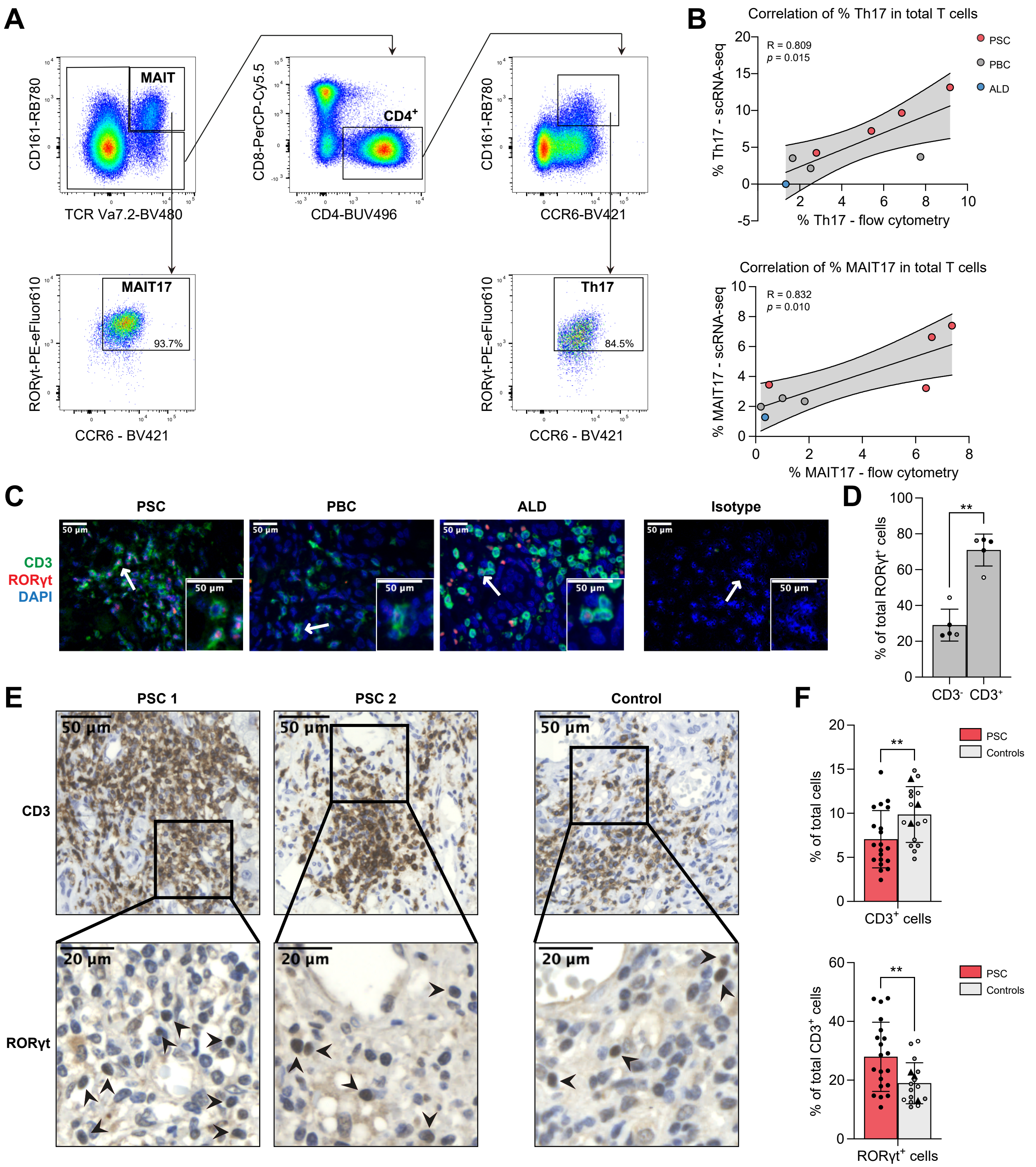

Fig. 3. Th17 and MAIT17 analyses using flow cytometry and immunohistochemistry staining. (A) Gating strategy for MAIT17 and Th17 gated from live CD3$^+$ TCRγδ$^-$ T cells derived from explanted livers of patients (four PSC, three PBC and one ALD). (B) Correlation between Th17 and MAIT17 abundance measured by scRNA-seq and flow cytometry. (C) CD3 and RORγt IF of liver tissue from PSC and disease controls. (D) Relative abundance of CD3$^-$RORγt$^+$ and CD3$^+$RORγt$^+$ cells in three PSC (black dots) and two disease controls (PBC: gray dot, ALD: white dot). (E) CD3 and RORγt IHC staining of liver tissue from PSC and disease control. (F) Relative abundance of CD3$^+$ cells in total cells and RORγt$^+$ cells in CD3$^+$ cells between PSC and disease controls (PBC: gray dots, ALD: white dots, MASH: black triangles). Statistical significance of correlation was evaluated using two-tailed Pearson correlation. Mann-Whitney *U* test was used for comparison between two groups, $^{**}p \leq 0.01$. ALD, alcohol-related liver disease; MAIT, mucosal-associated invariant; MAIT17, *IL17*$^+$ mucosal-associated invariant T cell; PBC, primary biliary cholangitis; PSC, primary sclerosing cholangitis; scRNA-seq, single-cell RNA sequencing; Th17, T helper type 17.

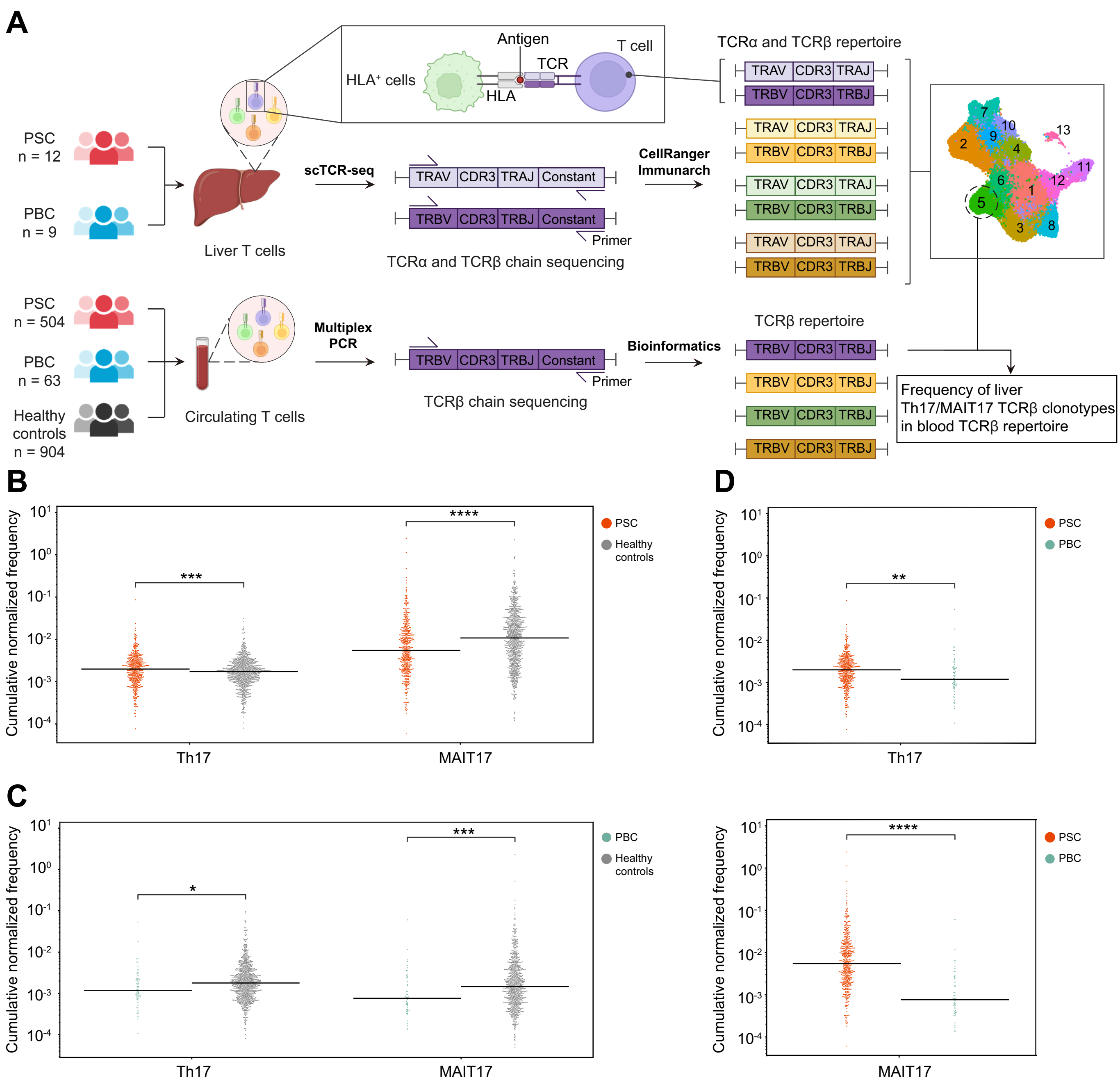

Fig. 4. Identification of PSC and PBC Th17/MAIT17 liver clonotypes in blood of individuals with PSC, PBC, and HC. (A) Workflow for identifying proportions of Th17 and MAIT17 liver TCR clonotypes shared with blood T cell repertoires of patients with PSC (n = 504), PBC (n = 63), and healthy controls (n = 904). (B) Cumulative normalized frequency of PSC liver Th17 and MAIT17 TCR clonotypes found in blood of PSC and healthy controls. (C) Cumulative normalized frequency of PBC liver Th17 and MAIT17 TCR clonotypes found in blood of PBC and healthy controls. (D) Comparison of PSC liver Th17/MAIT17 clonotypes in PSC blood *vs*. PBC liver Th17/MAIT17 clonotypes in PBC blood. Statistical significance was evaluated by Mann-Whitney *U* test in Python using statannotations package, *p ≤0.05, **p ≤0.01, ***p ≤0.001, ****p ≤0.0001. MAIT17, IL-17+ mucosal-associated invariant T cell; ns, non-significant; PBC, primary biliary cholangitis; PSC, primary sclerosing cholangitis.

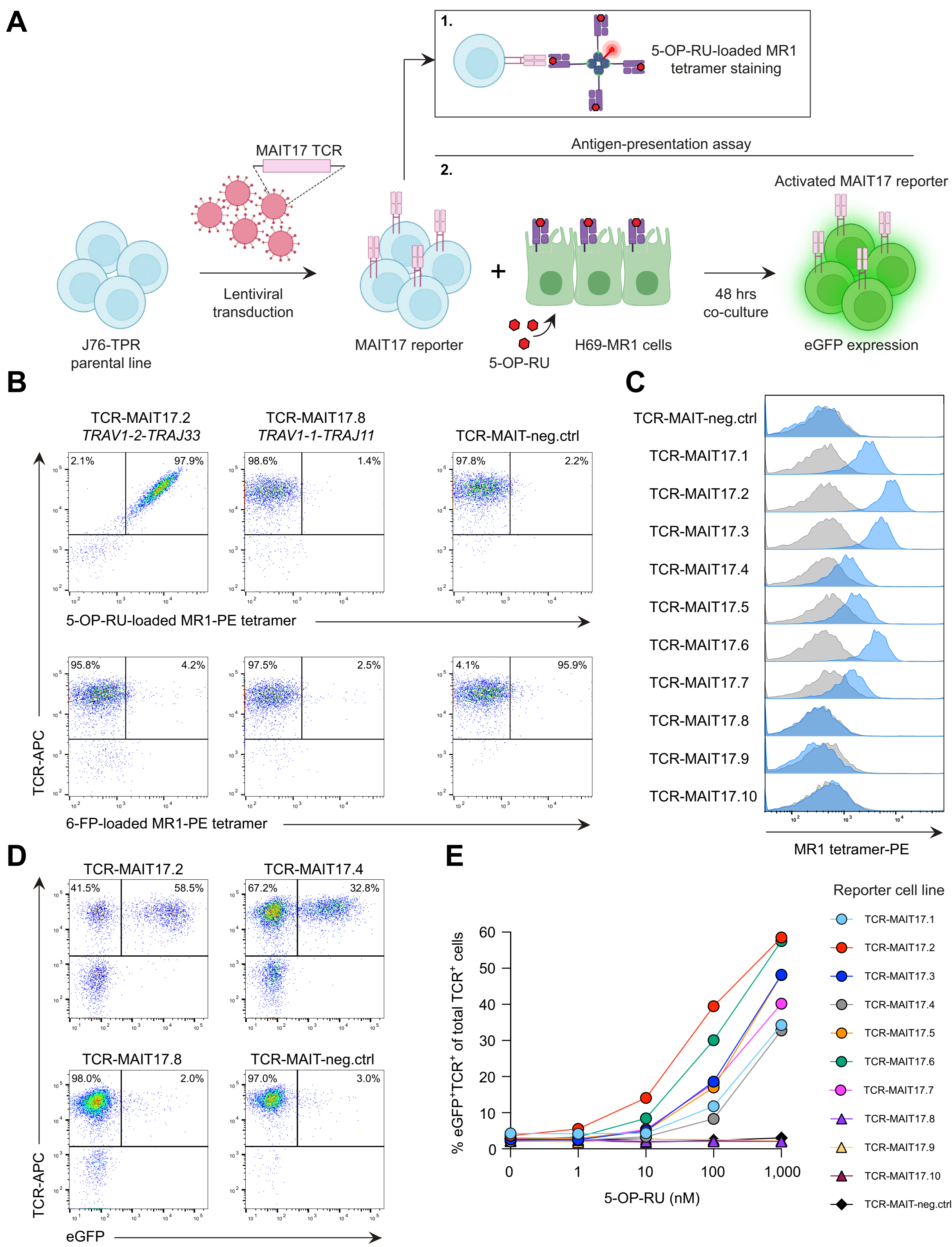

Fig. 5. Expanded MAIT17 TCR clonotypes in PSC livers recognize 5-OP-RU. (A) Generation, tetramer validation, and activation of MAIT17 TCR reporters. (B) 5-OP-RU-MR1 tetramer staining of MAIT17 TCR reporters generated from TCR-seq of liver T cells. Plots show staining of MAIT17 TCR cell lines expressing prototypical MAIT TCR (*TRAV1-2-TRAJ33*), non-prototypical MAIT TCR (*TRAV1-1-TRAJ11*) and non-MAIT TCR (TCR-MAIT-neg.ctrl). (C) 5-OP-RU-MR1 and 6-FP-MR1 (negative) tetramer staining overlay of MAIT17 TCR reporters (n = 10). (D) NFAT-eGFP expression indicates MAIT17 TCR activation by 5-OP-RU-pulsed H69-MR1 cholangiocytes. (E) 5-OP-RU activation of all MAIT17 TCR reporters. 5-OP-RU, 5-(2-oxopropylideneamino)-6-d-ribitylaminouracil; 6-FP, 6-formylpterin; eGFP, enhanced green fluorescent protein; J76-TPR, Jurkat 76 triple parameter reporter; MAIT17, *IL17*$^{+}$ mucosal-associated invariant T cell; MR1, major histocompatibility complex class I-related gene protein; TCR, T cell receptor.

In conclusion, we show using multiple lines of evidence that Th17 and MAIT17 are enriched in livers of individuals with PSC which supports the potential use of targeted T cell therapies for the future treatment of PSC.

Affiliations

[1]*Norwegian PSC Research Center, Department of Transplantation Medicine, Division of Surgery and Specialized Medicine, Oslo University Hospital Rikshospitalet, Oslo, Norway;* [2]*Research Institute of Internal Medicine, Division of Surgery and Specialized Medicine, Oslo University Hospital Rikshospitalet, Oslo, Norway;* [3]*Institute of Clinical Medicine, Faculty of Medicine, University of Oslo, Oslo, Norway;* [4]*Institute of Clinical Molecular Biology, University of Kiel and University Hospital Schleswig-Holstein (UKSH), Kiel, Germany;* [5]*Department of Gastroenterology, Hepatology and Infectious Diseases, Medical Faculty, Heinrich Heine University Düsseldorf, University Hospital Düsseldorf, Düsseldorf, Germany;* [6]*Department of Immunology and Immunotherapy, School of Infection, Inflammation and Immunology, College of Medicine and Health, University of Birmingham, Birmingham, UK;* [7]*NIHR Biomedical Research Centre, Centre for Liver and Gastrointestinal Research, College of Medicine and Health, University of Birmingham, Birmingham, UK;* [8]*Centre for Rare Diseases, European Reference Network on Hepatological Diseases (ERN-RARE-LIVER) Centre, Birmingham, UK;* [9]*Liver Unit, University Hospitals Birmingham National Health Service Foundation Trust Queen Elizabeth, Birmingham, UK;* [10]*Section of Gastroenterology, Department of Transplantation Medicine, Division of Surgery and Specialized Medicine, Oslo University Hospital Rikshospitalet, Oslo, Norway;* [11]*Hybrid Technology Hub, Institute of Basic Medical Sciences, University of Oslo, Oslo, Norway*

Abbreviations

5-OP-RU, 5-(2-oxopropylideneamino)-6-d-ribitylaminouracil; 6-FP, 6-formylpterin; ALD, alcohol-related liver disease; ALP, alkaline phosphatase; ALT, alanine aminotransferase; D, dendritic cell; eGFP, enhanced green fluorescent protein; EM, effector memory; FIB-4, fibrosis-4; H, hepatocyte; HC, healthy controls; IBD, inflammatory bowel disease; IF, immunofluorescence; IL-17+ mucosal-associated invariant T cell; INR, international normalized ratio; J76-TPR, Jurkat 76 triple parameter reporter; LMC, liver mononuclear cells; MAIT, mucosal-associated invariant; MAIT17, *IL17*$^+$ mucosal-associated invariant T cell; MASH, metabolic dysfunction-associated steatohepatitis; MELD-Na, model for end-stage liver disease; MFI, mean fluorescence intensity; MR1, major histocompatibility complex class I-related gene protein; P, plasma cell; PBC, primary biliary cholangitis; PSC, primary sclerosing cholangitis; scRNA-seq, single-cell RNA sequencing; snRNA-seq, single-nuclei RNA seq; T, T cell; T_c, cytotoxic T cell; T_{CM}, central memory T cell; TCR, T cell receptor; TCR-seq, T cell receptor sequencing; T_{EM}, effector memory T cell; T_{FH}-like, follicular-helper like T cell; Th17, T helper type 17; Treg, regulatory T cell; T_{RM}, tissue-resident memory T cell; UMAP, uniform manifold approximation and projection.

Financial support

This work was funded by the Research Council of Norway (325435), National Research Foundation of Korea (RS-2023-00268071) and Norwegian PSC Research Center. LRVB was supported by a Helse Sør-Øst PhD fellowship (340365) and MSJ by the Mildred-Scheel-Stipendienprogramm für Krebsforschung (57584079).

Conflicts of interest

The authors declare no conflicts of interest.

Please refer to the accompanying ICMJE disclosure forms for further details.

Authors' contributions

Designed the study and wrote the manuscript: LRVB, EM, BKC. Performed wet lab experiments: LRVB, BKC. Assisted with bioinformatics and data management: JØ. Provided and reviewed clinical information: MSJ, AGB, DK, TF, YHO. Shared and analyzed blood TCR sequencing data: HE, AF. Assisted with flow cytometry setup and analysis: XJ. Supplied patient samples: YHO, PJT, THK, TF, EM. Coordinated project contributions: EM, BKC. All authors approved the final manuscript.

Data availability

Data are available upon reasonable request from the corresponding author BKC.

Acknowledgements

We thank Professor Peter Steinberger at the Medical University of Vienna for J76-TPR cells, the Norwegian Sequencing Centre Ullevål for RNA sequencing, Umair Majid for human intestine to optimize RORγt IHC staining, Oda Helgesen Ramberg and Jørgen D. Rønneberg for technical support, Merete Tysdahl and Grace Wootton for administrative support, Enya Amundsen-Isaksen and Anna Katharina Frank for discussions regarding lentiviral vector design, and Sissel Åkra for sample retrieval. Figure schematics were created using BioRender (Canada).

Supplementary data

Supplementary data to this article can be found online at https://doi.org/10.1016/j.jhepr.2026.101857.

References

Author names in bold designate shared co-first authorship

[1] Manns MP, Bergquist A, Karlsen TH, et al. Primary sclerosing cholangitis. Nat Rev Dis Primers 2025;11:17.

[2] Karlsen TH, Folseraas T, Thorburn D, et al. Primary sclerosing cholangitis - a comprehensive review. J Hepatol 2017;67:1298–1323.

[3] Jiang X, Karlsen TH. Genetics of primary sclerosing cholangitis and pathophysiological implications. Nat Rev Gastroenterol Hepatol 2017;14:279–295.

[4] Henriksen EK, Jorgensen KK, Kaveh F, et al. Gut and liver T-cells of common clonal origin in primary sclerosing cholangitis-inflammatory bowel disease. J Hepatol 2017;66:116–122.

[5] Chung BK, Henriksen EKK, Jorgensen KK, et al. Gut and liver B cells of common clonal origin in primary sclerosing cholangitis-inflammatory bowel disease. Hepatol Commun 2018;2:956–967.

[6] Zimmer CL, von Seth E, Buggert M, et al. A biliary immune landscape map of primary sclerosing cholangitis reveals a dominant network of neutrophils and tissue-resident T cells. Sci Transl Med 2021;13:eabb3107.

[7] Jiang X, Otterdal K, Chung BK, et al. Cholangiocytes modulate CD100 expression in the liver and facilitate pathogenic T-Helper 17 cell differentiation. Gastroenterology 2024;166:667–669.

[8] **Sebode M**, **Peiseler M**, Franke B, et al. Reduced FOXP3(+) regulatory T cells in patients with primary sclerosing cholangitis are associated with IL2RA gene polymorphisms. J Hepatol 2014;60:1010–1016.

[9] Liaskou E, Jeffery LE, Trivedi PJ, et al. Loss of CD28 expression by liver-infiltrating T cells contributes to pathogenesis of primary sclerosing cholangitis. Gastroenterology 2014;147:221. 32.e227.

[10] **Chung BK**, **Jördens MS**, Øgaard J, et al. Multimodal transcriptomics identifies metallothionein as a novel pathway in primary sclerosing cholangitis. Hepatology 2026;83:1066–1081.

[11] Chung BK, Ogaard J, Reims HM, et al. Spatial transcriptomics identifies enriched gene expression and cell types in human liver fibrosis. Hepatol Commun 2022;6:2538–2550.

[12] Ramachandran P, Matchett KP, Dobie R, et al. Single-cell technologies in hepatology: new insights into liver biology and disease pathogenesis. Nat Rev Gastroenterol Hepatol 2020;17:457–472.

[13] **Poch T**, **Krause J**, Casar C, et al. Single-cell atlas of hepatic T cells reveals expansion of liver-resident naive-like CD4(+) T cells in primary sclerosing cholangitis. J Hepatol 2021;75:414–423.

[14] **Andrews TS**, **Nakib D**, Perciani CT, et al. Single-cell, single-nucleus, and spatial transcriptomics characterization of the immunological landscape in the healthy and PSC human liver. J Hepatol 2024;80:730–743.

[15] ElAbd H, Pesesky M, Innocenti G, et al. T and B cell responses against Epstein-Barr virus in primary sclerosing cholangitis. Nat Med 2025;31:2306–2316.

[17] Nazarov VI, Tsvetkov VO, Popov AA, et al. mmunarch: bioinformatics analysis of T-cell and B-cell immune repertoires. 2023. https://github.com/immunomind/immunarch.

[18] Chen L, Flies DB. Molecular mechanisms of T cell co-stimulation and co-inhibition. Nat Rev Immunol 2013;13:227–242.
[19] Garner LC, Amini A, FitzPatrick MEB, et al. Single-cell analysis of human MAIT cell transcriptional, functional and clonal diversity. Nat Immunol 2023;24:1565–1578.
[20] **Alvisi G**, **Termanini A**, Soldani C, et al. Multimodal single-cell profiling of intrahepatic cholangiocarcinoma defines hyperactivated Tregs as a potential therapeutic target. J Hepatol 2022;77:1359–1372.
[21] Toubal A, Nel I, Lotersztajn S, et al. Mucosal-associated invariant T cells and disease. Nat Rev Immunol 2019;19:643–657.
[22] ElAbd H, Byron R, Woodhouse S, et al. Seq2MAIT: a novel deep learning framework for identifying mucosal associated invariant T (MAIT) cells. bioRxiv 2024. https://doi.org/10.1101/2024.03.12.584395.
[23] **Garner LC**, **Klenerman P**, **Provine NM**. Insights into mucosal-associated invariant T cell biology from studies of invariant natural killer T cells. Front Immunol 2018;9:1478.
[24] **Jeffery HC**, **Hunter S**, **Humphreys EH**, et al. Bidirectional cross-talk between biliary epithelium and Th17 cells promotes local Th17 expansion and bile duct proliferation in biliary liver diseases. J Immunol 2019;203:1151–1159.
[25] **Oo YH**, **Banz V**, Kavanagh D, et al. CXCR3-dependent recruitment and CCR6-mediated positioning of Th-17 cells in the inflamed liver. J Hepatol 2012;57:1044–1051.
[26] **Katt J**, **Schwinge D**, **Schoknecht T**, et al. Increased T helper type 17 response to pathogen stimulation in patients with primary sclerosing cholangitis. Hepatology 2013;58:1084–1093.
[27] **Kunzmann LK**, **Schoknecht T**, Poch T, et al. Monocytes as potential mediators of pathogen-induced T-helper 17 differentiation in patients with primary sclerosing cholangitis (PSC). Hepatology 2020;72:1310–1326.
[28] Nakamoto N, Sasaki N, Aoki R, et al. Gut pathobionts underlie intestinal barrier dysfunction and liver T helper 17 cell immune response in primary sclerosing cholangitis. Nat Microbiol 2019;4:492–503.
[29] **Jeffery HC**, **van Wilgenburg B**, Kurioka A, et al. Biliary epithelium and liver B cells exposed to bacteria activate intrahepatic MAIT cells through MR1. J Hepatol 2016;64:1118–1127.
[30] von Seth E, Zimmer CL, Reuterwall-Hansson M, et al. Primary sclerosing cholangitis leads to dysfunction and loss of MAIT cells. Eur J Immunol 2018;48:1997–2004.
[31] Bottcher K, Rombouts K, Saffioti F, et al. MAIT cells are chronically activated in patients with autoimmune liver disease and promote profibrogenic hepatic stellate cell activation. Hepatology 2018;68:172–186.
[32] Gherardin NA, Souter MN, Koay HF, et al. Human blood MAIT cell subsets defined using MR1 tetramers. Immunol Cell Biol 2018;96:507–525.
[33] **Ivanov II**, **McKenzie BS**, **Zhou L**, et al. The orphan nuclear receptor RORgammat directs the differentiation program of proinflammatory IL-17+ T helper cells. Cell 2006;126:1121–1133.
[34] Shen H, Goodall JC, Hill Gaston JS. Frequency and phenotype of peripheral blood Th17 cells in ankylosing spondylitis and rheumatoid arthritis. Arthritis Rheum 2009;60:1647–1656.
[35] Izadi M, Tahmasebi S, Pustokhina I, et al. Changes in Th17 cells frequency and function after ozone therapy used to treat multiple sclerosis patients. Mult Scler Relat Disord 2020;46:102466.
[36] Zhang L, Yang XQ, Cheng J, et al. Increased Th17 cells are accompanied by FoxP3(+) Treg cell accumulation and correlated with psoriasis disease severity. Clin Immunol 2010;135:108–117.
[37] Huang W, Na L, Fidel PL, et al. Requirement of interleukin-17A for systemic anti-*Candida albicans* host defense in mice. J Infect Dis 2004;190:624–631.
[38] Bonetti L, Horkova V, Grusdat M, et al. A Th17 cell-intrinsic glutathione/mitochondrial-IL-22 axis protects against intestinal inflammation. Cell Metab 2024;36:1726. 44.e1710.
[39] Le Bourhis L, Martin E, Peguillet I, et al. Antimicrobial activity of mucosal-associated invariant T cells. Nat Immunol 2010;11:701–708.
[40] **El Morr Y**, **Furstenheim M**, Mestdagh M, et al. MAIT cells monitor intestinal dysbiosis and contribute to host protection during colitis. Sci Immunol 2024;9:eadi8954.
[41] Hegde P, Weiss E, Paradis V, et al. Mucosal-associated invariant T cells are a profibrogenic immune cell population in the liver. Nat Commun 2018;9:2146.
[42] Haga K, Chiba A, Shibuya T, et al. MAIT cells are activated and accumulated in the inflamed mucosa of ulcerative colitis. J Gastroenterol Hepatol 2016;31:965–972.
[43] **Cho YN**, **Kee SJ**, Kim TJ, et al. Mucosal-associated invariant T cell deficiency in systemic lupus erythematosus. J Immunol 2014;193:3891–3901.
[44] **Bodd M**, **Raki M**, Bergseng E, et al. Direct cloning and tetramer staining to measure the frequency of intestinal gluten-reactive T cells in celiac disease. Eur J Immunol 2013;43:2605–2612.

Keywords: Primary sclerosing cholangitis; Primary biliary cholangitis; Alcohol-related liver disease; Single-cell RNA sequencing; T cell receptor sequencing; IL-17 producing helper T cells; mucosal-associated invariant T cells.
Received 21 August 2025; received in revised form 26 March 2026; accepted 7 April 2026; Available online 17 April 2026

Journal of Hepatology, Volume *8*

Supplemental information

Intrahepatic T cell analysis reveals Th17 and MAIT17 enrichment in primary sclerosing cholangitis

Lisa R.V. Brynjulfsen, Hesham ElAbd, Jonas Øgaard, Markus S. Jördens, Amber G. Bozward, Daniel Kearns, Xiaojun Jiang, Trine Folseraas, Palak J. Trivedi, Ye H. Oo, Andre Franke, Tom H. Karlsen, Espen Melum, and Brian K. Chung

Intrahepatic T-cell analysis reveals Th17 and MAIT17 enrichment in primary sclerosing cholangitis

Lisa R. V. Brynjulfsen, Hesham ElAbd, Jonas Øgaard, Markus S. Jördens, Amber G. Bozward, Daniel Kearns, Xiaojun Jiang, Trine Folseraas, Palak J. Trivedi, Ye H. Oo, Andre Franke, Tom H. Karlsen, Espen Melum, Brian K. Chung

Table of contents

Supplementary materials & methods……………………………………………….………2

Supplementary references…………………………………………………………………..8

Supplementary figures……………………………………………………………………….9

Supplementary tables………………………………………………………………………18

Supplementary Materials & Methods

LMC isolation and cryopreservation

Liver sections (~100 g) were minced into 1-2 mm^3 pieces, washed with PBS and incubated at 37°C for 30 min in digestion medium consisting of RPMI-1640 supplemented with 3% fetal bovine serum (FBS) and collagenase type 1A (VWR International, Radnor, USA). Digested tissue was mechanically homogenized using a Stomacher 400 circulator (Seward, Worthing, UK) and filtered through 60-70 μm nylon strainers with PBS. Filtered suspensions were transferred into 50 ml conical tubes and spun at 600 g for 5 min. Supernatant was discarded and cell pellets were washed 3-4 more times in PBS. Washed pellets were resuspended in PBS and layered over Lympholyte-H (Cedarlane, Burlington, Canada) and spun at 600 g for 20 min to isolate LMCs by density centrifugation. Isolated LMCs were resuspended in Cryostor-10 (Sigma-Aldrich, St.Louis, USA) at 8-16 million cells per vial and stored in liquid nitrogen.[1]

T cell enrichment from LMCs

Cryopreserved LMCs were thawed and washed in warm RPMI 1640 with GlutaMAX and HEPES (Thermo Fisher Scientific, Waltham, USA) supplemented with 20% FBS (Sigma-Aldrich). Washed LMCs were resuspended in PBS (Lonza, Basel, Switzerland) supplemented with 2% FBS and 1 mM EDTA (Thermo Fisher Scientific), treated with 100 μg/ml DNase I (QIAGEN, Hilden, Germany) for 15 min and filtered through 30 μm strainers. T cells were enriched from LMCs using negative-selection magnetic bead kits following the manufacture's recommendations (STEMCELL Technologies, Vancouver, Canada). To exclude dead cells, enriched T cells were stained with 7-aminoactinomycin D (7-AAD, 1:333 dilution; Thermo Fisher Scientific) or Zombie NIR (1:333 dilution; BioLegend, San Diego, USA) for 5 min and sorted using a FACSMelody flow cytometer (BD Biosciences, Franklin Lakes, USA). Viable T cell enrichments were filtered through 30 μm strainers and enumerated using a Countess II Automated Cell Counter (Thermo Fisher Scientific) for scRNA-seq.

Preprocessing, clustering, differential gene expression analysis and cluster annotation

Sequencing reads were demultiplexed and aligned to human genome GRCh38-2020-A using the Cell Ranger v7.1.0 multi pipeline (10x Genomics).[2] Filtered outputs were loaded into R v4.3.2 for preprocessing with Seurat v.5.0.1.[3] Features detected in fewer than 3 cells and cells with fewer than 200 detected features were excluded. Cell doublets and empty droplets outside the 1.5 interquartile range (IQR) for total unique molecular identifier (UMI) counts per cell and unique features per cell were excluded. Further, dead cells with mitochondrial gene count percentage per cell above the upper fence of the 1.5 IQR were also excluded. Preprocessed samples were merged into a single SeuratObject and normalized using NormalizeData. The top 2000 variable features were identified using FindVariableFeatures and scaled using ScaleData. Principal component analysis (PCA) was performed using RunPCA and batch effects were adjusted using CCA integration.[4, 5]

To define cell clusters, FindNeighbors was performed using the first 30 principal components (PCs) followed by FindClusters with a resolution parameter of 1.3. DEG analysis for each cluster was performed using FindAllMarkers to obtain marker genes and all reported cluster marker genes reached statistical significance ($P<0.01$, Wilcoxon Rank Sum test) and ordered by ascending P-value. To exclude non-T cells, only cells with normalized counts of *CD3D* > 0,

CD68 < 1, *CD79A* < 2, *CD79B* < 2, and *ALB* < 5 were analyzed. Reclustering of filtered T cells was repeated using the first 21 PCs and resolution setting of 0.5. Marker DEGs for each T cell cluster were identified using FindAllMarkers and reached a $\log_2$ fold-change > 0.5, expressed in > 25% of the cells in the cluster and had an adjusted P-value < 0.05. Heatmaps showing the top 40 DEGs for each cluster in the total T cell UMAP and the top 25 DEGs for the Th17/MAIT17 were generated using DoHeatmap and downsampled to display a maximum of 1000 cells per cluster for the total T cell UMAP.

Using canonical lineage markers,[6-10] all 13 T cell clusters in the total T cell UMAP were annotated into discrete phenotypes. Cluster 1 ($CD4^+$ $T_{EM}1$) was the most abundant in 14 of 24 livers (18.5 ± 8.0%) and expressed the effector memory (EM) markers *TNF*, *IFNG* and *CXCR3*. Of the four remaining $CD4^+$ subsets, cluster 3 ($CD4^+$ T_{CM}) was the second largest and featured the central memory (CM) markers *CCR7* and *IL7R*, while cluster 6 ($CD4^+$ $T_{EM}2$) shared EM markers with $CD4^+$ $T_{EM}1$ (*CD40LG*, *IL7R*, *CD4*) but elevated expression of *KLRB1*, *STAT1*, and *CXCR6*. Cluster 11 (Tregs) featured the conventional Treg genes *FOXP3*, *CTLA4*, *CD27*, *IL2RA* and cluster 12 expressed the follicular-helper T cell (T_{FH}) markers *CTLA-4*, *PDCD1*, *TOX2*, and *ICOS*.

Of the five $CD8^+$ subsets, cluster 2 (innate-like $CD8^+$ T_c) was the most abundant (14.2 ± 14.2%) and featured co-expression of the innate leukocyte marker *FCGR3A* and the cytotoxic T cell (T_c) genes *FGFBP2*, *GZMB* and *PRF1*. Cluster 4 ($CD8^+$ T_{RM}) was classified as $CD8^+$ tissue-resident memory (RM) based on *ITGAE* and *CXCR6* expression and cluster 7 (innate-like γδ T cells) featured both *FCGR3A* and the TCR γδ-genes *TRDC* and *TRDV1*. Cluster 9 ($CD8^+$ T_c) expressed the cytotoxic markers *GZMK*, *GZMA*, *NKG7* but lacked innate leukocyte markers. Cluster 10 ($CD8^+$ T_{EM}/γδ) expressed low levels of *CCR7* and cytotoxic markers characteristic of $CD8^+$ effector memory T cells and was also positive for the TCR γδ-genes *TRDC* and *TRDV1*.

Three of 13 subsets contained a mixture of both $CD4^+$ and $CD8^+$ cells with cluster 5 constituting the largest (8.7 ± 5.5%) featuring *CCR6*, *RORC*, *IL23R* and *KLRB1* indicative of both Th17 and MAIT17 cells. Cluster 8 was annotated as naïve based on the expression of *CCR7*, *SELL*, and *TCF7* while cluster 13 (cycling) was the smallest T cell subset overall (0.6 ± 0.4%) and distinguished by heightened expression of the cell cycle markers *MKI67* and *RRM2*.

To partition the Th17/MAIT17 cluster, Th17/MAIT17 cells was subsetted from total T cells and reclustered using the same methods described for total T cells with PCA set on *TRAV1-2*, *CD8A*, *CD8B*, *NKG7*, *NCR3*, *GZMK*, *CTSW*, *CD4*, *GPR183*, *MAP3K4*, *CD40LG*, *SAMSN1* and *AHR*.

Data integration was performed using CCA integration with the k.weight parameter set to 41. The first 10 PCs were used for clustering Th17/MAIT17 cells. Resolution was set to 0.2. UMAPs were generated with RunUMAP using the first 10 PCs and minimum distance of 0.8.

Clusters annotated as CD4-positive, alpha-beta T cell, CD8-positive, alpha-beta T cell and T cell from Andrews and Nakib *et al*. (https://cellxgene.cziscience.com/collections/0c8a364b-97b5-4cc8-a593-23c38c6f0ac5) were merged into a single SeuratObject with total T cells from our study. NormalizeData, FindVariableFeatures, ScaleData, RunPCA, CCA integration with

k.weight set to 42. Clustering and generation of UMAP on merged data was performed using the first 25 PCs and with the resolution parameter set to 0.6.

Analysis of liver and blood TCR overlap and blood MAIT cell frequencies
DNA was extracted from EDTA-blood of individuals with PSC (n=504), PBC (n=63) and age and sex-matched controls (n=904) using the Qiagen DNeasy Blood Extraction Kit (QIAGEN, Hilden, Germany). Isolated DNA was amplified for TCR β-chain repertoires using the immuneSEQ assay (Adaptive Biotechnologies, Seattle, USA) as previously reported.[11] Briefly, after library preparation, sequencing and clonotype identification, non-productive clonotypes containing a stop-codon or a frameshift were excluded. Different V(D)J recombination sequences encoding the same TCR β-chain sequence at the protein level were combined by summing their expansion thus a blood TCR clonotype was defined herein as a unique recombination of V and J genes and a unique amino acid CDR3 sequence.

TCR and MR1 DNA constructs and lentiviral vectors
Paired TCR αβ-chain variable domain RNA sequences from a subset of expanded PSC liver MAIT17 clonotypes identified by TCR-seq (n=10, Supplementary Table 3) were cloned into DNA constructs for lentiviral transduction into J76-triple parameter reporters (J76-TPR) that lack endogenous TCR and express enhanced green fluorescent protein (eGFP) upon TCR-mediated NFAT activation.[12, 13] For each TCR construct, a MAIT17 TCR β-chain variable domain sequence from TCR-seq was followed by sequences for a murine TCR β-chain constant region, cysteine bridge, P2A self-cleaving peptide, MAIT17 TCR α-chain variable domain from TCR-seq, murine TCR α-chain constant region and cysteine bridge.[14] To generate the MAIT17 TCR negative control, protein sequences for the TCR αβ-variable domains know to recognize a HLA-DRB1*13:01-restricted p53 mutant (4285-TCR1)[15] were reverse translated and codon-optimized for expression in human cells. Human MR1 sequence (GenBank: NM_001531.2) was cloned into DNA constructs for lentiviral transduction of H69 cholangiocytes.[16] TCR- and MR1-encoding pLV-Puro-EF1α lentiviral vectors were synthesized by VectorBuilder (Chicago, Illinois, USA) and detailed in Supplementary Figure 1A and 1B.

Flow cytometry
Cryopreserved LMCs were thawed in RPMI 1640 with GlutaMAX and HEPES (Thermo Fisher Scientific) containing 20% FBS (Sigma-Aldrich), filtered through 30 μm strainers and incubated at 10^6 cells/ml in RPMI containing 10% FBS for 3.5 hr at 37°C in a humidified 5% CO_2 incubator. Following incubation, cells were treated with FcR blocking reagent (1:5, Miltenyi Biotec, Bergisch Gladbach, Germany) for 10 min at 4°C in PBS (Lonza), washed and then stained with BV421 anti-human CCR6 (1:100, clone G034E3, BioLegend) and BV786 anti-human CCR7 (1:50, clone 3D12, Thermo Fisher Scientific) for 30 min at 37°C in Brilliant stain buffer (1:2, Thermo Fisher Scientific) diluted with RPMI and 10% FBS. Cells were then stained for 30 min at 4°C for extracellular markers using the following antibodies diluted 1:50 in a mixture of PBS, Brilliant stain buffer (1:2, Thermo Fisher Scientific) and CellBlox Plus blocking buffer (1:20, Thermo Fisher Scientific): Alexa Fluor 488 anti-human CD3 (clone OKT3, Thermo Fisher Scientific), PE/Fire 810 anti-human TCR γ/δ (clone B1, BioLegend), RB780 anti-human CD161 (clone DX12, BD Biosciences), BV480 anti-human TCR Vα7.2 (clone OF-5A12, BD Biosciences), PerCP/Cyanine5.5 anti-human CD8a (clone RPA-T8, BioLegend), BUV 496 anti-human CD4 (clone RPA-T4, Thermo Fisher Scientific), BUV395 anti-human CD45RA (clone

HI100, Thermo Fisher Scientific), NovaFluor Red 700 anti-human CD25 (clone BC96, Thermo Fisher Scientific), BV650 anti-human PD-1 (clone J105, Thermo Fisher Scientific), Super Bright 600 anti-human ICOS (clone ISA-3, Thermo Fisher Scientific) and LIVE/DEAD Fixable Near-IR Dead Cell Stain (1:200, Thermo Fisher Scientific). LMCs were washed, fixed, permeabilized and re-stained for intracellular markers using the Foxp3 intracellular staining kit (Thermo Fisher Scientific) following the manufacturer´s instructions. Intracellular staining was performed with PE-eFluor 610 anti-human RORγt (1:200, clone AFKJS-9, Thermo Fisher Scientific), PE-Cy7 anti-human FOXP3 (1:50, clone PCH101, Thermo Fisher Scientific), BUV737 anti-human CTLA-4 (1:50, clone 14D3, Thermo Fisher Scientific), PE anti-human FGFBP2 (1:50, clone TDA3, BioLegend), and BV711 anti-human Bcl-6 (1:50, clone K112-91, BD Biosciences) in Brilliant stain buffer (1:2 diluted in 1x permeabilization buffer) for 30 min at room temperature. LMCs were washed in permeabilization buffer, resuspended in PBS and filtered using 30 μm strainers prior for data acquisition on a ID7000 Spectral Cell Analyzer (Sony Biotechnology, San Jose, USA) equipped with five lasers (355 nm, 405 nm, 488 nm, 561 nm, 637 nm). Data was unmixed using Sony ID7000 software v2.0.2.17121 and analyzed with FlowJo v10.10.0 (BD Life Sciences, Franklin Lakes, New Jersey, USA). Fluorescence minus one (FMO) controls were used to determine positive stainings and gating for MAIT17 and Th17 cells is shown in Supplementary Figure 7.

To determine the reactivity of TCR-transduced J76-TPR cell lines to 5-OP-RU, approximately 100,000 cells were stained with PE-conjugated human MR1:5-OP-RU tetramer (1:400) or MR1:6-formylpterin (6-FP, 1:400) control tetramer from the NIH Tetramer Core (Emory University, Atlanta, USA) for 30 min at 4°C. Cells were washed and stained with anti-mouse TCR β-chain-APC (1:50, clone H57-597, BioLegend). Dead cells were excluded using Zombie Violet (1:200, BioLegend). MR1 expression on H69-MR1 cells was measured using anti-human MR1-APC (1:50, clone 26.5, BioLegend). TCR reporters and H69-MR1s were analyzed by flow cytometry data using a LSRFortessa flow cytometer (BD Biosciences) and FlowJo v10.10.0 (BD Life Sciences). All stainings were performed on ice in PBS (Lonza). Gating strategy for MR1 expression on H69-MR1 cells in shown in Supplementary Figure 3A and 3B, while gating strategy for tetramer staining is detailed in Supplementary Figure 2A.

5-OP-RU induced activation of MAIT17 reporter cell lines

On day 1, H69-MR1 cells were seeded at 1 x 10^4 per well in a 96-well flat-bottom plate and incubated overnight in a 37°C humidified 5% CO_2 incubator. On day 2, MAIT17 TCR-transduced J76-TPR cell lines (TCR-MAIT) were added at 5 x 10^4 per well in H69 media supplemented with the indicated concentrations of 5-OP-RU and incubated for 48 hrs in a 37°C humidified 5% CO_2 incubator. J76-TPR cells expressing a non-MAIT17 TCR (4285-TCR1) were cultured with 5-OP-RU-loaded H69-MR1 cells as a negative control. Parental J76-TPR cells cultured with 1000 nM 5-OP-RU without H69 cells also served as negative controls. 5-OP-RU was prepared as by incubating 5-amino-6-D-ribitylaminouracil (5-A-RU, MuseChem, Paoli, Pennsylvania, USA) with methylglyoxal (Sigma-Aldrich) at a 1:25 ratio for 15 minutes at room temperature as previously described.[17] NFAT-eGFP expression used to detect 5-OP-RU activation by MAIT17-TCR reporters was measured using LSRFortessa flow cytometer (BD Biosciences) and analyzed in FlowJo v10.10.0 (BD Life Sciences). Gating strategy for identifying activated TCR-transduced reporter cell lines is shown in Supplementary Figure 2B.

Lentiviral transduction and culture of J76-TPR and H69 cell lines

J76-TPR cells cultured in RPMI 1640 with GlutaMAX and HEPES (Thermo Fisher Scientific) supplemented with 10% fetal bovine serum (FBS; Sigma-Aldrich) and penicillin-streptomycin (1:100, Thermo Fisher Scientific) were infected with TCR-encoding pLV-Puro-EF1α lentiviral vectors at a multiplicity of infection (MOI) of 5 using 8.0 μg/ml polybrene (VectorBuilder). Cells and vectors were spun at 800 g for 30 min at room temperature and maintained in a 37°C humidified 5% CO_2 incubator. Three days post-infection, TCR-transduced J76-TPR cells were selected using 2.5 μg/ml puromycin (Applied Biological Materials, Richmond, Canada) for 48 hr. To generate MR1-transduced H69 cells (H69-MR1), H69 cholangiocytes cultured to 50% confluency in H69-specific supplemented medium [16] were infected overnight with MR1-encoding pLV-Puro-EF1α lentiviral vectors at an MOI of 25 with 8.0 μg/ml polybrene (VectorBuilder) in a 37°C humidified 5% CO_2 incubator. Three days post-infection, H69-MR1s were selected with 3 μg/ml puromycin (Applied Biological Materials) for 48 hr. Following selection, all transduced cell lines were cultured in non-selective media and maintained in a 37°C humidified 5% CO_2 incubator for experiments.

Immunohistochemistry (IHC), immunofluorescence (IF) and staining quantification

Formalin-fixed paraffin-embedded 5 μm liver sections were deparaffinized in histoclear (Histolab Products AB, Askim, Sweden) for 10, 8 and 5 min and rehydrated in series of decreasing ethanol solutions (100% 2x for 1 min, 96% for 1 min, 80% for 1 min and 50% for 1 min) and then stored in distilled water. Antigen retrieval was performed in Envision FLEX Target Retrieval pH 6.1 solution (Agilent Technologies, Santa Clara, USA) for 20 min at 95°C. Sections were left cooling in the machine for 15 min, transferred to the bench top for another 15 min and then placed in PBS. Sections used for RORγt IHC detection and IF staining underwent nuclear permeabilization with 0.1% Triton X-100 (Sigma-Aldrich) in PBS (Santa Cruz Biotechnology, Dallas, USA) for 10 min followed by three washes with 0.1% Tween-20 in PBS (PBST). RORγt and IF sections were the treated with 3% hydrogen peroxide (Merck KGaA, Darmstadt, Germany) while sections used for CD3 IHC staining were treated with 0.3% hydrogen peroxide in PBS for 20 min. All sections were then washed three times with PBST and then blocked with 2.5% normal goat serum or 2.5% normal horse serum (Vector Laboratories, Newark, USA) for 20 min. Sections were incubated overnight at 4°C with mouse anti-human RORγt (1:250, clone 6F3.1, Sigma-Aldrich) or rabbit anti-CD3 (1:500, clone SP162, Abcam, Cambridge, UK) in PBST containing 0.1% bovine serum album (Sigma-Aldrich). Mouse IgG2a antibody (clone: MOPC-173, BioLegend) and rabbit IgG (clone: EPR25A, Abcam) were used as isotype controls.

After overnight incubation, sections for IHC washed 3 times in PBST and stained with ImmPRESS HRP-conjugated goat anti-mouse IgG for 30 min or ImmPRESS HRP-conjugated horse anti-rabbit IgG (Vector Laboratories) for 20 min. After three final washes in PBST, sections used for IHC RORγt detection were stained with DAB (Vector Laboratories) for 3 min while sections used for CD3 detection were stained with DAB for 4 min. Sections were then stored in water for 5 min, counterstained with hematoxylin (Vector Laboratories), dehydrated in series of increasing ethanol solutions (50% for 1 min, 80% for 1 min, 96% for 1 min and 100% for 1 min), washed 3x in histoclear for 1 min and mounted using Eukitt Quick-hardening mounting medium (Sigma-Aldrich). All steps were performed at room temperature unless stated

otherwise. Annotation of positive and negative cells was performed in QuPath v0.5.[18] using the train object classifier set to random trees. While viewing the image on live update, the classifier was trained by manually annotating 10-20 positive and negative cells for CD3 and 20-40 positive and negative cells for RORγt. After automated classification, CD3 and RORγt positive staining was evaluated and adjusted. Percentage of $CD3^+$ cells was calculated by dividing the number of $CD3^+$ cells on the total number of cells detected within the tissue analyzed for CD3. The percentage of RORγt^+ T cells was calculated by dividing the number of RORγt^+ cells on the number of $CD3^+$ cells quantified within the same tissue area.

For IF staining, sections incubated overnight with primary antibodies were washed 3 times in PBST and stained for 30 min with Alexa Fluor 555 donkey anti-mouse IgG (1:1000, Thermo Fisher Scientific) and Alexa Fluor 647 goat anti-rabbit IgG (1:1000, Thermo Fisher Scientific) in PBST containing 0.1% bovine serum album. After washing three times with PBST, IF sections were mounted using EverBrite TrueBlack Hardset Mounting Medium with DAPI (Biotium, Fremont, USA). All steps were performed at room temperature unless stated otherwise. Slides were imaged using a Nikon Eclipse E400 Microscope equipped with a Nikon camera DS-Fi3 (Minato, Japan) and merged using Fiji ImageJ v2.16.0/1.54p[19]

Supplementary References

[1] Chung BK, Guevel BT, Reynolds GM, et al. Phenotyping and auto-antibody production by liver-infiltrating B cells in primary sclerosing cholangitis and primary biliary cholangitis. J Autoimmun. 2017;77:45-54.
[2] Zheng GX, Terry JM, Belgrader P, et al. Massively parallel digital transcriptional profiling of single cells. Nat Commun. 2017;8:14049.
[3] Hao Y, Stuart T, Kowalski MH, et al. Dictionary learning for integrative, multimodal and scalable single-cell analysis. Nat Biotechnol. 2024;42:293-304.
[4] **Stuart T, Butler A,** Hoffman P, et al. Comprehensive Integration of Single-Cell Data. Cell. 2019;177:1888-1902 e1821.
[5] Butler A, Hoffman P, Smibert P, et al. Integrating single-cell transcriptomic data across different conditions, technologies, and species. Nat Biotechnol. 2018;36:411-420.
[6] **Poch T, Krause J,** Casar C, et al. Single-cell atlas of hepatic T cells reveals expansion of liver-resident naive-like CD4(+) T cells in primary sclerosing cholangitis. J Hepatol. 2021;75:414-423.
[7] Xu W, Zhao X, Wang X, et al. The Transcription Factor Tox2 Drives T Follicular Helper Cell Development via Regulating Chromatin Accessibility. Immunity. 2019;51:826-839 e825.
[8] Andreatta M, Corria-Osorio J, Muller S, et al. Interpretation of T cell states from single-cell transcriptomics data using reference atlases. Nat Commun. 2021;12:2965.
[9] Ramachandran P, Dobie R, Wilson-Kanamori JR, et al. Resolving the fibrotic niche of human liver cirrhosis at single-cell level. Nature. 2019;575:512-518.
[10] **Alvisi G, Termanini A,** Soldani C, et al. Multimodal single-cell profiling of intrahepatic cholangiocarcinoma defines hyperactivated Tregs as a potential therapeutic target. J Hepatol. 2022;77:1359-1372.
[11] ElAbd H, Pesesky M, Innocenti G, et al. T and B cell responses against Epstein-Barr virus in primary sclerosing cholangitis. Nat Med. 2025.
[12] Muller TR, Schuler C, Hammel M, et al. A T-cell reporter platform for high-throughput and reliable investigation of TCR function and biology. Clin Transl Immunology. 2020;9:e1216.
[13] Jutz S, Leitner J, Schmetterer K, et al. Assessment of costimulation and coinhibition in a triple parameter T cell reporter line: Simultaneous measurement of NF-kappaB, NFAT and AP-1. J Immunol Methods. 2016;430:10-20.
[14] Muller TR, Jarosch S, Hammel M, et al. Targeted T cell receptor gene editing provides predictable T cell product function for immunotherapy. Cell Rep Med. 2021;2:100374.
[15] Malekzadeh P, Pasetto A, Robbins PF, et al. Neoantigen screening identifies broad TP53 mutant immunogenicity in patients with epithelial cancers. J Clin Invest. 2019;129:1109-1114.
[16] Grubman SA, Perrone RD, Lee DW, et al. Regulation of intracellular pH by immortalized human intrahepatic biliary epithelial cell lines. Am J Physiol. 1994;266:G1060-1070.
[17] **Valestrand L, Zheng F,** Hansen SH, et al. Bile from Patients with Primary Sclerosing Cholangitis Contains Mucosal-Associated Invariant T-Cell Antigens. Am J Pathol. 2022;192:629-641.
[18] Bankhead P, Loughrey MB, Fernandez JA, et al. QuPath: Open source software for digital pathology image analysis. Sci Rep. 2017;7:16878.
[19] Schindelin J, Arganda-Carreras I, Frise E, et al. Fiji: an open-source platform for biological-image analysis. Nat Methods. 2012;9:676-682.
[20] **Andrews TS, Nakib D,** Perciani CT, et al. Single-cell, single-nucleus, and spatial transcriptomics characterization of the immunological landscape in the healthy and PSC human liver. J Hepatol. 2024;80:730-743.

Supplementary Figures

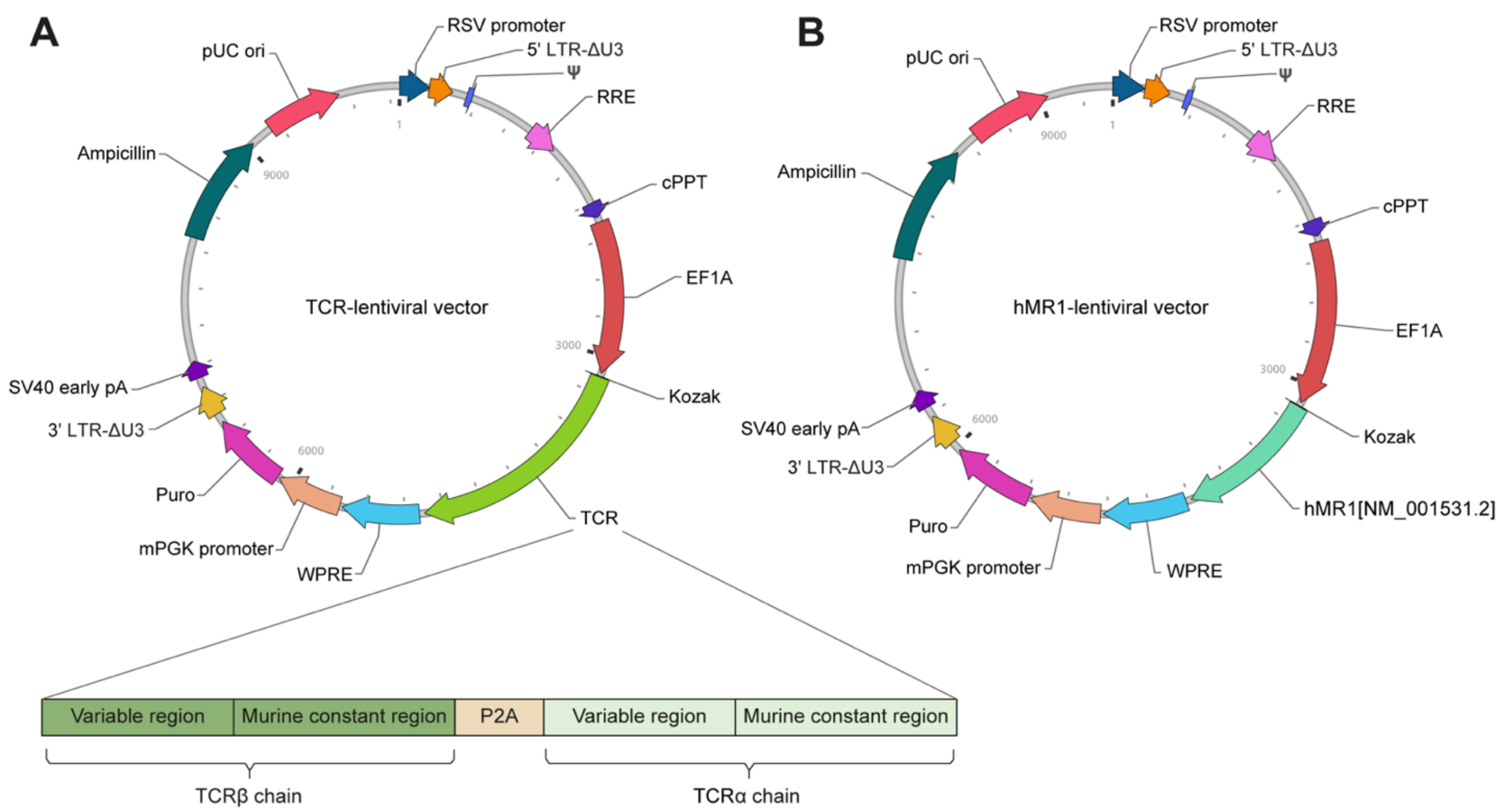

Supplementary Figure 1. Detailed overview of lentiviral TCR and MR1 expression vectors used to generate MAIT17 TCR reporters and H69-MR1 cell line. (A) Representative lentiviral vector used to transduce TCR expression into J76-TPR cell lines. (B) Lentiviral vector used to drive human MR1 overexpression in H69 cholangiocytes. hMR1, human major histocompatibility complex class I-related gene protein; P2A, peptide 2A sequence.

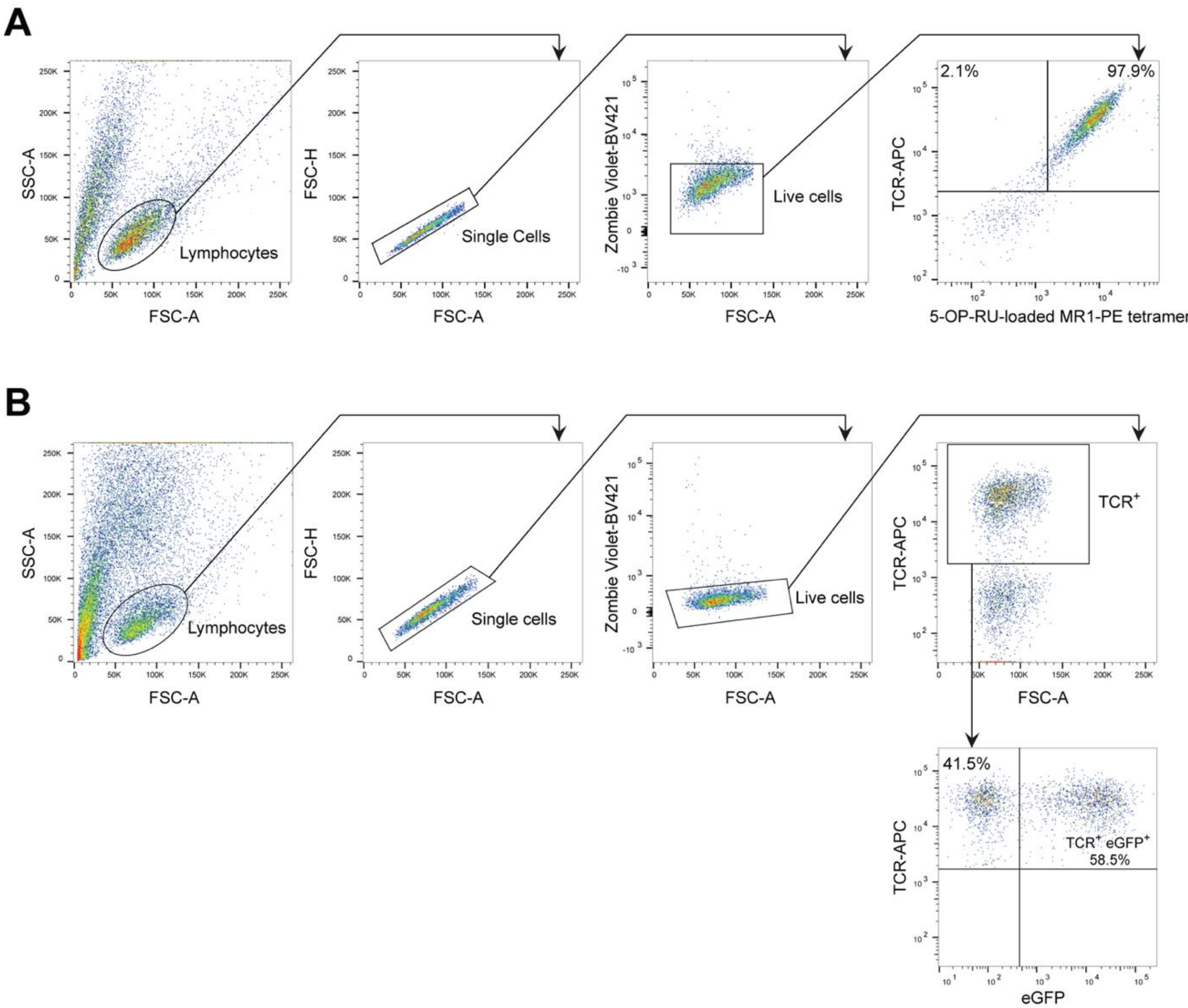

Supplementary Figure 2. Gating strategy for 5-OP-RU-MR1 tetramer staining and activation of TCR reporter cell lines by flow cytometry. (A) TCR-transduced cell lines were gated for lymphocyte morphology and singlets using forward (FSC) and side scatter (SSC). Lymphocyte singlets were gated for viability (Zombie Violet$^-$) and TCR and MR1-tetramer staining. TCR$^+$MR1-tetramer$^+$ cells were categorized as prototypical MAIT cells. Gated frequencies show the percentage of MR1-tetramer$^+$ cells as a fraction of total TCR$^+$ cells. (B) TCR-transduced reporter lines were analyzed for TCR activation by gating on lymphocyte morphology, singlets, live cells (Zombie Violet$^-$) and TCR expression. Gated frequencies show the percentage of TCR$^+$eGFP$^+$ cells as a fraction of total TCR$^+$ cells.

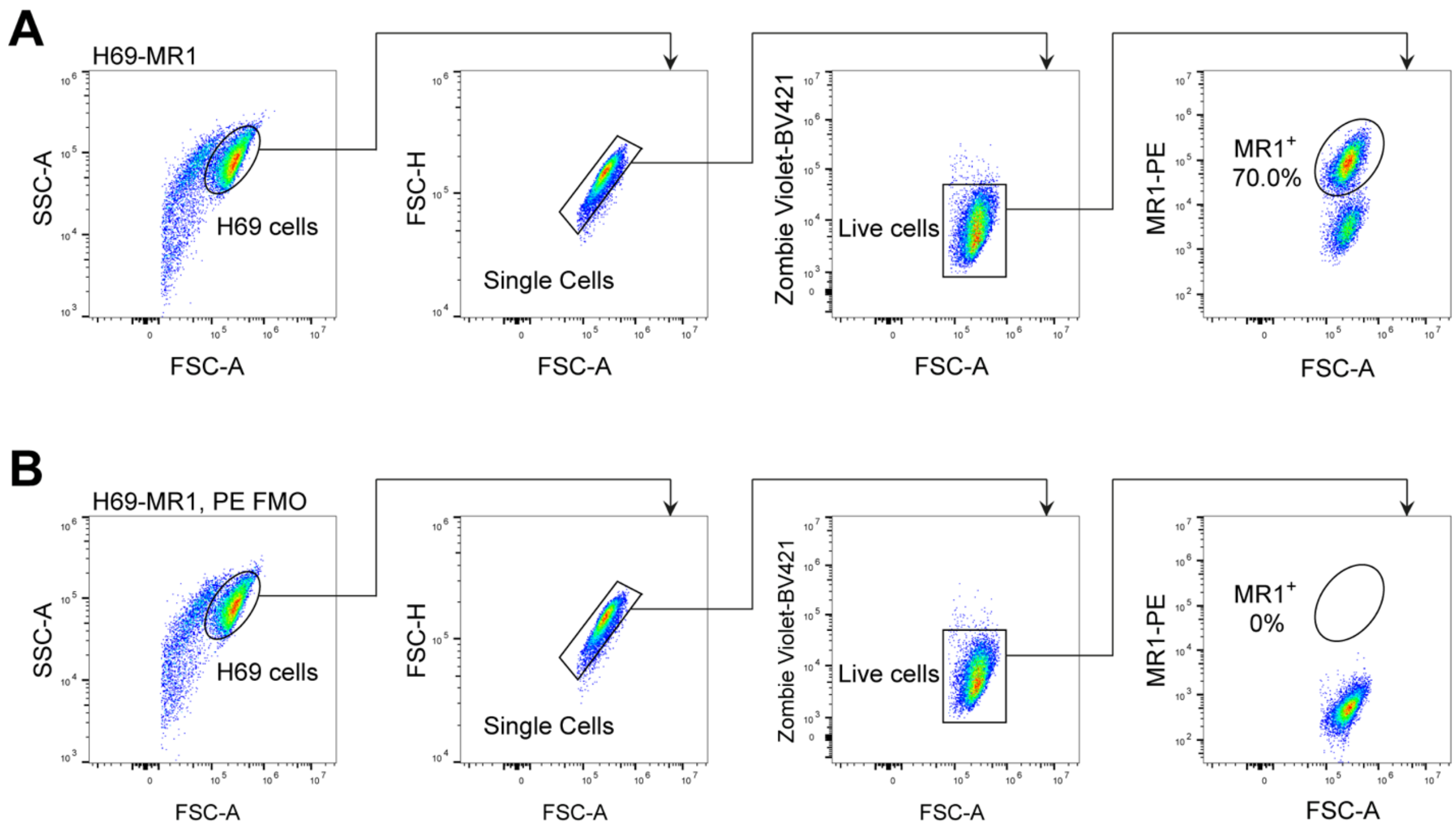

Supplementary Figure 3. MR1 expression on H69-MR1 cholangiocyte cells by flow cytometry. (A) MR1-H69 cells were analyzed for MR1 expression by gating for H69 morphology, singlets using forward (FSC) and side scatter (SSC), live cells (Zombie Violet$^-$), and MR1 staining. (B) FMO control for MR1 staining to assist with the gating of MR1$^+$ cells. H69-MR1 cells were gated on H69 morphology, singlets, live cells (Zombie Violet$^-$), and MR1 staining. FMO, fluorescence minus one; MR1, major histocompatibility complex class I-related gene protein.

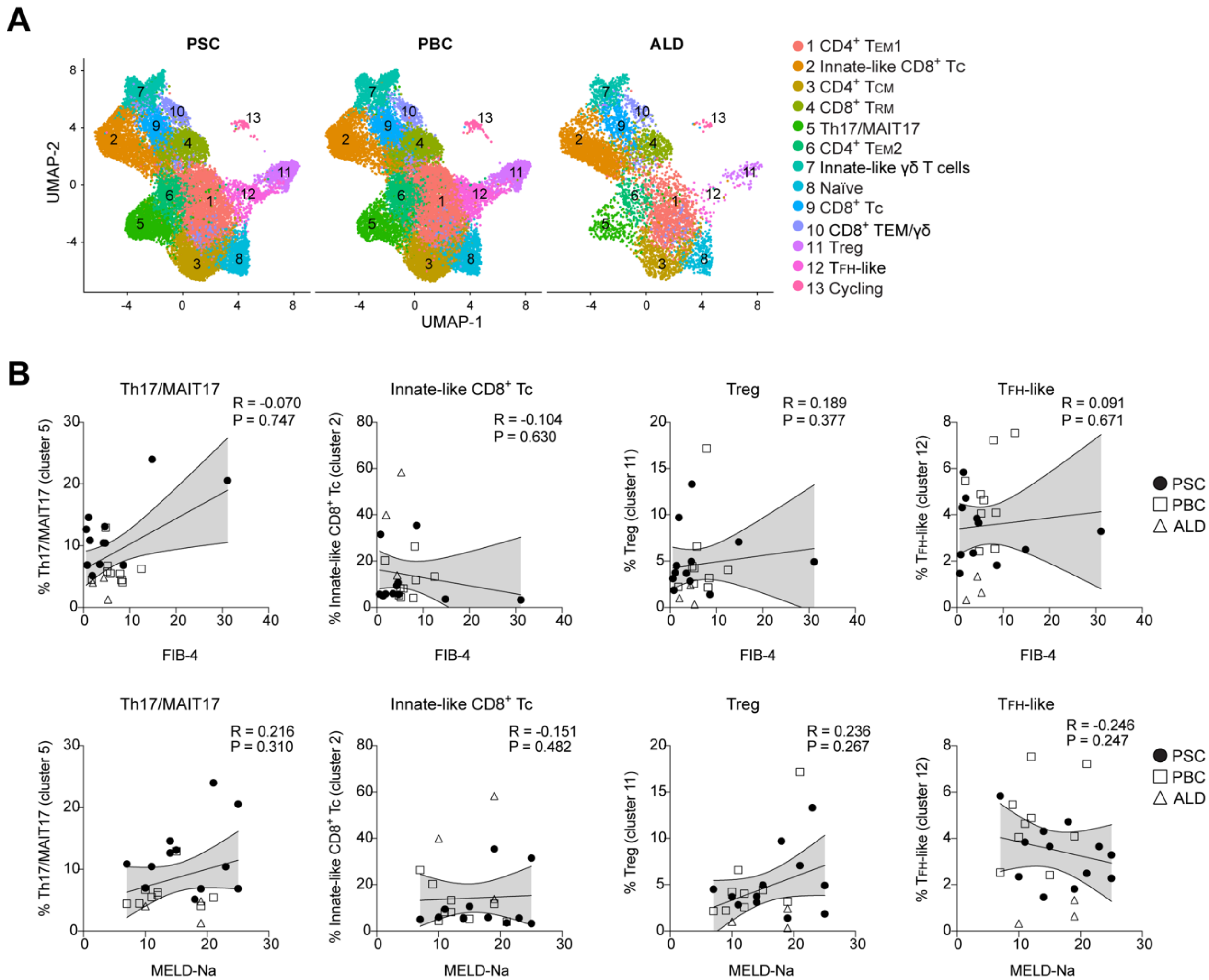

Supplementary Figure 4. Total T cell scRNA-seq UMAP split by disease type and correlation of FIB-4 and MELD-Na with T cell proportions. (A) UMAP showing PSC (n=12, 16,641 cells), PBC (n=9, 18,426 cells), and ALD livers (n=3, 5,830 cells) analyzed by scRNA-seq. (B) Correlation analysis of FIB-4 and MELD-Na with proportions of liver T cell subsets differentially-abundant in PSC, PBC and ALD measured by scRNA-seq (i.e. Th17/MAIT17, innate-like CD8^{+} cytotoxic T_c, Treg, and T_{FH}-like). Statistical significance for correlation analyses evaluated using Spearman.

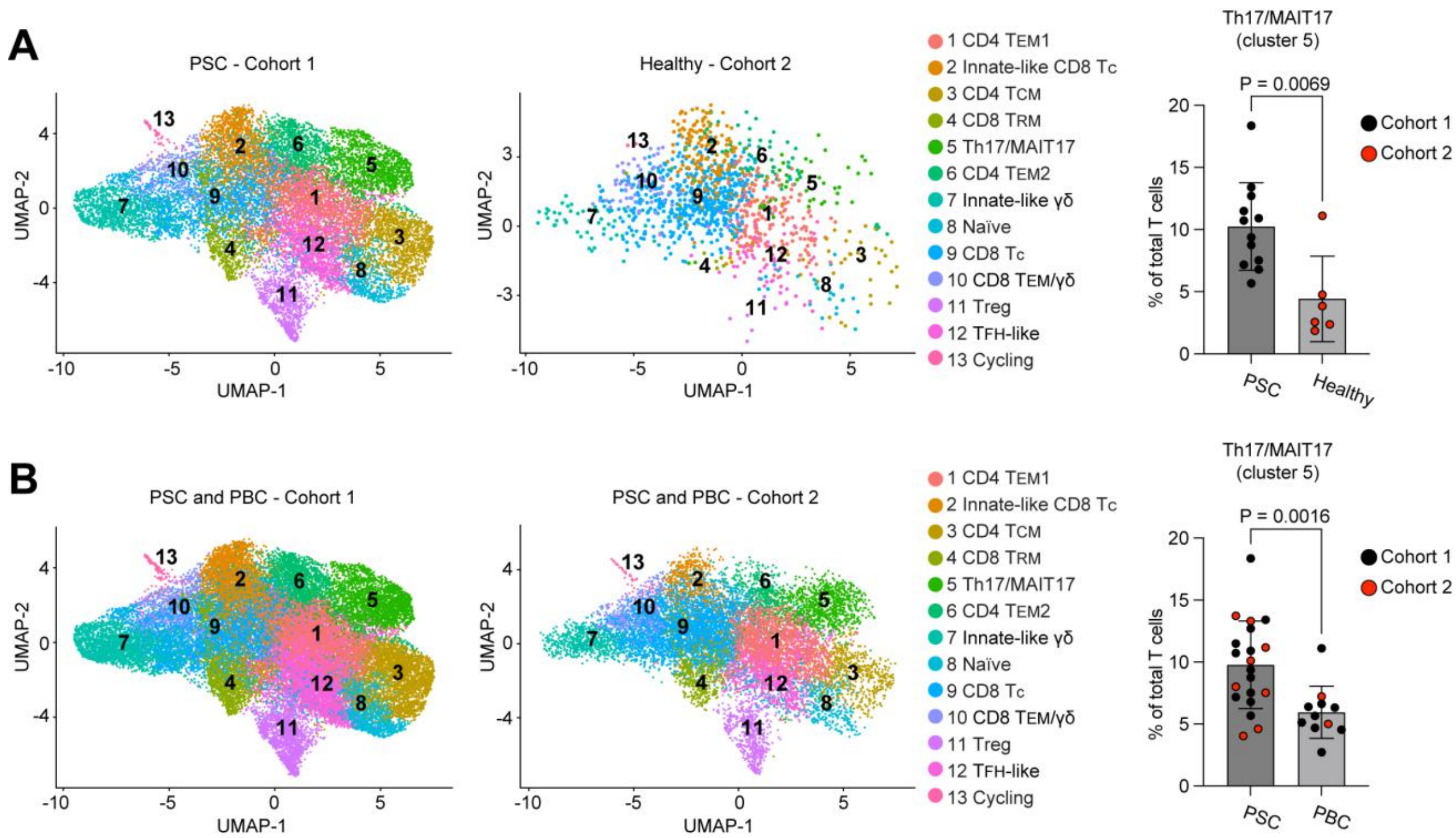

Supplementary Figure 5. PSC livers have increased Th17/MAIT17 proportions compared to healthy and PBC controls. (A) UMAPs indicate clustering of T cells from 12 PSC livers (Supplementary Table 1) herein and 6 healthy controls from Andrews and Nakib *et al*.[20] assessed by scRNA-seq (Cohort 1). Bar graph shows Th17/MAIT17 proportion as percentage of total T cells. (B) UMAPs indicate clustering of PSC and PBC liver T cells (12 PSC, 9 PBC; Supplementary Table 1) and Andrews and Nakib *et al*.[20] (8 PSC, 2 PBC) assessed by scRNA-seq (Cohort 2). Bar graph shows Th17/MAIT17 proportion as percentage of total T cells. Statistical significance evaluated using Mann-Whitney.

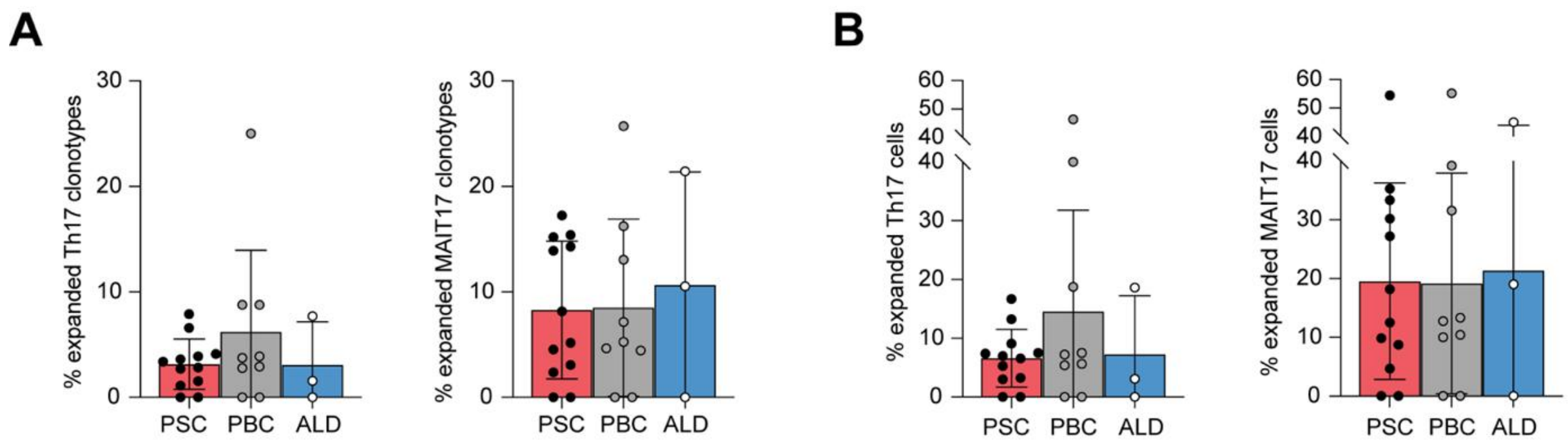

Supplementary Figure 6. Clonal expansion of Th17 and MAIT17 enrichment in PSC. (A) Percentage of expanded Th17 and MAIT17 clonotypes. (B) Percentage of expanded Th17 and MAIT17 cells.

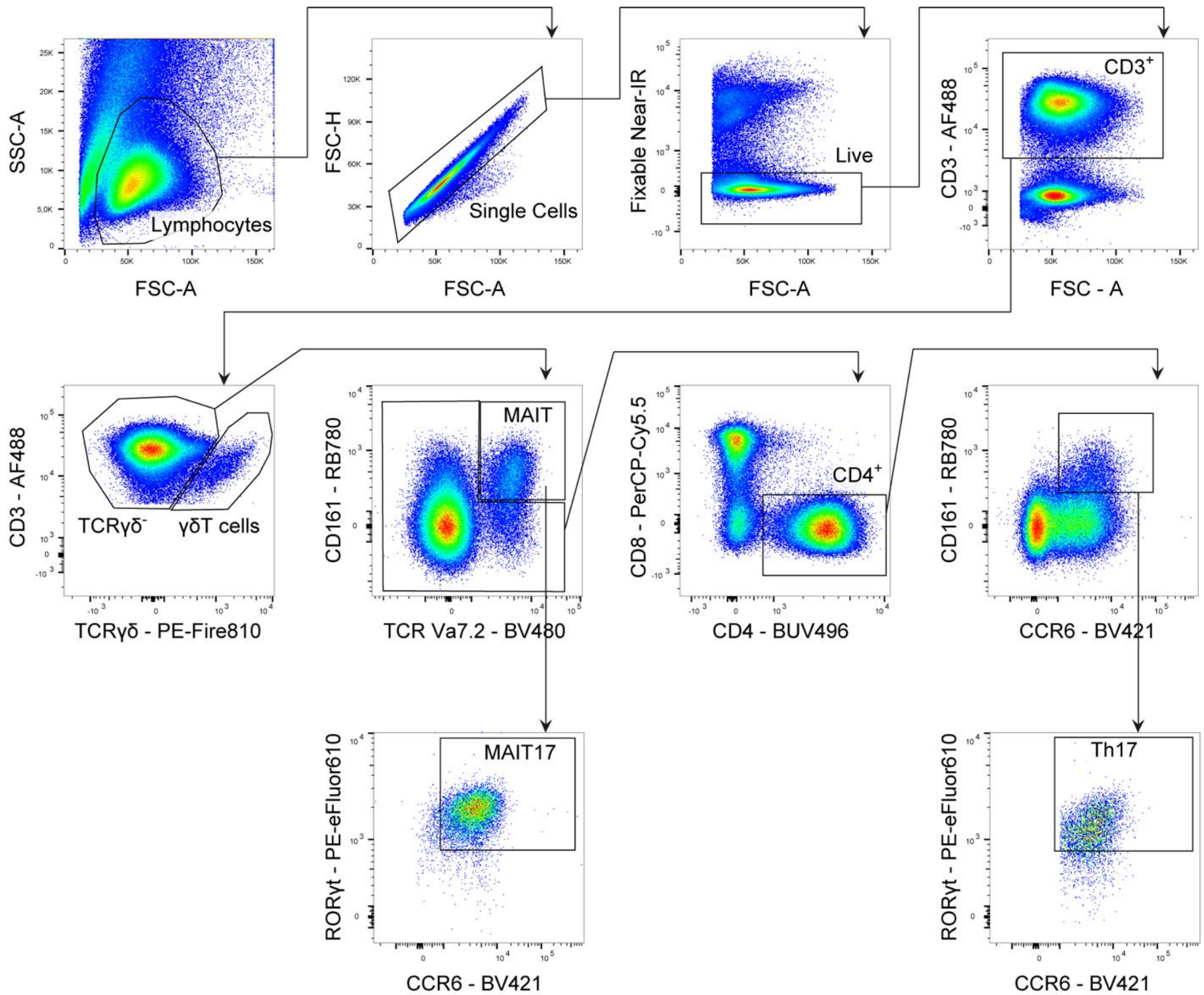

Supplementary Figure 7. Gating strategy for the identification of liver MAIT17 and Th17 cells by flow cytometry. Cryopreserved LMCs stained for MAIT17 and Th17 markers were first gated on lymphocyte morphology and single cells using forward (FSC) and side scatter (SSC). Dead cells were excluded using Fixable Near-IR and TCR$\gamma\delta^+$ cells were excluded from live CD3E$^+$TCR$\alpha\beta^+$ cells. MAIT cells within live CD3E$^+$TCR$\alpha\beta^+$ cells were identified as CD161$^+$TCRVα7.2$^+$ and defined as MAIT17 by RORγt$^+$ and CCR6$^+$ co-staining. TCRVα7.2$^-$ cells positive for CD4, CD161 and RORγt were enumerated as Th17 cells.

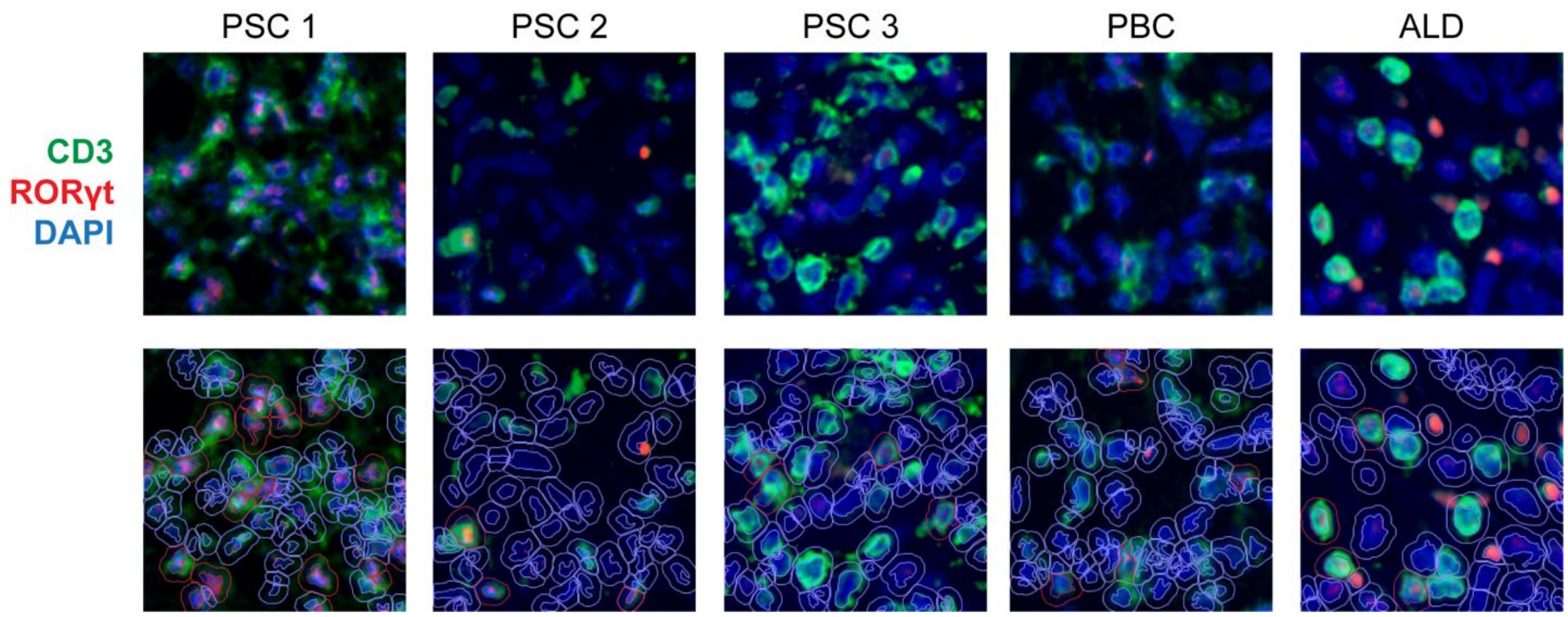

Supplementary Figure 8. Immunofluorescence and quantification of Th17/MAIT17 cells in liver tissue from PSC and disease controls. Images in top row indicate $CD3^+$ (green), nuclear $ROR\gamma t^+$ (red) and $DAPI^+$ nuclei (blue) IF staining of liver tissue from 3 PSC, 2 PBC and 1 ALD explant. For each liver section, numbers of single positive ($ROR\gamma t^+$) and double-positive ($CD3^+ROR\gamma t^+$) cells were quantified for 3 regions (150 μm^2). Quantification was performed in QuPath v0.5[18] using the train object classifier set to random trees. To train the classifier, 5-15 negative, single positive or double-positive cells were manually annotated. Within each region, T cell percentages of total $ROR\gamma t^+$ were calculated by dividing the number of double-positive on the number of single-positive. Mean T cell percentages of total $ROR\gamma t^+$ cells for 3 regions was then average for each sample.

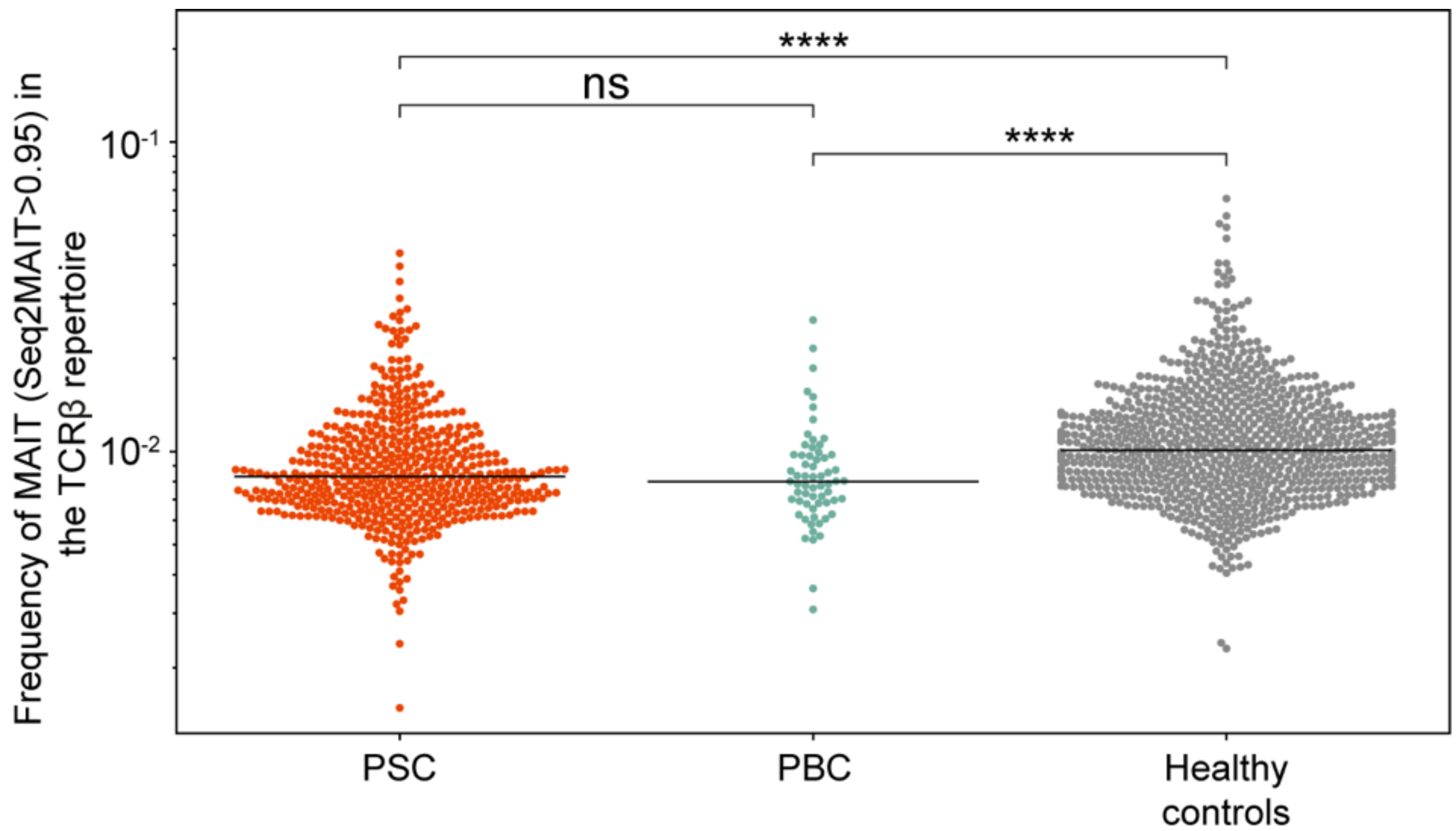

Supplementary Figure 9. Predicted MAIT cell clonotype frequencies in blood PSC, PBC and healthy controls. Fraction of MAIT TCR clonotypes in blood of 504 PSC, 63 PBC and 904 healthy controls predicted by Seq2MAIT. Statistical significance was evaluated by Mann-Whitney test in Python using statannotions package, ns > 0.05, ****$p \leq 0.0001$.

Supplementary Table 1. Clinical details of scRNA-seq cohort at liver transplantation

Patient ID	Disease	Age (years)	Sex (M/F)	IBD status	UDCA (Y/N)	Disease stage	APRI	FIB-4	MELD Na	Hb (g/dL)	Platelets (10^9/L)	Leuko (10^9/L)	INR
1	PSC	38	M	UC	Y	late	0.8	1.4	7	14.3	220	5.4	0.9
2	PSC	65	F	CD	N	late	21.1	31.1	25	8.4	36	21.3	1.3
3	PSC	37	M	—	Y	late	4.7	8.6	19	11.2	86	4.7	1.2
4	PSC	44	M	UC	Y	early	1.1	4.6	15	12.6	218	10.2	1.3
5	PSC	19	M	CD	Y	early	1.7	1.1	14	11.2	174	4.5	1.2
6	PSC	33	M	UC	N	late	2.5	4.7	23	9.0	159	5.0	1.4
7	PSC	56	M	UC	N	late	9.4	14.8	21	13.0	102	5.5	1.6
8	PSC	63	M	—	Y	early	1.0	4.3	11	12.7	123	6.3	1.1
9	PSC	36	M	UC	N	late	2.2	1.9	18	13.9	216	5.5	1.3
10	PSC	70	M	UC	N	late	0.6	3.5	10	11.7	121	4.9	1.2
11	PSC	28	F	UC	N	early	0.7	0.8	25	12.1	290	5.7	1.1
12	PSC	22	M	UC	Y	early	1.2	0.6	14	13.3	375	9.1	1.1
13	PBC	45	F	—	N	late	2.0	5.1	12	14.1	74	10.6	1.1
14	PBC	54	F	—	Y	late	3.1	8.4	19	13.2	90	4.2	1.4
15	PBC	46	F	—	N	late	3.4	7.9	21	10.3	115	4.0	1.6
16	PBC	44	M	—	N	late	1.4	1.8	9	12.8	296	6.0	0.9
17	PBC	43	F	—	Y	early	1.7	5.2	10	10.2	96	5.7	1.1
18	PBC	57	M	—	N	late	2.3	5.8	11	14.5	155	3.6	1.1
19	PBC	69	F	—	Y	late	0.9	4.8	15	7.7	139	4.3	1.3
20	PBC	52	M	—	N	late	3.7	8.2	7	11.9	46	5.2	1.1
21	PBC	53	F	—	Y	late	3.9	12.5	12	9.2	66	3.5	1.2
22	ALD	55	F	—	N	late	1.2	4.4	19	13.4	112	8.2	1.7
23	ALD	62	F	—	N	late	0.5	2.0	10	13.1	159	8.9	1.4
24	ALD	56	F	—	N	late	1.4	5.3	19	10.0	83	4.8	1.5

Patient ID	Disease	Sodium (mmol/L)	AST (U/L)	ALT (U/L)	Protein (g/L)	ALB (g/dL)	bilirubin (μmol/L)	CRP (mg/L)	γGT (U/L)	ALP (U/L)	Creatinine (μmol/L)	Dialysis (Y/N)
1	PSC	141	87	119	82	43	19	1.2	444	272	82	N
2	PSC	136	304	311	39	32	916	51.0	313	351	52	N
3	PSC	132	200	100	70	32	90	46.0	450	665	71	N
4	PSC	140	97	18	99	36	73	21.0	1689	339	52	N
5	PSC	138	116	138	69	39	73	3.0	186	176	69	N
6	PSC	137	162	51	79	32	409	32.0	88	286	101	N
7	PSC	138	384	202	62	30	222	28.0	213	231	69	N
8	PSC	143	50	35	71	33	25	33.0	93	393	113	N
9	PSC	139	187	267	72	49	149	4.4	440	1110	88	N
10	PSC	139	28	21	80	40	8	8.0	277	233	104	N
11	PSC	133	79	103	67	41	34	1.0	529	252	51	Y
12	PSC	142	176	263	80	44	86	3.0	698	541	65	N
13	PBC	141	59	49	75	39	54	3.1	377	328	52	N
14	PBC	135	111	63	72	34	104	28.0	74	199	80	N
15	PBC	136	157	64	66	26	166	3.9	119	598	49	N
16	PBC	139	171	192	79	42	35	1.6	523	1776	67	N
17	PBC	137	64	30	80	35	36	13.8	65	190	77	N
18	PBC	138	143	81	86	40	44	13.8	456	620	89	N
19	PBC	134	52	29	51	21	36	15.1	53	159	77	N
20	PBC	135	68	87	69	40	11	3.0	286	159	70	N
21	PBC	139	103	44	56	34	48	26.8	368	386	50	N
22	ALD	137	55	38	63	33	102	4.9	35	200	60	N
23	ALD	130	26	26	77	40	18	21.0	274	137	65	N
24	ALD	137	47	36	68	39	14	1.6	194	120	207	N

Supplementary Table 1 Abbreviations: ALB, albumin; ALD, alcohol-related liver disease; ALT, alanine aminotransferase; ALP, alkaline phosphatase; APRI, aspartate aminotransferase to platelet ratio index; AST, aspartate transaminase; CD, Crohn's disease; CRP, C-reactive protein; FIB-4, fibrosis-4 index; γGT, gamma-glutamyl transferase; Hb, hemoglobin; IBD, inflammatory bowel disease; INR, international normalized ratio; leuko, leukocytes; MELD-Na, model for end-stage liver disease sodium, neg, negative; PBC, primary biliary cholangitis; PSC, primary sclerosing cholangitis; UC, ulcerative colitis; UDCA, ursodeoxycholic acid; (–), no result; early-stage disease = FIB-4 < 1.3 for < 65 years, < 2.0 for ≥ 65 years; later-stage disease = FIB-4 ≥ 1.3 for < 65 years, ≥ 2.0 for ≥ 65 years.

Supplementary Table 2. Clinical details of IHC liver staining cohort at liver transplantation

Patient ID	Disease	Age (years)	Sex (M/F)	IBD status	UDCA (Y/N)	Disease Stage	APRI	FIB-4	MELD Na	Hb (g/dL)	Platelets (10^9/L)	Leuko (10^9/L)	INR
1	PSC	38	M	UC	Y	late	0.8	1.4	7	14.3	220	5.4	0.9
3	PSC	37	M	neg	Y	late	4.7	8.6	19	11.2	86	4.7	1.2
14	PBC	54	F	neg	Y	late	3.1	8.4	20	13.2	90	4.2	1.4
23	ALD	62	F	neg	N	late	0.5	2.0	10	13.1	159	8.9	1.4
25	PSC	56	M	neg	N	early	0.2	0.7	6	13.4	366	8.5	1.0
26	PSC	41	M	UC	N	late	0.8	1.4	8	13.4	287	8.9	1.2
27	PSC	43	M	neg	Y	late	1.2	1.9	20	15.8	195	5.6	1.1
28	PSC	42	M	UC	N	early	0.2	0.6	7	15.5	348	6.7	1.1
29	PSC	40	M	UC	Y	late	0.8	1.4	18	14.9	305	7.5	1.1
30	PSC	53	M	UC	Y	late	1.0	1.7	9	13.8	282	8.0	1.0
31	PSC	32	M	UC	Y	late	4.5	6.4	7	12.2	60	2.2	1.0
32	PSC	48	F	neg	Y	early	0.5	0.9	12	13.5	445	3.5	1.1
33	PSC	33	F	UC	Y	late	5.1	6.2	22	10.4	68	3.6	1.4
34	PSC	58	M	neg	N	late	2.6	8.3	22	12.2	81	4.0	1.5
35	PSC	47	F	UC	N	late	5.2	5.9	24	12.0	148	7.5	1.6
36	PSC	32	M	UC	N	late	7.7	11.0	15	10.1	72	2.1	1.2
37	PSC	66	M	neg	N	late	1.5	4.4	15	13.1	153	7.8	1.1
38	PSC	67	M	UC	Y	late	1.9	7.2	19	11.6	184	6.4	1.2
39	PSC	29	M	UC	Y	late	1.2	1.7	24	8.9	155	4.4	1.5
40	PSC	39	M	UC	N	early	0.2	0.4	6	15.3	466	14.7	1.0
41	PSC	55	F	UC	Y	late	1.1	1.8	7	12.9	246	7.9	1.1
42	PSC	54	F	neg	N	late	0.7	1.3	9	11.8	210	9.4	0.9
43	MASH	64	F	neg	N	late	1.2	3.9	8	9.5	141	6.8	1.2
44	MASH	55	M	neg	N	late	0.6	2.7	11	12.2	150	7.3	1.3
45	MASH	70	M	neg	N	late	0.4	2.1	8	8.6	210	5.2	1.2
46	ALD	70	M	neg	N	late	2.0	16.5	29	8.6	36	3.1	2.5
47	ALD	58	M	neg	N	late	0.7	4.8	11	11.7	106	6.2	1.3
48	ALD	56	M	neg	N	late	1.6	5.6	18	10.4	138	7.3	1.2
49	PBC	66	F	neg	N	late	1.6	7.0	15	8.0	68	1.4	1.3
50	PBC	72	F	neg	N	late	0.8	4.4	23	10.4	82	9.0	2.1
51	PBC	58	F	neg	N	late	6.9	9.5	17	9.7	98	4.7	1.2
52	PBC	72	F	neg	Y	late	2.1	8.2	14	11.9	91	3.3	1.3
53	PBC	53	M	neg	Y	late	2.1	5.0	25	9.5	173	7.2	1.7
54	PBC	56	M	neg	N	late	3.0	4.7	16	12.7	138	3.9	0.9
55	PBC	64	F	neg	Y	late	15.1	23.5	30	7.6	48	4.6	2.2
56	PBC	70	F	neg	Y	late	1.5	5.2	21	10.6	137	7.3	1.7
57	PBC	71	F	neg	Y	late	1.8	6.6	15	10.8	125	4.2	1.3

Patient ID	Disease	Sodium (mmol/L)	AST (U/L)	ALT (U/L)	Protein (g/L)	ALB (g/dL)	bilirubin (μmol/L)	CRP (mg/L)	γGT (U/L)	ALP (U/L)	Creatinine (μmol/L)	Dialysis (Y/N)
1	PSC	141	87	119	82	43	19	1.2	444	272	82	N
3	PSC	132	200	100	70	32	90	46.0	450	665	71	N
14	PBC	135	111	63	72	34	104	28.0	74	199	80	N
23	ALD	130	26	26	77	40	18	21.0	274	137	65	N
25	PSC	140	43	84	72	45	7	0.6	100	134	67	N
26	PSC	143	110	119	70	46	14	1.1	537	255	82	N
27	PSC	140	113	179	76	48	458	0.7	306	166	81	N
28	PSC	139	30	37	75	47	7	1.2	30	73	68	N
29	PSC	137	120	120	83	45	279	50.0	292	505	77	N
30	PSC	145	143	264	74	44	37	4.6	1107	528	69	N
31	PSC	140	136	128	68	38	20	3.1	236	344	64	N
32	PSC	144	79	82	69	43	55	1.7	229	325	66	N
33	PSC	134	121	89	52	27	248	23.0	30	281	53	N
34	PSC	132	107	85	74	22	122	17.0	137	188	56	N
35	PSC	136	271	214	67	31	326	16.0	277	663	61	N
36	PSC	140	278	127	79	35	87	14.0	1123	722	50	N
37	PSC	134	111	117	87	35	30	1.0	500	188	108	N
38	PSC	136	179	82	79	36	100	8.9	250	494	116	N
39	PSC	131	90	102	95	27	189	65.0	153	346	33	N
40	PSC	135	40	61	80	41	10	72.0	478	407	68	N
41	PSC	143	93	132	82	44	16	10.0	832	393	51	N
42	PSC	139	54	115	75	41	14	5.7	1411	613	119	N
43	MASH	135	59	48	78	41	9	4.0	186	74	76	N
44	MASH	136	46	38	54	26	22	28.0	73	112	95	N
45	MASH	127	37	33	74	36	12	3.7	190	89	79	N
46	ALD	137	36	18	55	44	98	12.0	56	89	167	N
47	ALD	135	39	20	59	31	29	13.0	172	137	103	N
48	ALD	132	112	66	70	32	24	18.0	164	143	140	N
49	PBC	132	33	21	62	35	22	12.0	97	168	120	N
50	PBC	130	23	21	49	34	38	67.0	11	37	41	N
51	PBC	138	237	216	81	31	120	16.0	379	598	52	N
52	PBC	139	67	42	71	26	55	24.0	79	212	84	N
53	PBC	130	162	98	85	17	147	17.0	73	252	75	N
54	PBC	137	188	260	80	43	47	4.0	1261	699	134	N
55	PBC	138	254	159	53	38	680	28.0	24	91	56	N
56	PBC	131	71	48	53	32	115	13.0	38	165	56	N
57	PBC	139	80	48	60	23	52	42.0	57	441	92	N

Supplementary Table 2 Abbreviations: ALB, albumin; ALD, alcohol-related liver disease; ALT, alanine aminotransferase; ALP, alkaline phosphatase; APRI, aspartate aminotransferase to platelet ratio index; AST, aspartate transaminase; CRP, C-reactive protein; FIB-4, fibrosis-4 index; γGT, gamma-glutamyl transferase; Hb, hemoglobin; IBD, inflammatory bowel disease; INR,

international normalized ratio; leuko, leukocytes; MASH, metabolic dysfunction-associated steatohepatitis; MELD-Na, model for end-stage liver disease sodium, neg, negative; PBC, primary biliary cholangitis; PSC, primary sclerosing cholangitis; UC, ulcerative colitis; UDCA, ursodeoxycholic acid; early-stage disease = FIB-4 < 1.3 for < 65 years, < 2.0 for ≥ 65 years; later-stage disease = FIB-4 ≥ 1.3 for < 65 years, ≥ 2.0 for ≥ 65 years.

MAIT17 TCR reporter line	TCR chain	V-gene	CDR3 Sequence	J-gene
MAIT17.1	Alpha	*TRAV1-2*	CAVRDSNYQLIW	*TRAJ33*
	Beta	*TRBV6-5*	CASSPTENTEAFF	*TRBJ1-1*
MAIT17.2	Alpha	*TRAV1-2*	CAVMDSNYQLIW	*TRAJ33*
	Beta	*TRBV6-2*	CASSYRGGSGANVLTF	*TRBJ2-6*
MAIT17.3	Alpha	*TRAV1-2*	CAVMDSSYKLIF	*TRAJ12*
	Beta	*TRBV6-1*	CASTSSEVGEQYF	*TRBJ2-7*
MAIT17.4	Alpha	*TRAV1-2*	CAVKDSNYQLIW	*TRAJ33*
	Beta	*TRBV20-1*	CSQTSGRINEQFF	*TRBJ2-1*
MAIT17.5	Alpha	*TRAV1-2*	CAVLDSNYQLIW	*TRAJ33*
	Beta	*TRBV3-1*	CASSRSGSETDTQYF	*TRBJ2-3*
MAIT17.6	Alpha	*TRAV1-2*	CAALDSNYQLIW	*TRAJ33*
	Beta	*TRBV15*	CATSRTTGEGYNEQFF	*TRBJ2-1*
MAIT17.7	Alpha	*TRAV1-2*	CAPMDSNYQLIW	*TRAJ33*
	Beta	*TRBV6-1*	CASSELAGADTQYF	*TRBJ2-3*
MAIT17.8	Alpha	*TRAV1-1*	CAVRDFPPNSGYSTLTF	*TRAJ11*
	Beta	*TRBV6-6*	CASSYSEQRSNQPQHF	*TRBJ1-5*
MAIT17.9	Alpha	*TRAV29/DV5*	CAASSGNTGKLIF	*TRAJ37*
	Beta	*TRBV7-9*	CASSLLRDLGGYTF	*TRBJ1-2*
MAIT17.10	Alpha	*TRAV13-1*	CAVLNRDDKIIF	*TRAJ30*
	Beta	*TRBV6-1*	CASSEGLEAYNIQYF	*TRBJ2-4*

Supplementary Table 3. MAIT17 TCR sequences identified by TCR-seq used to generate MAIT17 reporters. TCR reporter lines with prototypic MAIT gene usage (*TRAV1-2*$^+$*TRAJ33*$^+$/*TRAJ12*$^+$) shown in purple. MAIT17-like TCR reporter lines (*TRAV1-2*$^-$*TRAJ33*$^-$/*TRAJ12*$^-$) shown in orange.
