# Supplementary material for: Intrahepatic T cell analysis reveals Th17 and MAIT17 enrichment in primary sclerosing cholangitis
Source: JHEP Rep. 2026 Apr 17;8(9):101857. doi: 10.1016/j.jhepr.2026.101857 (PMC13507853; doi:10.1016/j.jhepr.2026.101857)
Supplement: Supplementary file 4 [file mmc2.docx]

**JHEP Reports**

**CTAT methods**

Tables for a “Complete, Transparent, Accurate and Timely account” (CTAT) are now mandatory for all revised submissions. The aim is to enhance the reproducibility of methods.

- Only include the parts relevant to your study
- Refer to the CTAT in the main text as ‘Supplementary CTAT Table’
- Do not add subheadings
- Add as many rows as needed to include all information
- Only include one item per row

**If the CTAT form is not relevant to your study, please outline the reasons why:**

1.1 **Antibodies**

| **Name** | **Citation** | **Supplier** | **Cat no.** | **Clone no.** |
| --- | --- | --- | --- | --- |
| CCR6 |  | BioLegend | 353407 | G034E3 |
| CCR7 |  | Thermo Fisher Scientific | 417-1979-41 | 3D12 |
| CD3 |  | Thermo Fisher Scientific | 53-0037-41 | OKT3 |
| TCR γ/δ |  | BioLegend | 331243 | B1 |
| CD161 |  | BD Biosciences | 569114 | DX12 |
| TCR Vα7.2 |  | BD Biosciences | 749493 | OF-5A12 |
| CD8a |  | BioLegend | 301032 | RPA-T8 |
| CD4 |  | Thermo Fisher Scientific | 364-0049-42 | RPA-T4 |
| CD45RA |  | Thermo Fisher Scientific | 363-0458-41 | HI100 |
| CD25 |  | Thermo Fisher Scientific | H052T03R03-A | BC96 |
| PD-1 |  | Thermo Fisher Scientific | 416-2799-41 | J105 |
| ICOS |  | Thermo Fisher Scientific | 63-9948-42 | ISA-3 |
| RORγt |  | Thermo Fisher Scientific | 61-6988-80 | AFKJS-9 |
| FOXP3 |  | Thermo Fisher Scientific | 25-4776-41 | PCH101 |
| CTLA-4 |  | Thermo Fisher Scientific | 367-1529-41 | 14D3 |
| FGFBP2 |  | BioLegend | 346603 | TDA3 |
| Bcl-6 |  | BD Biosciences | 567180 | K112-91 |
| TCR β chain |  | BioLegend | 109212 | H57-597 |
| MR1 |  | BioLegend | 361108 | 26.5 |
| RORγt |  | Sigma-Aldrich | MABF81 | 6F3.1 |
| CD3 |  | Abcam, Cambridge, UK | ab135372 | SP16 |
| ImmPRESS HRP Goat Anti-Mouse IgG Polymer Detection Kit, Peroxidase |  | Vector Laboratories, Newark, USA | MP-7452 |  |
| ImmPRESS HRP Horse Anti-Rabbit IgG Polymer Detection Kit, Peroxidase |  | Vector Laboratories, Newark, USA | MP-7401 |  |
| Zombie NIR™ Fixable Viability Kit |  | BioLegend | 423106 |  |
| 7-AAD (7-Aminoactinomycin D) |  | Thermo Fisher Scientific | A1310 |  |
| MR1:5-OP-RU tetramer |  | NIH Tetramer Core, Emory University, Atlanta, USA |  |  |
| MR1:6-formylpterin tetramer |  | NIH Tetramer Core, Emory University, Atlanta, USA |  |  |
| Purified Mouse IgG2a, κ Isotype Ctrl Antibody |  | BioLegend | 400202 | MOPC-173 |
| Rabbit IgG, monoclonal [EPR25A] - Isotype Control |  | Abcam | ab172730 | EPR25A |
| Donkey anti-Mouse IgG (H+L) Highly Cross-Adsorbed Secondary Antibody, Alexa Fluor 555 |  | Thermo Fisher Scientific | A31570 |  |
| Goat anti-Rabbit IgG (H+L) Cross-Adsorbed Secondary Antibody, Alexa Fluor 647 |  | Thermo Fisher Scientific | A21244 |  |

1.2 **Cell lines**

| **Name** | **Citation** | **Supplier** | **Cat no.** | **Passage no.** | **Authentication test method** |
| --- | --- | --- | --- | --- | --- |
| J76-triple parameter reporters | PMID: 33251011  PMID: 26780292 | Peter Steinberger, Institute of Immunology, Medical University of Vienna, Lazarettgasse 19, 1090 Vienna, Vienna, Austria.  E-mail: peter.steinberger@meduniwien.ac.at | NA | - |  |
| H69 (human cholangiocyte cell line) | PMID: 8023938 | Douglas M. Jefferson, Graduate School of Biomedical Sciences, Genetics, Molecular and Cellular Biology, 136 Harrison Avenue, Boston, MA, 02111, USA | NA | - |  |

1.3 **Organisms**

| **Name** | **Citation** | **Supplier** | **Strain** | **Sex** | **Age** | **Overall n number** |
| --- | --- | --- | --- | --- | --- | --- |
|  |  |  |  |  |  |  |

1.4 **Sequence based reagents**

| **Name** | **Sequence** | **Supplier** |
| --- | --- | --- |
|  |  |  |

1.5 **Biological samples**

| **Description** | **Source** | **Identifier** |
| --- | --- | --- |
| Human liver mononuclear cell suspensions | 1) Norwegian PSC Research Center, Department of Transplantation Medicine, Division of Surgery and Specialized Medicine, Oslo University Hospital Rikshospitalet, Oslo, Norway  2) Liver Unit, University Hospitals Birmingham National Health Service Foundation Trust Queen Elizabeth, Birmingham, UK |  |
| Human blood | Institute of Clinical Molecular Biology, University of Kiel and University Hospital Schleswig-Holstein (UKSH), Kiel, Germany | PMID: 40500415 |
| Human liver tissues | Norwegian PSC Research Center, Department of Transplantation Medicine, Division of Surgery and Specialized Medicine, Oslo University Hospital Rikshospitalet, Oslo, Norway |  |

1.6 **Deposited data**

| **Name of repository** | **Identifier** | **Link** |
| --- | --- | --- |
| RIIM Data Explorer | NoPSC Liver Atlas - Single Cell T-Cell Profiling | https://riim-data.no/portal.html?dataset=NoPSC_LIVER_ATLAS/TCELLS |
| CELLxGENE Discover - Single-cell and spatial transcriptomics characterisation of the immunological landscape in the healthy and PSC human liver | All cells types from scRNA-seq of human primary sclerosing cholangitis patients and healthy controls | https://datasets.cellxgene.cziscience.com/7d4d0da4-655e-438a-a2ec-b4371e2b80fc.h5ad |

1.7 **Software**

| **Software name** | **Manufacturer** | **Version** |
| --- | --- | --- |
| Cell Ranger | 10x Genomics, Pleasanton, USA | Version 7.1.0 |
| R | Posit PBC, Boston MA, USA | Version 4.3.2 |
| GraphPad Prism | GraphPad, San Diego, USA | Version 10.0.3 |
| Sony ID7000 software | Sony Biotechnology, San Jose, USA | Version 2.0.2.17121 |
| FlowJo | BD Life Sciences, Franklin Lakes, New Jersey, USA | Version 10.10.0 |
| QuPath | PMID: 29203879 | Version 0.5.1 |

1.8 **Other (*e.g*. drugs, proteins, vectors etc.)**

| EasySep^TM^ Human T Cell Enrichment Cocktail | STEMCELL Technologies, Vancouver, Canada | Cat no: 19051C.2 |
| --- | --- | --- |
| EasySep^TM^ D Magnetic Particles | STEMCELL Technologies, Vancouver, Canada | Cat no: 19250 |
| 5-amino-6-D-ribitylaminouracil | MuseChem, Paoli, Pennsylvania, USA | Cat no: 17014-74-3 |
| Methylglyoxal | Sigma-Aldrich, St. Louis, USA | Cat no: M0252 |
| MAIT17.1 | VectorBuilder, Chicago, Illinois, USA | Vector ID: VB240220-1355dtg |
| MAIT17.2 | VectorBuilder | Vector ID: VB240220-1357tmh |
| MAIT17.3 | VectorBuilder | Vector ID: VB240220-1358gyb |
| MAIT17.4 | VectorBuilder | Vector ID: VB240220-1359cvh |
| MAIT17.5 | VectorBuilder | Vector ID: VB240220-1360fjj |
| MAIT17.6 | VectorBuilder | Vector ID: VB240220-1363jab |
| MAIT17.7 | VectorBuilder | Vector ID: VB240220-1365zgq |
| MAIT17.8 | VectorBuilder | Vector ID: VB240220-1366nzk |
| MAIT17.9 | VectorBuilder | Vector ID: VB240220-1367qzj |
| MAIT17.10 | VectorBuilder | Vector ID: VB240220-1368gpb |
| TCR-mut-p53-13:01 | VectorBuilder | Vector ID: VB230702-1028xgu |

1.9 **Please provide the details of the corresponding methods author for the manuscript:**

Lisa R. V. Brynjulfsen

Norwegian PSC Research Center

Research Institute of Internal Medicine

Division of Surgery and Specialized Medicine

Oslo University Hospital Rikshospitalet

Postboks 4950 Nydalen, N-0424 Oslo, Norway

Phone: +47 401 04 126

Email: l.r.v.brynjulfsen@medisin.uio.no

**2.0	Please confirm for randomised controlled trials all versions of the clinical protocol are included in the submission. These will be published online as supplementary information.**

NA
